# Infrared Spectrum of the Adduct 2-Chloro-2-hydroperoxybut-3-ene [(C_2_H_3_)CCl(CH_3_)OOH] of the Reaction between the Criegee Intermediate Methyl Vinyl Ketone Oxide [C_2_H_3_C(CH_3_)OO] and HCl

**DOI:** 10.1021/acs.jpca.4c04936

**Published:** 2024-09-26

**Authors:** Hao Wang, Yuan-Pern Lee

**Affiliations:** † Department of Applied Chemistry and Institute of Molecular Science, National Yang Ming Chiao Tung University, Hsinchu 300093, Taiwan; ‡ Center for Emergent Functional Matter Science, National Yang Ming Chiao Tung University, Hsinchu 300093, Taiwan

## Abstract

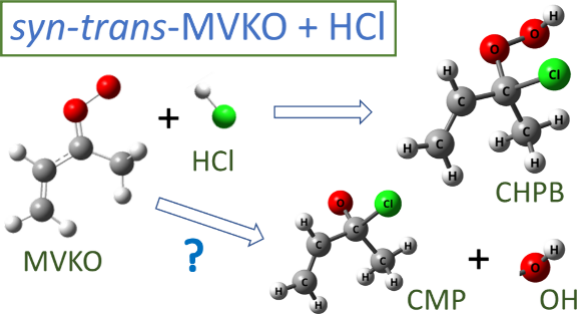

Methyl vinyl ketone oxide (MVKO, C_2_H_3_C(CH_3_)OO) is an important Criegee intermediate produced from ozonolysis of isoprene, which is the most abundant nonmethane hydrocarbon emitted into the atmosphere. Reactions between Criegee intermediate and hydrogen chloride (HCl) are important because of their large rate coefficients. In this work, we photolyzed a mixture of (*Z*)-(CH_2_I)HC=C(CH_3_)I/HCl/O_2_ at 248 nm to produce MVKO to carry out the reaction MVKO + HCl and recorded infrared spectra of transient species with a step-scan Fourier-transform infrared absorption spectrometer. Eleven bands near 1415, 1381, 1350, 1249, 1178, 1118, 1103, 1065, 978, 931, and 895 cm^–1^ were observed and assigned to the hydrogen-transferred adduct (C_2_H_3_)CCl(CH_3_)OOH (2-chloro-2-hydroperoxybut-3-ene, CHPB) according to the predicted IR spectrum using the B3LYP/aug-cc-pVTZ method; the conformation could not be definitively determined. According to calculations, most low-energy conformers can interconvert, and the most stable conformers *anti*–*trans*-CHPB and *syn*–*trans*-CHPB might have the major contributions. Four bands near 1423, 1360, 1220, and 1080 cm^–1^ were observed and tentatively assigned to the OH-decomposition products (C_2_H_3_)CCl(CH_3_)O (1-chloro-1-methyl-2-propenyloxy, CMP); both *trans*-CMP and *cis*-CMP might contribute. Unlike in the case of CH_2_OO + HCl and CH_3_CHOO + HCl, in which secondary reactions of the OH-decomposition products reacted readily with O_2_ to produce the dehydrated products HC(O)Cl and CH_3_C(O)Cl, respectively, the secondary reaction of CMP with O_2_ was not observed because there is no feasible H atom in CMP for O_2_ to abstract.

## 1 Introduction

Isoprene [CH_2_=CH–C(CH_3_)=CH_2_] has an estimated total global emission of about 600 Tg year^–1^ into Earth’s atmosphere; it is the second most abundantly emitted volatile organic compounds.^1,2^ Its major removal pathways in the atmosphere include reactions with OH, ozone (O_3_), and NO_3_.^3−7^ In the ozonolysis of isoprene, three types of Criegee intermediates, formaldehyde oxide (CH_2_OO), methacrolein oxide [MACRO, CH_2_=C(CH_3_)CHOO], and methyl vinyl ketone oxide [MVKO, C_2_H_3_C(CH_3_)OO], were produced, with estimated branching ratios ∼58, 19, and 23%, respectively.^6,8,9^ MVKO and MACRO are hence important Criegee intermediates in the atmosphere; their unique structures with resonance stabilization by the vinyl moiety affect their O–O bond strength and their reactivities.

For the simplest Criegee intermediate CH_2_OO, its reactions with many atmospheric species have been extensively studied. In contrast, the reactions of larger Criegee intermediates have been less explored. Typically, several conformers exist for the larger Criegee intermediates; different conformers might show distinctly diverse reactivities toward atmospheric species.^10,11^ For MVKO, four conformers exist; conformers *syn*–*trans*, *syn*–*cis*, *anti*–*trans*, and *anti*–*cis* are ordered by increasing energy, all within 13 kJ mol^–1^. The *syn*-/*anti*-notation specifies the orientation of the methyl moiety relative to the terminal O atom, and the *cis*-/*trans*-notation specifies the relative orientation of the C=C bond in the C_2_H_3_ moiety and the C=O bond. Barber et al. reported a novel method for the synthesis of MVKO by photoirradiation of a mixture of 1,3-diiodobut-2-ene [(CH_2_I)HC=C(CH_3_)I] and O_2_ to initiate the reaction C_2_H_3_C(CH_3_)I + O_2_ to form MVKO [C_2_H_3_C(CH_3_)OO] + I.^12^ These authors characterized *syn-* and *anti-*MVKO with near-infrared (NIR) action spectra by probing the decomposition product OH with laser-induced fluorescence; the NIR activation of MVKO in region 5750–6260 cm^–1^ resulted in the hydrogen transfer followed by dissociation to produce OH. Using the same production method, Vansco et al.,^13^ Caravan et al.,^14^ and Lin et al.^15,16^ reported the UV spectrum of MVKO. Endo et al. employed a discharged jet to produce MVKO and observed the microwave spectrum of only *syn*–*trans*-MVKO.^17^ We reported seven absorption bands of *syn*–*trans*-MVKO in the mid-infrared (IR) region; additional weak bands might be tentatively ascribed to *syn*–*cis*-MVKO.^18^ We observed also the IR spectrum of the iodoalkenyl radical C_2_H_3_C(CH_3_)I produced from photolysis of the precursor (CH_2_I)HC=C(CH_3_)I so that we recognized that photolysis caused the fission of the allylic C–I bond, not the vinylic C–I bond, of the precursor. At high pressure, the IR spectrum of the adduct C_2_H_3_C(CH_3_)IOO from C_2_H_3_C(CH_3_)OO + I was identified. The O–O stretching band of MVKO was observed at 948 cm^–1^, much greater than values 871 and 887 cm^–1^ observed for the corresponding bands of Criegee intermediates *syn*-CH_3_CHOO^19^ and (CH_3_)_2_COO,^20^ respectively, because the resonance stabilization of MVKO induced a stronger O–O bond. The unimolecular decomposition rate coefficient of *anti*-MVKO appeared to be smaller than that of *syn*-MVKO.^12,21^ Rate coefficients for the reactions of MVKO with SO_2_,^14,15^ HCOOH,^14^ H_2_O/(H_2_O)_2_,^14^ HNO_3_,^22^ (CH_3_)_2_S,^23^ and NH_2_C_3_H_6_OH^24^ have been reported.

Hydrogen chloride (HCl) has an average mixing ratio 0.53–2.7 ppbv (part per billion by volume) in the polluted urban atmosphere;^25^ its reaction with the Criegee intermediates might play an important role in atmospheric chemistry. The rate coefficient for the reaction of CH_2_OO with HCl was ∼4.7 × 10^–11^ cm^3^ molecule^–1^ s^–1^,^26,27^ in agreement with a value 4 × 10^–11^ cm^3^ molecule^–1^ s^–1^ predicted by Vereecken.^28^ Liu et al. probed OH by laser-induced fluorescence to monitor *syn*-CH_3_CHOO^29^ and determined the rate coefficient of the reaction *syn*-CH_3_CHOO + HCl to be (4.77 ± 0.95) × 10^–11^ cm^3^ molecule^–1^ s^–1^ at 298 K.^30^ In contrast, a value of (2.7 ± 1.0) × 10^–10^ cm^3^ molecule^–1^ s^–1^ was reported for the reaction *syn*-/*trans*-CH_3_CHOO + HCl at ∼70 Torr and 298 K by our group, indicating that *anti*-CH_3_CHOO reacts with HCl much faster than *syn*-CH_3_CHOO.^31^ The reactions of *syn*-CH_3_CHOO (and CH_2_OO) with HCl have effective first-order rate coefficients comparable to those of CH_2_OO with organic acids,^32^ implying their potential importance in atmospheric chemistry. To our knowledge, the rate coefficient and reaction products of MVKO + HCl have not been reported.

For the reaction products of CH_2_OO + HCl, Cabezas and Endo observed the microwave spectra of the H-transferred adduct *gauche*-chloromethyl hydroperoxide (CMHP, CH_2_ClOOH) in a discharged jet.^33^
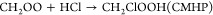
1Liang et al. in our laboratory identified the IR spectrum of *gauche*-CMHP using a step-scan Fourier-transform infrared (FTIR) spectrometer.^27^ These authors also pointed out that, although CH_2_ClO + OH are expected to be the major decomposition products of the adduct CH_2_ClOOH (reaction 2a), secondary reactions of CH_2_ClO with O_2_ to form HC(O)Cl + HO_2_ (reaction 3) and OH with HCl to form H_2_O + Cl (reaction 4) interfered because both HCl and O_2_ are major species in the system; instead, absorption bands of H_2_O and formyl chloride (HC(O)Cl) at 1782.9 cm^–1^ were observed.
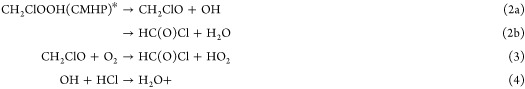


For the reaction products of CH_3_CHOO with HCl, Cabezas and Endo reported the microwave spectra of the H-transferred adduct of this reaction, *syn*- and *anti*-chloroethyl hydroperoxide (CEHP, CH_3_CHClOOH), in a discharged jet expansion.^34^
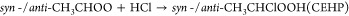
5An estimated population ratio of *anti*-CEHP/*syn*-CEHP = 3:1 was reported, indicating that *anti*-CH_3_CHOO reacts with HCl much faster than *syn*-CH_3_CHOO does, because *syn*-CH_3_CHOO was about five times more abundant than *anti*-CH_3_CHOO under their experimental conditions.^35,36^ In contrast, with the step-scan FTIR detection in our laboratory, only *anti*-CEHP was observed from *syn*-/*anti*-CH_3_CHOO + HCl reaction in a flowing cell at 298 K, indicating that the conversion from *syn*-CEHP to *anti*-CEHP of lower energy is facile.^31^ In addition, both H_2_O and acetyl chloride [CH_3_C(O)Cl, at 1819.1 cm^–1^] were observed as decomposition products of the H-transferred adduct CEHP, CH_3_CHClOOH (reaction 6b). According to temporal profiles of H_2_O and CH_3_C(O)Cl, some of them were produced from secondary reactions of a preferred set of the decomposition products of CEHP, CH_3_CHClO + OH (reaction 6a) via reactions of CH_3_CHClO with O_2_ (reaction 7) and OH with HCl (reaction 4).
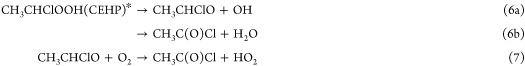


In this work, we extended our work to MVKO + HCl and observed infrared absorption bands of the H-transferred adduct 2-chloro-2-hydroperoxybut-3-ene [(C_2_H_3_)CCl(CH_3_)OOH, CHPB]. Four additional bands were tentatively assigned to the OH-decomposition products (C_2_H_3_)CCl(CH_3_)O (1-chloro-1-methyl-2-propenyloxy,CMP). Unlike those of CH_2_ClO (+ OH) produced from the reaction CH_2_OO + HCl and CH_3_CHClO (+ OH) from CH_3_CHOO + HCl that react with O_2_ readily, the secondary reaction of CMP with O_2_ was not observed, likely because there was no feasible H atom in CMP for O_2_ to abstract.

## 2 Methods

We employed a step-scan FTIR spectrometer (Bruker, Vertex 80v) to characterize the reaction products with IR absorption spectra, as described previously.^27,31,37^ A multipass White cell with a volume ∼1370 cm^3^ and an effective path length 15 × 24 = 360 cm served as both an absorption cell and a reactor; it was pumped with a dry screw pump (Hanbell PS80, 1600 L min^–1^). The photolysis laser is a KrF excimer laser (Coherent, CompexPro 102F, 248 nm, 7 Hz, ∼216 mJ pulse^–1^, beam size 8.4 × 1.2 cm^2^).

The IR beam from the FTIR entered and exited the reactor before being detected with a MCT (HgCdTe) detector at 77 K. The signal was recorded with an external 14-bit digitizer at 4 ns intervals and covered a total period of 40 μs. The signal was averaged over 15 laser shots at each scan step. Typically, to reach an improved ratio of signal-to-noise (S/N), six spectra were recorded and averaged under similar experimental conditions. To cover an extended period and also to achieve an improved S/N, an internal 24-bit digitizer with temporal resolution 12.5 μs was employed. To reduce the consumption of sample by reducing the total data-acquisition period, under-sampling was used; appropriate optical filters were employed to allow the passage of light only in the spectral region of interest. Two spectral ranges were employed: a range of 753–1504 cm^–1^ at resolution 1 cm^–1^ with 1523 scan steps were completed in ∼60 min and a range of 770–2200 cm^–1^ at resolution 2 cm^–1^ with 2538 scan steps were completed in ∼90 min. The full width at half-maximum, fwhm, after apodization with the Blackman-Harris 3-term function is 1.28 times the listed instrumental resolution, defined by the fwhm of the triangle apodization function.

Criegee intermediate C_2_H_3_C(CH_3_)OO (MVKO) were prepared by the reaction of O_2_ with C_2_H_3_C(CH_3_)I, which was produced on photolysis of the precursor (*Z*)-(CH_2_I)HC=C(CH_3_)I at 248 nm. The partial pressure of (*Z*)-(CH_2_I)HC=C(CH_3_)I was evaluated from the integrated-absorbance in region 1118–1205 cm^–1^ using a calculated IR cross section of 1.7 × 10^–17^ cm^2^ molecule^–1^. The partial pressures of the reactant HCl were determined by calibration of IR absorption with varied pressures. According to the UV absorption cross section of (*Z*)-(CH_2_I)HC=C(CH_3_)I, ∼ 1.94 × 10^–17^ cm^2^ molecule^–1^ at 248 nm,^24^ and the laser fluence, we estimated the photolysis fraction of C_2_H_3_C(CH_3_)I from (*Z*)-(CH_2_I)HC=C(CH_3_)I to be ∼46%, assuming a unity quantum yield. O_2_ was divided into two streams, one passed through liquid (*Z*)-(CH_2_I)HC=C(CH_3_)I before entering the reactor, the other was used to purge the windows. The total flow rate of O_2_, *F*_O2_, was ∼21 STP cm^3^ s^–1^, total pressure *P*_T_ = 80–85 Torr, and partial pressures of (*Z*)-(CH_2_I)HC=C(CH_3_)I and HCl were 25–27 mTorr and 25–30 mTorr, respectively. (*Z*)-(CH_2_I)HC=C(CH_3_)I (>95%, Accela ChemBio), HCl (99.999%, Ching-Fong), and O_2_ (99.99%, Chiah-Lung) were used as received.

The optimized geometries, harmonic (anharmonic) vibrational wavenumbers, zero-point vibrational energies (ZPVE), IR intensities, and rotational constants of all species discussed in this work were calculated with the B3LYP density-functional theory (DFT) using the Becke’s three-parameter hybrid exchange functional with a correlation functional of Lee et al.^38−40^ and Dunning’s correlation consistent polarized-valence triple-ζ basis set, augmented with s, p, d, and f functions (aug-cc-pVTZ).^41,42^ All quantum-chemical calculations were performed with the Gaussian 16 program.^43^ The single-point energy of each species was calculated with the coupled cluster single–double and perturbative triple, CCSD(T), method^44^ at the B3LYP/aug-cc-pVTZ-optimized geometries to construct the reaction pathway scheme (RPS); all energies were corrected for ZPVE calculated with the B3LYP/aug-cc-pVTZ method.

## 3 Results

### 3.1 Quantum-Chemical Calculations

**Figure 1:**
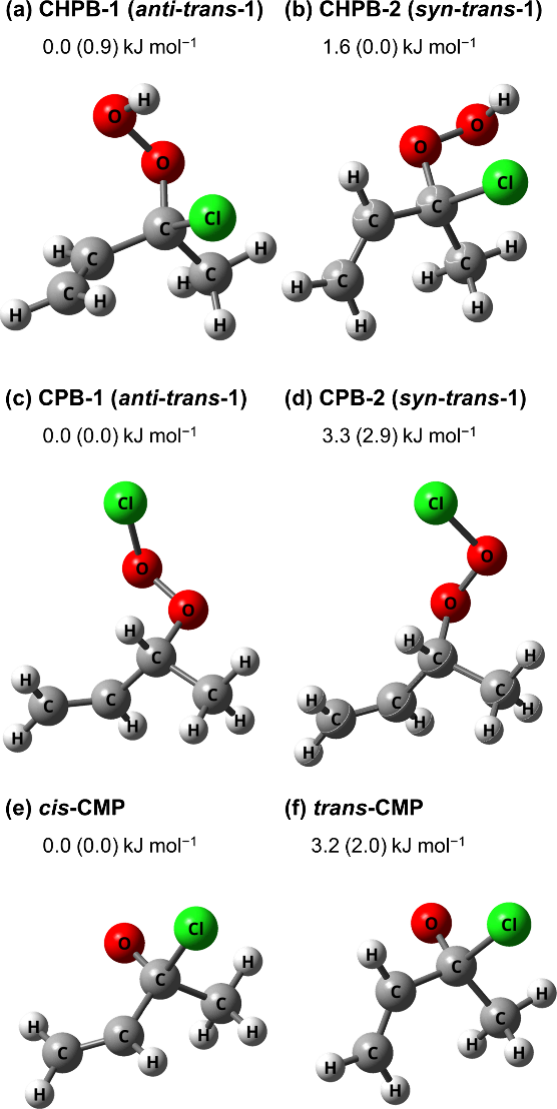
Geometries of representative conformers of (C_2_H_3_)CCl(CH_3_)OOH (CHPB), (C_2_H_3_)CH(CH_3_)OOCl (CPB), and (C_2_H_3_)CCl(CH_3_)O (CMP) calculated with the B3LYP/aug-cc-pVTZ method. (a) CHPB-1, (b) CHPB-2, (c) CPB-1, (d) CPB-2, (e) *cis*-CMP, and (f) *trans*-CMP. Relative energies were calculated with the CCSD(T)/aug-cc-pVTZ//B3LYP/aug-cc-pVTZ method, and those in parentheses were calculated with the B3LYP/aug-cc-pVTZ method.

The major path of MVKO + HCl is the formation of the H-transferred adduct 2-chloro-2-hydroperoxybut-3-ene [(C_2_H_3_)CCl(CH_3_)OOH, CHPB].
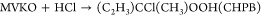
8The structures of the two most stable conformers of the CHPB, optimized with the B3LYP/aug-cc-pVTZ method, are presented in Figure 1a,b. The Cartesian coordinates of all 16 possible conformers of CHPB are listed in Table S1; their structures and relative energies are shown in Figure S1. More detailed structural parameters of the six lowest-energy conformers of CHPB are depicted in Figure S2. The energy of CHPB-1 is smaller than that of CHPB-2 by 1.6 kJ mol^–1^ according to the CCSD(T)/aug-cc-pVTZ//B3LYP/aug-cc-pVTZ method, but CHPB-1 is higher in energy than CHPB-2 by 0.9 kJ mol^–1^ according to the B3LYP/aug-cc-pVTZ method (listed in parentheses). We located these conformers by relaxed scans on the dihedral angles Φ(CCCO), Φ(CCOO), and Φ(COOH) at 10° intervals with the B3LYP/aug-cc-pVTZ method; representative results are shown in Figures S3 and S4. The interconversion among the six lowest-energy conformers of CHPB with energy calculated with the CCSD(T)/aug-cc-pVTZ method is summarized in Figure S5. The barriers for the interconversions among *anti*-conformers CHPB-1, CHPB-3, and CHPB-4 (except CHPB-1 to CHPB-3) and those among *syn*-conformers CHPB-2, CHPB-5, and CHPB-6 are less than 8 kJ mol^–1^; the barrier for the interconversion between CHPB-1 and CHPB-2 is ∼17 kJ mol^–1^.

**Figure 2:**
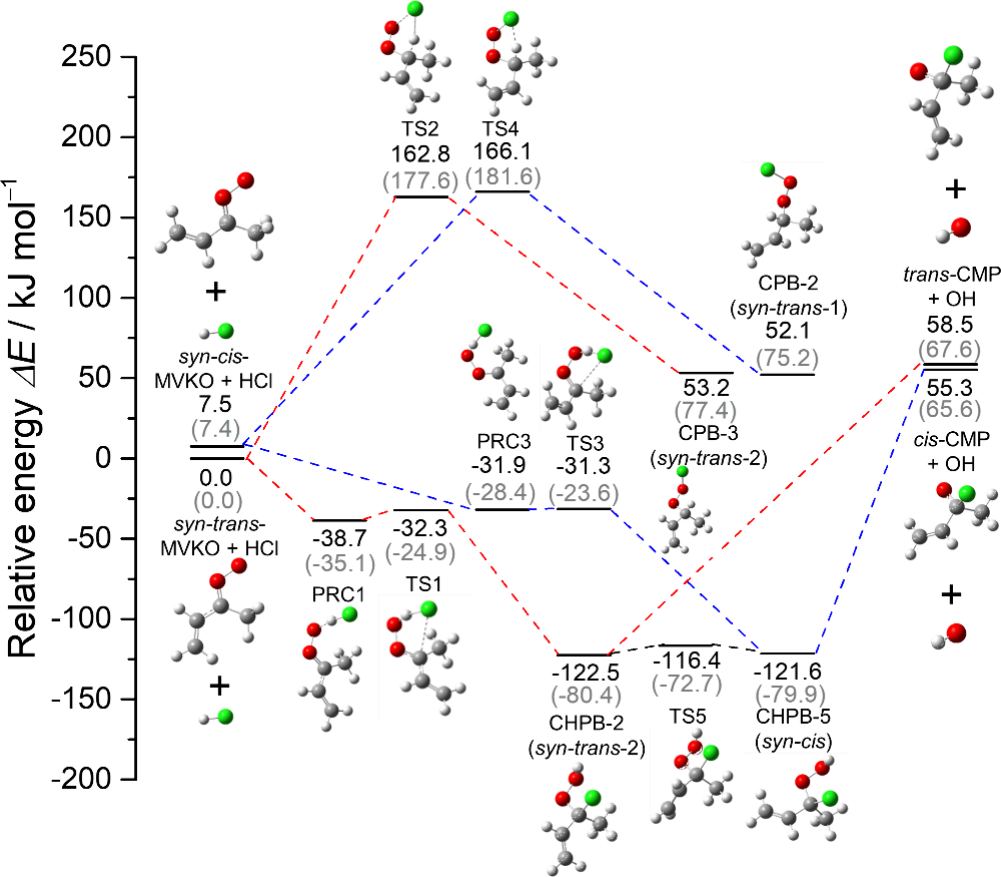
Reaction pathway scheme (RPS) of *syn*-MVKO + HCl predicted with the CCSD(T)/aug-cc-pVTZ//B3LYP/aug-cc-pVTZ method. The dashed red and dashed blue lines indicate the reactions of HCl with *syn–trans*-MVKO and *syn–cis*-MVKO, respectively. Energies calculated with the B3LYP/aug-cc-pVTZ method are listed in parentheses for comparison. Energies in kJ mol^–1^ were corrected for zero-point vibrational energies calculated with the B3LYP/aug-cc-pVTZ method. CHPB indicates (C_2_H_3_)CCl(CH_3_)OOH, CPB indicates (C_2_H_3_)CH(CH_3_)OOCl, CMP indicates the (C_2_H_3_)CCl(CH_3_)O radical, and PRC indicates a prereaction complex.

Figure 2 depicts the reaction pathway scheme (RPS) of the reaction *syn-*MVKO + HCl predicted with CCSD(T)/aug-cc-pVTZ//B3LYP/aug-cc-pVTZ method; energies calculated with the B3LYP/aug-cc-pVTZ method are listed in parentheses for comparison. The RPS of *anti-*MVKO + HCl is shown in Figure S6. The dashed red and dashed blue lines in Figure 2 indicate the reactions of HCl with *syn–trans*-MVKO and *syn–cis*-MVKO, respectively. Prereaction complexes, PRC1 and PRC3, were located; their structures are presented in Figure S7a and b, respectively, and associated Cartesian coordinates are listed in Table S2. The exothermicity of *syn–trans*-MVKO + HCl → CHPB-2 is ∼123 kJ mol^–1^, whereas that of *syn–cis*-MVKO + HCl → CHPB-5 is ∼129 kJ mol^–1^; only direct formation channels and the interconversion between CHPB-2 and CHPB-5 are presented. For *anti*-MVKO + HCl, CHPB-1, and CHPB-4 were produced directly (Figure S6). These conformers of CHPB might further dissociate to OH + (C_2_H_3_)CCl(CH_3_)O (1-chloro-1-methyl-2-propenyloxy, CMP), which have energies of 55–59 kJ mol^–1^ above those of *syn*–*trans*-MVKO + HCl.
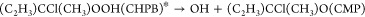
9

Two conformers of CMP (*cis*- and *trans*-) were predicted to be stable; their structures are shown in Figure S7c,d, and the associated Cartesian coordinates are listed in Table S2. In the reactions of the smaller Criegee intermediate *syn*-CH_3_CHOO + HCl, the H-transferred adduct *syn*-CEHP [CH_3_CHClOOH] decomposes to either CH_3_CHClO + OH or CH_3_C(O)Cl + H_2_O; the latter via the hydrogen transfer to the terminal OH before decomposition. In contrast, CHPB [(C_2_H_3_)CCl(CH_3_)OOH] has no feasible H atom to transfer to OH, so that the corresponding channel for the formation of H_2_O does not take place.

A second reaction pathway for MVKO + HCl is the formation of a second adduct, (C_2_H_3_)CH(CH_3_)OOCl (3-chloroperoxybut-1-ene, CPB), with the Cl atom attached to the terminal O atom and the H atom of HCl transferred to the C atom connecting the O–O moiety.
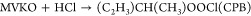
10

Four conformers of CPB were predicted to be stable, as shown in Figure S8; associated Cartesian coordinates are listed in Table S3. The *syn*–*trans*-CPB conformers (CPB-2 and CPB-3) are greater in energy by 3–4 kJ mol^–1^ than that of *anti*–*trans*-CPB (CPB-1) produced in *anti*-MVKO + HCl (Figure S6). Because of the large barrier (>150 kJ mol^–1^) and the endothermicity (38–53 kJ mol^–1^) involved, these channels are unlikely to occur in MVKO + HCl under our experimental conditions. The geometries of all transition states are depicted in Figure S9; associated Cartesian coordinates are listed in Table S4.

The harmonic and anharmonic vibrational wavenumbers and harmonic IR intensities of the precursor (*Z*)-(CH_2_I)HC=C(CH_3_)I are compared with experimental results in Table S5. We fitted the experimental vibrational wavenumbers against calculated harmonic vibrational wavenumbers of (*Z*)-(CH_2_I)HC=C(CH_3_)I for a spectral region 800–3300 cm^–1^ in Figure S10a and derived a scaling equation *y* = (0.955 ± 0.003)*x* + (30.7 ± 6.7), in which *y* is the scaled vibrational wavenumber and *x* is the calculated harmonic vibrational wavenumber; this equation was used for wavenumbers greater than 2000 cm^–1^. To further improve the scaling for the region below 2000 cm^–1^, we fitted the data for a spectral region 800–2000 cm^–1^ in Figure S10b and derived a scaling equation *y* = (0.971 ± 0.016)*x* + (10.4 ± 20.7); this equation was used for wavenumbers smaller than 2000 cm^–1^. As listed in Table S5, the average absolute deviation for scaled harmonic vibrational wavenumbers from experiments is (7.8 ± 4.9) cm^–1^, whereas that for anharmonic vibrational wavenumbers is (11.1 ± 6.2) cm^–1^. These scaling equations were employed to all species considered in this work; although the scaling should be applied only for wavenumbers above 800 cm^–1^, we applied it to vibrational modes below 800 cm^–1^ and listed them in tables for simplicity. The scaled harmonic and anharmonic vibrational wavenumbers and harmonic IR intensities of six lowest-energy conformers of CHPB are listed in Table S6; those of four conformers of CPB and two conformers of CMP are listed in Tables S7 and S8, respectively.

The rotational constants of the ground and the vibrationally excited (*v* = 1) states and the ratios of *a*-, *b*-, and *c*-types of each infrared absorption mode of six lowest-energy conformers of CHPB, four conformers of CPB, and two conformers of CMP are listed in Tables S9–S11, respectively. The displacement vectors, rotational axes, and directions of dipole derivatives for modes *v*_9_–*v*_23_ of CHPB-1 and CHPB-2 are shown in Figures S11 and S12, respectively. The *a*-, *b*-, and *c*-type spectra and the resultant spectra for vibrational modes ν_9_–ν_23_ of CHPB-1 and CHPB-2, simulated with the PGOPHER program^45^ according to calculated rotational constants of the ground and the vibrationally excited (*v* = 1) states, ratios of *a*-, *b*-, and *c*-types of vibrational bands (Table S9), *J*_max_ = 150, *T* = 298 K, and Gaussian width (fwhm) = 1.28 cm^–1^, are depicted in Figures S13 and S14, respectively.

### 3.2 Infrared Spectra upon Irradiation of (*Z*)-(CH_2_I)HC=C(CH_3_)I/HCl/O_2_

The transient IR absorption spectra (820–1440 cm^–1^, instrumental resolution 1.0 cm^–1^) of a flowing mixture of (*Z*)-(CH_2_I)HC=C(CH_3_)I/HCl/O_2_ (0.03/0.03/85.0, *P*_T_ = 85.1 Torr) at 298 K were recorded with an external ADC (time intervals 4 ns), as depicted in Figure S15. The spectrum before photolysis is shown in Figure S15a and that of MVKO (reported in ref (18)) is shown in Figure S15b for reference; difference spectra recorded 0–5, 5–10, and 30–35 μs upon irradiation of the mixture at 248 nm are presented in Figure S15c–e, respectively; positive features represent production, whereas negative ones representing the destruction of the precursor upon photolysis are truncated. We processed the spectra in Figures S15c–e by adding back the scaled spectrum of the precursor, Figure S15a, and subtracting the scaled spectrum of MVKO, Figure S15b, to yield spectra in Figures S15f–h; the region that might be interfered with by the intense absorption of (*Z*)-(CH_2_I)HCC(CH_3_)I is shaded gray. Because of the unsatisfactory S/N, only bands near 931, 1065, 1118, and 1360 cm^–1^ could be identified as absorption of possible product.

**Figure 3:**
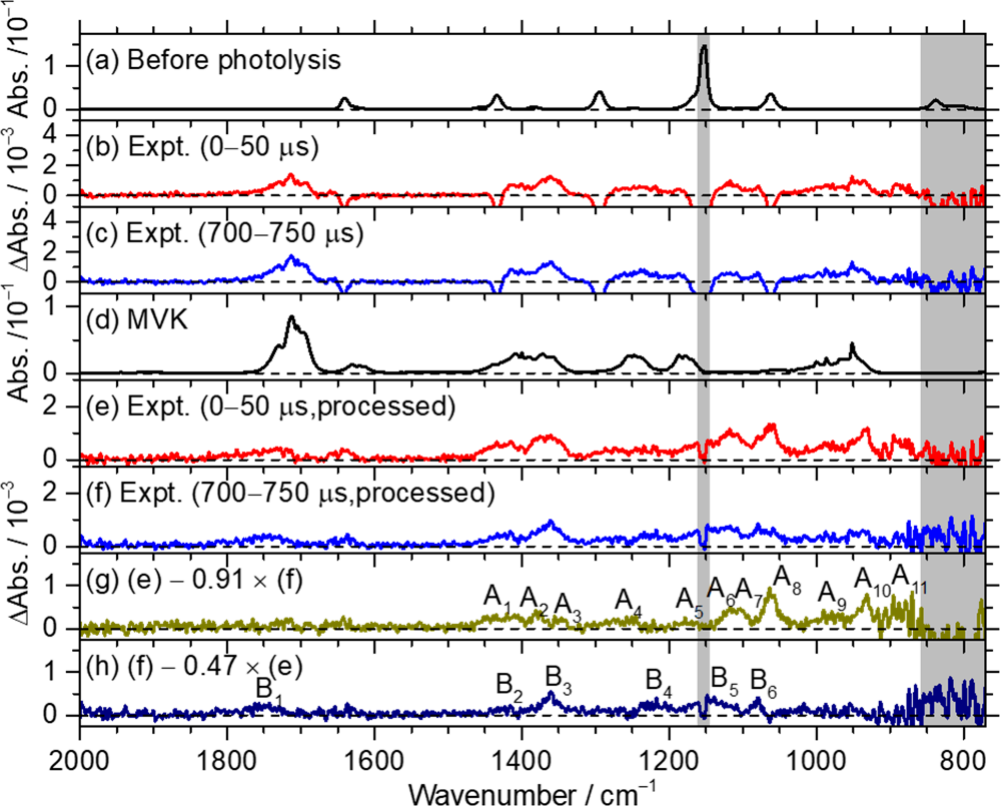
IR spectra recorded with an internal ADC upon photolysis at 248 nm of a flowing mixture of (*Z*)-(CH_2_I)HC=C(CH_3_)I/HCl/O_2_ (0.03/0.03/83.0, *P*_T_ = 83.1 Torr) at 298 K. (a) Absorption spectrum before photolysis. Difference spectra recorded 0–50 μs (b) and 700–750 μs (c) after irradiation; negative bands of precursor are truncated. (d) IR absorption spectrum of methyl vinyl ketone (MVK). (e, f): Processed spectra of (b, c) with absorption bands of the precursor (*Z*)-(CH_2_I)HCC(CH_3_)I, spectrum (a), added back and those of MVK subtracted. (g) Difference spectrum from spectrum in (e) minus 0.91 times that in (f). (h) Difference spectrum from spectrum in (f) minus 0.47 times that in (e). The region interfered with by absorption of the precursor molecule (1140–1160 cm^–1^) and the region with poor S/N (770–860 cm^–1^) due to cutoff of the filter are shaded gray. New features in groups A and B are marked A_1_–A_11_ in (g) and B_1_–B_6_ in (h), respectively. Instrumental resolution is 2.0 cm^–1^.

An internal ADC with temporal resolution 12.5 μs to cover an extended reaction period with improved S/N was also used. The spectrum in region 800–2000 cm^–1^ (resolution 2 cm^–1^) of a flowing mixture of (*Z*)-(CH_2_I)HC=C(CH_3_)I/HCl/O_2_ (0.03/0.03/83.0, *P*_t_ = 83.1 Torr) at 298 K are presented in Figure 3a; two spectra obtained under the same conditions were averaged. The difference spectra recorded 0–50 and 700–750 μs after irradiation at 248 nm are shown in Figure 3b and c, respectively. A spectrum of the end-product methyl vinyl ketone (MVK) is depicted in Figure 3d for comparison. We processed these spectra by adding back bands of (*Z*)-(CH_2_I)HC=C(CH_3_)I and subtracting bands of MVK; the resultant spectra are presented in Figure 3e and f, respectively. On comparison of Figure 3e and f, it is clear that some bands decreased faster than the others. We further subtract 0.91 times Figure 3f from Figure 3e to yield Figure 3g, which presents better the spectrum of a species that decayed slightly faster; these bands are marked as A_1_–A_11_ and termed group A. In general, the spectrum of group A agree with that obtained with the external ADC (Figure S15g), except that the latter has poor S/N. The observed wavenumbers and relative IR intensities of bands in group A are listed in Table 1. Figure 3h, obtained on subtracting 0.47 times Figure 3e from Figure 3f, represents the spectrum of a species that survived in a later period; these bands are marked as B_1_–B_6_ and termed group B. The observed wavenumbers and relative IR intensities of bands in group B are listed in Table S12.

**Table 1:** Comparison of Observed Wavenumbers (cm^–1^) and Relative Intensities of Bands in Group A with the Scaled Harmonic and Anharmonic Vibrational Wavenumbers and Infrared Intensities of Isomers of (C_2_H_3_)CCl(CH_3_)OOH, CHPB-1, and CHPB-2, Predicted with the B3LYP/aug-cc-pVTZ Method

			CHPB-1 (*anti*–*trans*-1)			CHPB-2 (*syn*–*trans*-1)		
mode	experiment		scaled harmonic		anharm.	scaled harmonic		anharm.
*v*_8_			1674^a^	(4)^b^	1676	1665	(0)	1662
*v*_9_			1455	(2)	1447	1461	(4)	1459
*v*_10_			1448	(2)	1441	1457	(3)	1449
*v*_11_	1415	(76)^c^	1416	(19)	1415	1421	(17)	1416
*v*_12_	1381	(41)	1377	(7)	1383	1381	(17)	1380
*v*_13_	1350	(24)	1370	(62)	1359	1370	(60)	1358
*v*_14_			1297	(2)	1303	1305	(1)	1313
*v*_15_	1249	(24)				1254	(12)	1250
	1178	(18)	1202	(38)	1196			
*v*_16_	1118	(45)				1146	(50)	1141
	1103	(52)	1125	(73)	1118			
*v*_17_	1065	(100)	1083	(36)	1073	1070	(70)	1070
*v*_18_			1026	(7)	1028	1012	(14)	1025
*v*_19_	978	(65)	999	(8)	997	1003	(6)	997
*v*_20_	931	(76)	964	(38)	962	962	(39)	958
*v*_21_			951	(6)	941	933	(2)	926
*v*_22_	895	(59)	903	(12)	900	895	(33)	891
*v*_23_			805	(62)	802	811	(78)	810

a Scaling equation *y* = 0.971*x* + 30.7, in which *y* is the scaled harmonic vibrational wavenumber and *x* is the harmonic vibrational wavenumber calculated with the B3LYP/aug-cc-pVTZ method.

b Harmonic IR intensities (in km mol^–1^) are listed in parentheses.

c Percentage integrated intensities relative to that of the most intense band near 1065 cm^–1^ (ν_17_).

## 4 Discussion

**Figure 4:**
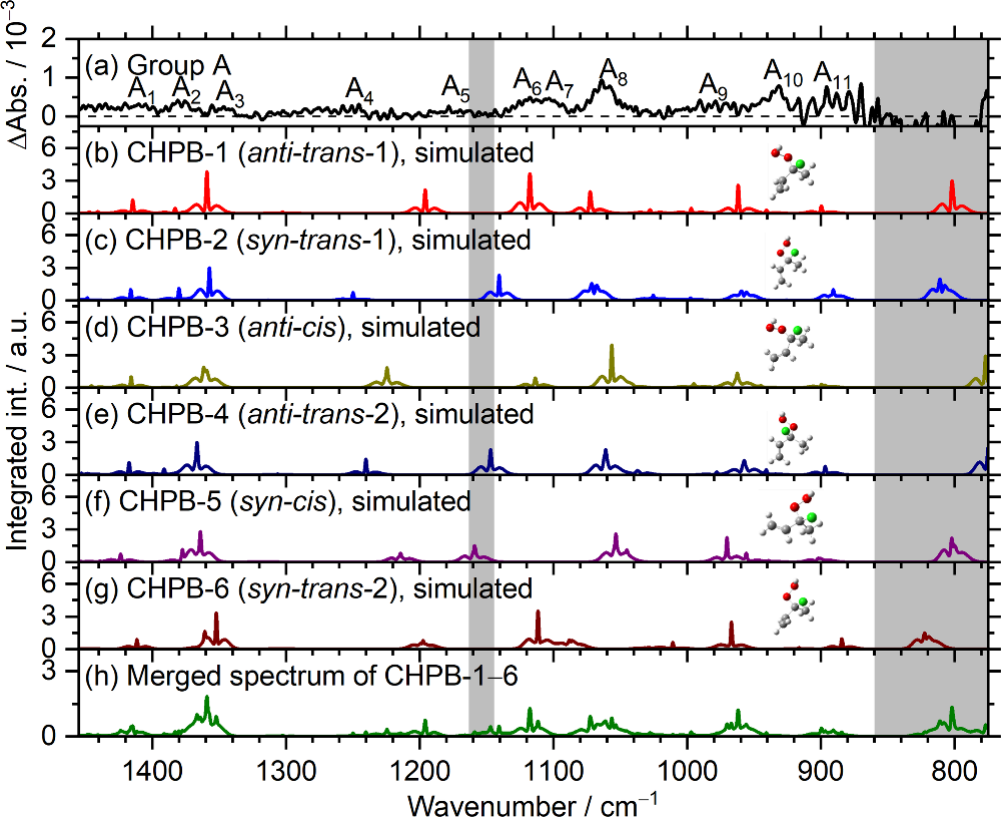
Comparison of bands in group A with simulated spectra of six lowest-energy conformers of CHPB, (C_2_H_3_)CCl(CH_3_)OOH. (a) Absorption spectrum of group A taken from Figure 3g. IR spectra of fundamental bands of CHPB-1 (b), CHPB-2 (c), CHPB-3 (d), CHPB-4 (e), CHPB-5 (f), and CHPB-6 (g) simulated according to rotational parameters, anharmonic vibrational wavenumbers, and IR intensities predicted with the B3LYP/aug-cc-pVTZ method; Gaussian fwhm is 1.28 cm^–1^. (h) Merged spectrum of CHPB-1 to CHPB-6 according to Boltzmann distribution. The regions interfered with by absorption of the parent molecule (1140–1160 cm^–1^) and the cutting-off of the filter (770–860 cm^–1^) are shaded gray.

Spectra of bands in group A in Figure 3g are reproduced in Figure 4a. The 11 features A_1_–A_11_ are compared with predicted IR spectra of fundamental bands of six lowest-energy conformers of CHPB according to rotational-contour simulations with PGOPHER^45^ and anharmonic vibrational wavenumbers, as presented in Figure 4b–g; details of the simulated spectra for CHPB-1 and CHPB-2 are presented in Figures S13 and S14, respectively. The spectrum of group A is also compared with IR stick spectra of six lowest-energy conformers of CHPB according to anharmonic vibrational wavenumbers predicted with the B3LYP/aug-cc-pVTZ method in Figure S16; predicted overtone and combination bands are presented in light colors. The predicted contributions of overtone and combination bands are insignificant. The spectrum of group A is also compared with IR stick spectra of four conformers of CPB and two conformers of CMP according to scaled harmonic vibrational wavenumbers predicted with the B3LYP/aug-cc-pVTZ method in Figures S17 and S18, respectively.

On comparison of spectral patterns (band positions and relative IR intensities), we found that the observed spectrum in group A agrees much better with CHPB than with CPB or CMP. Similar to other species we studied,^27,31^ the simulated spectra show narrower contours than the observed ones, with the Q-branches more prominent than observations. This effect might be partly due to the product being highly internally excited because of the exothermicity; the unaccounted contribution from the ^37^Cl-isotopic species, which is expected to shift slightly, might also cause the broadening.

To assign the observed bands in group A to a specific conformer of CHPB is challenging because the spectra of these conformers are similar. Assuming that the interconversion among these conformers is feasible, as predicted from calculations, we apply a Boltzmann distribution for these conformers according to their CCSD(T) energies and derive the simulated spectrum for a combination of six conformers of CHPB, as shown in Figure 4h; the Boltzmann distribution of conformers are 32:17:16:13:12:11 for CHPB-1 to CHPB-6, respectively. The resultant predicted spectral pattern agrees better with experiments; the number of bands is greater than that of a single conformer. The major contribution comes from CHPB-1, CHPB-2, and CHPB-3, which have energies within 1.7 kJ mol^–1^.

The wavenumbers and relative intensities of experimentally observed bands in group A are compared with the scaled harmonic and anharmonic vibrational wavenumbers and harmonic infrared intensities of vibrational modes ν_8_–ν_23_ of CHPB-1 and CHPB-2 in Table 1. The observed features A_1_–A_3_ at 1415, 1381, and 1350 cm^–1^ agree satisfactorily with the anharmonic vibrational wavenumbers predicted at 1415 (ν_11_, CH_2_ scissor), 1383 (ν_12_, CH_3_ umbrella), and 1359 (ν_13_, HOO bend) cm^–1^ of CHPB-1, and similar modes of CHPB-2 at 1416, 1380, and 1358 cm^–1^; corresponding scaled harmonic vibrational wavenumbers of CHPB-1 and CHPB-2 were predicted at 1416, 1377, and 1370 cm^–1^, and 1421, 1381, and 1370 cm^–1^, respectively (Table 1). The weak bands A_4_ and A_5_ at 1249 and 1178 cm^–1^ might correspond to the C–C–C_H3_ antisymmetric stretching mode (ν_15_) of CHPB-2, predicted near 1250 (1254) cm^–1^, and the ν_15_ of CHPB-1, predicted near 1196 (1202) cm^–1^, respectively; listed numbers are anharmonic vibrational wavenumbers, those in parentheses are scaled harmonic vibrational wavenumbers, and the subscript indicate the position of the carbon atom. The overlapped bands A_6_ and A_7_ near 1118 and 1107 cm^–1^ might correspond to ν_16_ (C–C–C_H3_ bend mixed with C–O stretch) of CHPB-2, predicted near 1141 (1146 cm^–1^), and ν_16_ (C–O stretch mixed with CH_3_ wag) of CHPB-1, predicted near 1118 (1125) cm^–1^, respectively. The four bands in this region corresponding to only two modes clearly indicated that at least two conformers of CHPB are present in our experiment.

The most intense band A_8_ at 1065 cm^–1^ corresponds to ν_17_ (C–C_H3_ stretch) of CHPB-1, predicted near 1073 (1083) cm^–1^, and ν_17_ (C–C_H3_ stretch mixed with CH_3_ wag) of CHPB-2, predicted near 1070 (1070) cm^–1^. A weaker A_9_ band at 978 cm^–1^ might correspond to the CH out-of-plane bending mode of C_2_H_3_ (ν_19_) of CHPB-1, predicted near 997 (999) cm^–1^, and the ν_19_ of CHPB-2, predicted near 997 (1003) cm^–1^. Another intense band A_10_ at 931 cm^–1^ corresponds to the CH_2_ wagging mode (ν_20_) of CHPB-1, predicted near 962 (964) cm^–1^, and the ν_20_ of CHPB-2, predicted near 958 (962) cm^–1^. The S/N for band A_11_ near 895 cm^–1^ is poor; it might be tentatively assigned to ν_22_ (O–O stretch) of CHPB-1, predicted near 900 (903 cm^–1^), and ν_22_ (O–O stretch mixed with the C–C_CH3_ stretch) of CHPB-2, predicted near 891 (894) cm^–1^. All vibrational modes of CHPB-1 and CHPB-2 in region 850–1700 cm^–1^ with IR intensity >8 km mol^–1^ have been observed except the ν_18_ mode of CHPB-2 predicted near 1025 (1012) cm^–1^ with IR intensity 14 km mol^–1^, which might be overlapped with band A_8_ near1065 cm^–1^.

From the above discussion, the observed bands in group A are best assigned to CHPB, with contributions from at least conformers CHPB-1 and CHPB-2. The average absolute deviations between experimental wavenumbers and predicted anharmonic (scaled harmonic) vibrational wavenumbers are 12 ± 10 (16 ± 12) cm^–1^ for CHPB-1 and 9 ± 11 (13 ± 13) cm^–1^ for CHPB-2.

Spectra of bands in group B are reproduced in Figure S19a. The six features B_1_–B_6_ are compared with IR stick spectra of two conformers of CMP, a representative conformer of CPB, CPB-1, and CH_3_CHClC(O)CH_3_,^46^ as presented in Figure S19b–e. On comparison of spectral patterns (band positions and relative IR intensities), we tentatively assigned most observed bands in group B to CMP, a product after the dissociation of OH from the adduct CHPB, as discussed in Supporting Information, Note SA. Bands B_2_, B_3_, B_5_, and B_6_ observed at 1423, 1360, 1144, and 1080 cm^–1^ correspond satisfactorily with the anharmonic vibrational wavenumbers predicted near 1421, 1401, 1127, and 1046 cm^–1^ for *cis*-CMP and 1433, 1394, 1148, 1059 cm^–1^ for *trans*-CMP. However, weak bands B_1_ and B_4_, observed at 1750 and 1220 cm^–1^, respectively, have no corresponding modes predicted for CMP; they might be associated with another species.

In our previous experiments of CH_2_OO + HCl,^27^ we did not observe the corresponding dissociation products OH + CH_2_ClO, reaction 2a, but observed products HC(O)Cl + H_2_O, reaction 2b, which were produced from secondary reactions OH + HCl → H_2_O + Cl, reaction 4, and CH_2_ClO + O_2_ → HC(O)Cl + HO_2_, reaction 3, because O_2_ and HCl were two major reactants in the system. Similarly, in the experiments of CH_3_CHOO + HCl,^31^ we observed CH_3_C(O)Cl + H_2_O, reaction 6b, some of which were produced from secondary reactions CH_3_CHClO + O_2_, reaction 7, and OH + HCl → H_2_O + Cl. One would expect that similar secondary reactions to occur for CMP (C_2_H_3_)CCl(CH_3_)O, the OH-decomposition product of CHPB, reaction 9. However, CMP has no feasible H atom to be abstracted by O_2_, so that no similar secondary product could be produced. We did observe the formation of H_2_O, likely from OH + HCl → H_2_O + Cl. The most likely reaction for CMP would be the C–Cl fission to produce methyl-vinyl ketone, (C_2_H_3_)C(O)(CH_3_), which we did observe (Figure 3); MVK was also produced during the production of MVKO via the self-reaction of MVKO.

## 5 Conclusions

Using a step-scan Fourier-transform spectrometer, we detected the hydrogen-transferred adduct (C_2_H_3_)CCl(CH_3_)OOH (2-chloro-2-hydroperoxybut-3-ene, CHPB) and, tentatively, its decomposition products (C_2_H_3_)CCl(CH_3_)O (1-chloro-1-methyl-2-propenyloxy, CMP) upon photolysis of a flowing mixture of (*Z*)-(CH_2_I)HC=C(CH_3_)I/HCl/O_2_ at 248 nm to initiate the reaction MVKO + HCl. The observed 11 bands in group A are assigned to the H-transferred adduct CHPB, similar to the adducts CH_2_ClOOH and CH_3_CHClOOH observed in CH_2_OO + HCl and CH_3_CHOO + HCl, respectively. No definitive assignments of the conformer could be derived, but most likely the lowest-energy conformers CHPB-1 and CHPB-2 have major contributions.

The observed four bands (B_2_, B_3_, B_5_, and B_6_) in group B are tentatively assigned to *trans*- and *cis*-CMP, (C_2_H_3_)CCl(CH_3_)O, produced from the OH-decomposition of CHPB, (C_2_H_3_)CCl(CH_3_)OOH. In similar reactions of CH_2_OO + HCl → CH_2_ClO + OH and CH_3_CHOO + HCl → CH_3_CHClO + OH, the products CH_2_ClO and CH_3_CHClO readily react with O_2_ in the system to form HC(O)Cl + HO_2_ and CH_3_C(O)Cl + HO_2_, respectively, so that neither CH_2_ClO nor CH_3_CHClO was observed. However, in the case of MVKO + HCl, the H-abstraction of the OH-dissociation product CMP by O_2_ was not observed because there is no feasible H atom in CMP.

## Supporting Information Available

Tentative assignments of bands in Group B (Note A); Cartesian coordinates of optimized conformers of CHPB, PRC, CMP, CPB, and all transition structures; vibrational wavenumbers and IR intensities of conformers of CHPB, CPB, and CMP; rotational parameters and ratios of *a*-/*b*-/*c*-type ratios for each vibrational state of conformers of CHPB, CPB, and CMP; comparison of observed wavenumbers and intensities of bands in Group B with those predicted for *trans*-CMP and *cis*-CMP; scaled harmonic vibrational wavenumbers of *s*-*trans*- and *s*-*cis*-C_2_H_3_C(O)Cl and six conformers of C_2_H_3_C(OH)Cl(CH_3_) (total 14 Tables); geometries of conformers of CHPB, PRC, CMP, CPB, and all transition structures; potential-energy for the conformation interconversion started from CHPB-1 and CHPB-2; potential energies for the interconversion among six lowest-energy conformers of CHPB; reaction pathway scheme of *anti*-MVKO + HCl; comparison of experimentally observed wavenumbers (cm^–1^) with the harmonic vibrational wavenumbers of (*Z*)-(CH_2_I)HCC(CH_3_)I; displacement vectors, directions of dipole derivatives, and rotational contours simulated for modes *v*_9_–*v*_23_ of CHPB-1 and CHPB-2; IR spectra recorded with an external ADC upon photolysis at 248 nm of a flowing mixture of (*Z*)-(CH_2_I)HC=C(CH_3_)I/HCl/O_2_; comparison of bands in group A with anharmonic vibrational stick spectra of conformers of CHPB, CPB, and CMP; comparison of bands in group B with IR stick spectra and absorption spectra of possible species (total 20 figures) (PDF)
