# Supplementary material for: Infrared Spectrum of the Adduct 2-Chloro-2-hydroperoxybut-3-ene [(C2H3)CCl(CH3)OOH] of the Reaction between the Criegee Intermediate Methyl Vinyl Ketone Oxide [C2H3C(CH3)OO] and HCl
Source: J Phys Chem A. 2024 Sep 26;128(40):8690–8. doi: 10.1021/acs.jpca.4c04936 (PMC11472334; doi:10.1021/acs.jpca.4c04936)
Supplement: Supplementary file 1 — jp4c04936_si_001.pdf [file jp4c04936_si_001.pdf]

Supporting Information

Infrared Spectrum of the Adduct 2-Chloro-2-hydroperoxybut-3-ene [$(C_2H_3)CCl(CH_3)OOH$] of the Reaction between the Criegee Intermediate Methyl Vinyl Ketone Oxide [$C_2H_3C(CH_3)OO$] and HCl

Hao Wang[a] and Yuan-Pern Lee[a,b*]

[a] *Department of Applied Chemistry and Institute of Molecular Science, National Yang Ming Chiao Tung University, Hsinchu 300093, Taiwan.*

[b] *Center for Emergent Functional Matter Science, National Yang Ming Chiao Tung University, Hsinchu 300093, Taiwan.*

**E-mail: yplee@nycu.edu.tw* (Y.-P. Lee)

Table of Contents

Note SA. Tentative assignments of bands in Group B-- S4

Table S1. Cartesian coordinates of optimized geometries of 16 conformers of CHPB [$(C_2H_3)C(CH_3)(Cl)OOH$] predicted with the B3LYP/aug-cc-pVTZ method--------------- S6

Table S2. Cartesian coordinates of optimized geometries of *syn-trans*-MVKO···HCl (PRC1), *syn-cis*-MVKO···HCl (PRC3) and two conformers (*trans*- and *cis*-) of CMP [$(C_2H_3)CCl(CH_3)O$] predicted with the B3LYP/aug-cc-pVTZ method -------------------S10

Table S3. Cartesian coordinates of optimized geometries of four conformers of CPB [$(C_2H_3)CH(CH_3)OOCl$] predicted with the B3LYP/aug-cc-pVTZ method----------------S11

Table S4. Cartesian coordinates of optimized geometries of TS1−TS8 predicted with the B3LYP/aug-cc-pVTZ method---S12

Table S5. Comparison of experimentally observed wavenumbers (cm^{-1}) and intensities of (*Z*)-$(CH_2I)HCC(CH_3)I$ with harmonic and anharmonic vibrational wavenumbers and infrared intensities predicted with the B3LYP/aug-cc-pVTZ-pp method------------------S14

Table S6. Vibrational wavenumbers and IR intensities of six lowest-energy conformers of CHPB predicted with the B3LYP/aug-cc-pVTZ method------------------------------------S15

Table S7. Vibrational wavenumbers and IR intensities of four conformers of CPB predicted with the B3LYP/aug-cc-pVTZ method --S18

Table S8. Vibrational wavenumbers and IR intensities of two conformers of CMP $(C_2H_3)CCl(CH_3)O$ predicted with the B3LYP/aug-cc-pVTZ method ---------------------S20

Table S9. Rotational parameters and *a*-/*b*-/*c*-type ratios for each vibrational state of six lowest-energy conformers of CHPB predicted with the B3LYP/aug-cc-pVTZ method - S21

Table S10. Rotational parameters and *a*-/*b*-/*c*-type ratios for each vibrational state of four conformers of CPB predicted with the B3LYP/aug-cc-pVTZ method ---------------------S24

Table S11. Rotational parameters and *a*-/*b*-/*c*-type ratios for each vibrational state of two conformers of CMP predicted with the B3LYP/aug-cc-pVTZ method --------------------S26

Table S12. Comparison of observed wavenumbers (cm^{-1}) and relative intensities of bands in Group B with scaled harmonic and anharmonic vibrational wavenumbers and infrared intensities of two conformers of $(C_2H_3)CCl(CH_3)O$, *trans*-CMP and *cis*-CMP, predicted with the B3LYP/aug-cc-pVTZ method --S27

Table S13. Comparison of experimental vibrational wavenumbers (cm^{-1}) and relative IR intensities of *s-trans*-and *s-cis*-$C_2H_3C(O)Cl$ with scaled harmonic vibrational wavenumbers and relative IR intensities predicted with the B3LYP/aug-cc-pVTZ method--------------S28

Table S14. Scaled harmonic vibrational wavenumbers and IR intensities of six conformers of $C_2H_3C(OH)Cl(CH_3)$ predicted with the B3LYP/aug-cc-pVTZ method-----------------S29

Figure S1. Geometries of sixteen conformers of $(C_2H_3)C(CH_3)(Cl)OOH$ (CHPB) calculated with the B3LYP/aug-cc-pVTZ method --S31

Figure S2. Geometries of six lowest-energy conformers of $(C_2H_3)C(CH_3)(Cl)OOH$ (CHPB) calculated with the B3LYP/aug-cc-pVTZ method --S32
Figure S3. Potential energies for the conformation interconversion started from *anti-trans*1-CHPB (CHPB-1) calculated with the B3LYP/aug-cc-pVTZ method----------------------S33
Figure S4. Potential energies for the conformation interconversion started from *syn-trans*1-CHPB (CHPB-2) calculated with the B3LYP/aug-cc-pVTZ method----------------------S37
Figure S5. Potential energies for the interconversion among six lowest-energy conformers of CHPB calculated with the CCSD(T)/aug-cc-pVTZ//B3LYP/aug-cc-pVTZ method--S42
Figure S6. Reaction pathway scheme (RPS) of *anti*-MVKO + HCl predicted with the CCSD(T)/aug-cc-pVTZ//B3LYP/aug-cc-pVTZ method -----------------------------------S43
Figure S7. Geometries of two pre-reactive complexes and two conformers of $(C_2H_3)C(CH_3)(Cl)O$ (CMP) calculated with the B3LYP/aug-cc-pVTZ method ----------S44
Figure S8. Geometries of four conformers of $(C_2H_3)CH(CH_3)OOCl$ (CPB) calculated with the B3LYP/aug-cc-pVTZ method ---S45
Figure S9. Geometries of TS1−TS8 calculated with the B3LYP/aug-cc-pVTZ method ---S46
Figure S10. Comparison of experimentally observed wavenumbers (cm^{-1}) with the harmonic vibrational wavenumbers of (Z)-$(CH_2I)HCC(CH_3)I$ predicted with the B3LYP/aug-cc-pVTZ-pp method---S48
Figure S11. Displacement vectors (blue arrows) and directions of dipole derivatives (brown arrows) for modes v_9–v_{23} of CHPB-1 predicted with the B3LYP/aug-cc-pVTZ method S49
Figure S12. Displacement vectors (blue arrows) and directions of dipole derivatives (brown arrows) for modes v_9–v_{23} of CHPB-2 predicted with the B3LYP/aug-cc-pVTZ method ---S51
Figure S13. Rotational contours simulated for modes v_9–v_{23} of CHPB-1-----------------S53
Figure S14. Rotational contours simulated for modes v_9–v_{23} of CHPB-2-----------------S56
Figure S15. IR spectra recorded with an external ADC upon photolysis at 248 nm of a flowing mixture of (Z)-$(CH_2I)HC=C(CH_3)I/HCl/O_2$ (0.03/0.03/85.0, P_T = 85.1 Torr) at 298 K --S59
Figure S16. Comparison of bands in group A with anharmonic vibrational stick spectra of six conformers of CHPB --S60
Figure S17. Comparison of bands in group A with anharmonic vibrational stick spectra of four conformers of CPB--S61
Figure S18. Comparison of bands in group A with anharmonic vibrational stick spectra of two conformers of $(C_2H_3)C(CH_3)(Cl)O$ (CMP) --S62
Figure S19. Comparison of bands in group B with IR stick spectra and absorption spectra of CMP, CPB and $CH_3CHClC(O)CH_3$---S63

Figure S20. Comparison of bands in group B with IR stick spectra and absorption spectra of $(C_2H_3)C(O)Cl$ and $(C_2H_3)C(OH)Cl(CH_3)$ -- S64
References -- S65

Note SA. Tentative assignments of bands in Group B.

Spectra of bands in group B are reproduced in Figure S19a. The six features B_1−B_6 are compared with IR stick spectra of two conformers of CMP, a representative conformer of CPB, CPB-1, and $CH_3CHClC(O)CH_3$, [1] as presented in Figures S19b−S19e. On comparison of spectral patterns (band positions and relative IR intensities), we found that the observed spectrum in group B might have contributions from CMP. The band B_6 observed at 1080 cm^{-1} might be associated with the ν_{15} mode of *trans*-CMP near 1059 (1065) cm^{-1} or the ν_{14} mode of *cis*-CMP near 1046 (1052) cm^{-1}; listed numbers are anharmonic vibrational wavenumbers and those in parentheses are scaled harmonic vibrational wavenumbers. The band B_5 at 1144 cm^{-1} might be associated with the ν_{13} mode of *trans*-CMP predicted near 1148 (1184) cm^{-1} and of *cis*-CMP near 1127 (1162) cm^{-1}. The most intense band B_3 at 1360 cm^{-1} might be assigned to the ν_{10} mode predicted for *trans*-CMP near 1394 (1400) cm^{-1} and for *cis*-CMP near 1401 (1405) cm^{-1}. The weak B_2 band observed at 1423 cm^{-1} might be associated with the ν_9 mode of *trans*-CMP predicted near 1433 (1446) cm^{-1} and of *cis*-CMP near 1421 (1429) cm^{-1}. However, weak bands B_1 observed at 1750 cm^{-1} and B_4 at 1220 cm^{-1} have no corresponding modes predicted for CMP; they might be associated with another species. Modes ν_{18} and ν_{19} of *trans*-CMP predicted near 953 (955) and 921 (921) cm^{-1} and mode ν_{18} of *cis*-CMP predicted near 955 (962) cm^{-1} have IR intensities 40, 29, and 35 km mol^{-1}, respectively. They might correspond to some weak bands in region 940−1020 cm^{-1}, but the poor S/N prevents definitive assignments. A comparison of bands in group B to those predicted for *trans*- and *cis*-CMP is presented in Table S12. The average absolute deviations between experimental wavenumbers and predicted anharmonic (scaled harmonic) vibrational wavenumbers are 17 ± 13 (30 ± 13) cm^{-1} for *trans*- CMP and 24 ± 18 (24 ± 17) cm^{-1} for *cis*-CMP.

The observed bands in group B agree poorly with those predicted for another possible product CPB; a representative spectrum of CPB-1 is depicted in Figure S19d for comparison. Although most reported bands of $CH_3CHClC(O)CH_3$, Figure S19e,[1] appeared to agree with those in group B; the observed band B_1 has a much smaller intensity than that reported for $CH_3CHClC(O)CH_3$. Considering that the formation of $CH_3CHClC(O)CH_3$ involves a transfer of Cl in CMP to the neighboring carbon atom followed by hydrogen addition to the terminal carbon atom, it is unlikely to occur. We also compared with additional potential products such as $(C_2H_3)C(O)Cl$ (CH_3-dissociation product of CMP) [2,3] and $(C_2H_3)C(OH)Cl(CH_3)$, the H-addition product of CMP, as presented in Figure S20; the vibrational wavenumbers are listed in Tables S13 and S14, respectively. The observed bands in group B agree poorly with the experimental results of $(C_2H_3)C(O)Cl$ and the calculated scaled harmonic vibrational wavenumbers of $(C_2H_3)C(OH)Cl(CH_3)$. We hence tentatively

assigned observed bands B_2, B_3, B_5 and B_6 in group B to CMP, a product via the dissociation of OH from the adduct CHPB.

According to the subtracting factors in Figure 3, in spectrum recorded 0−50 μs (Figure 3e) the intensity ratio of bands in group A to those in group B is 1.00 : 0.91 (or 0.52 : 0.48). Similarly, in the spectrum recorded 700−750 μs (Figure 3f), the ratio of group A to group B is 0.47 : 1:00 (or 0.32 : 0.68). Bands in Group B appeared to be present even during 0−50 μs, indicating that the likely carrier CMP was produced via decomposition of internally excited adduct CHPB, not through the unimolecular decomposition of thermalized CHPB. It seems to be unlikely that bands of the CMP radical persisted longer than those of CHPB. However, this might be because CMP has no feasible H atom to be abstracted by O_2 or other species, so that its decay is slower than expected.

Table S1. Cartesian coordinates of optimized geometries of 16 conformers of CHPB [(C_2H_3)C(CH_3)(Cl)OOH] predicted with the B3LYP/aug-cc-pVTZ method

atom	x	y	z	x	y	z
	CHPB-1 (*anti-trans*-1)			CHPB-2 (*syn-trans*-1)		
C1	-2.12426	-0.89548	-0.37718	-2.62143	-0.27138	-0.16715
C2	-1.00016	-0.98442	0.31351	-1.42312	-0.06470	-0.69340
C3	0.07898	0.05157	0.46330	-0.12433	-0.17184	0.04573
C4	0.01578	0.79108	1.79250	-0.17091	-0.63619	1.48309
H1	-2.35666	-0.03552	-0.98858	-2.76728	-0.53957	0.86962
H2	-2.84508	-1.70090	-0.35399	-3.51101	-0.16818	-0.77248
H3	-0.78868	-1.86955	0.90449	-1.31430	0.20477	-1.73599
H4	0.86075	1.46980	1.88538	-0.79350	0.02860	2.07560
H5	0.04569	0.06682	2.60648	-0.58139	-1.64597	1.51511
H6	-0.91141	1.35430	1.85070	0.82849	-0.65152	1.90425
H7	1.71268	-0.71131	-1.41439	2.43412	-0.33893	-0.51654
O1	1.36080	-0.50550	0.43054	0.67414	-0.95956	-0.78946
O2	1.51071	-1.36118	-0.72146	1.98824	-1.15147	-0.22581
Cl1	-0.03008	1.32843	-0.90091	0.61439	1.58022	0.04547
	CHPB-3 (*anti-cis*)			CHPB-4 (*anti-trans*-2)		
C1	2.22287	-0.52094	-0.68676	2.39522	0.39620	-0.63876
C2	0.93767	-0.83960	-0.71400	1.07998	0.55824	-0.66851
C3	-0.09139	-0.34862	0.25841	0.12234	-0.03085	0.31916
C4	-0.58224	-1.43007	1.20747	0.70711	-0.74933	1.51682
H1	2.62532	0.18789	0.02172	2.89930	-0.19772	0.11075
H2	2.91138	-0.96562	-1.39184	3.02126	0.86401	-1.38581
H3	0.55647	-1.54577	-1.44041	0.61673	1.15841	-1.43785
H4	-1.38738	-1.04927	1.83132	-0.09634	-1.13041	2.14148
H5	0.24890	-1.74911	1.83599	1.31247	-0.05455	2.09798
H6	-0.94216	-2.28260	0.63697	1.32280	-1.58262	1.19050
H7	-0.11954	2.22627	0.03402	-2.12893	1.11828	-0.41987
O1	0.28010	0.70917	1.08155	-0.76901	0.89858	0.86548
O2	0.72960	1.84844	0.31774	-1.39317	1.69681	-0.16047
Cl1	-1.58189	0.20899	-0.77134	-0.91046	-1.29318	-0.65350

	CHPB-5 (*syn-cis*)			CHPB-6 (*syn-trans*-2)		
C1	2.50400	0.24740	-0.44706	-2.46727	-0.35753	-0.42584
C2	1.54424	-0.42055	0.17670	-1.31560	-0.94929	-0.15558
C3	0.11259	0.00859	0.29060	0.00279	-0.29846	0.17315
C4	-0.35135	0.18170	1.72485	0.48463	-0.63773	1.57451
H1	2.31937	1.17984	-0.95972	-2.56795	0.71731	-0.45792
H2	3.51512	-0.13482	-0.45765	-3.35050	-0.94967	-0.62150
H3	1.75413	-1.35443	0.68169	-1.24829	-2.03169	-0.13721
H4	-0.14179	-0.72268	2.29080	-0.23691	-0.28031	2.30473
H5	0.19933	1.01385	2.16541	0.57350	-1.72154	1.66092
H6	-1.41475	0.39262	1.76870	1.45263	-0.18792	1.77014
H7	-1.86900	1.11038	-1.02859	2.26533	0.48692	-1.00826
O1	-0.05763	1.12757	-0.51474	0.85387	-0.75394	-0.84646
O2	-1.39030	1.65786	-0.38474	2.22563	-0.38357	-0.58185
Cl1	-0.91976	-1.40418	-0.45502	-0.10300	1.56030	0.05401
	CHPB-7			CHPB-8		
C1	2.09429	0.97808	-0.11930	2.40292	0.26536	-0.59547
C2	0.97557	0.87358	0.57825	1.08926	0.40576	-0.70606
C3	-0.12868	-0.13835	0.43093	0.08300	-0.08203	0.29414
C4	-0.28099	-1.01214	1.67086	0.61566	-0.82229	1.50629
H1	2.32040	0.31135	-0.93854	2.87300	-0.23119	0.24188
H2	2.81776	1.74635	0.11681	3.06188	0.64605	-1.36363
H3	0.78182	1.56288	1.39584	0.66099	0.89715	-1.56787
H4	0.61956	-1.60456	1.80843	1.15319	-1.71203	1.19084
H5	-0.43216	-0.38134	2.54698	1.28216	-0.17443	2.07473
H6	-1.13524	-1.67533	1.55524	-0.21456	-1.12048	2.14113
H7	-1.38242	2.23640	-0.39055	-0.83154	2.63303	0.03493
O1	-1.38815	0.47746	0.30047	-0.70419	0.94063	0.84445
O2	-1.39544	1.37644	-0.83416	-1.24864	1.79106	-0.19638
Cl1	0.15988	-1.24906	-1.01112	-1.02985	-1.25837	-0.64294

	CHPB-9			CHPB-10		
C1	-2.58424	-0.44383	-0.14112	-1.43131	1.94958	-0.13609
C2	-1.41141	-0.14555	-0.68055	-0.61573	1.03649	-0.64396
C3	-0.09744	-0.13581	0.04567	0.10000	-0.02916	0.13057
C4	-0.10204	-0.57895	1.49249	-0.09204	-0.04808	1.63120
H1	-2.69945	-0.71286	0.89935	-1.64720	2.01297	0.92132
H2	-3.48494	-0.41503	-0.73811	-1.91234	2.67753	-0.77474
H3	-1.33475	0.12579	-1.72551	-0.41930	1.00632	-1.70953
H4	-0.77549	0.04493	2.07378	0.22735	0.89427	2.07013
H5	-0.43074	-1.61755	1.55232	-1.14119	-0.21899	1.86480
H6	0.89564	-0.49495	1.91033	0.49817	-0.85538	2.05737
H7	2.18819	-1.94459	-0.23289	-1.93900	-1.47971	-1.05302
O1	0.72811	-0.90335	-0.78639	-0.14750	-1.32289	-0.45423
O2	2.07001	-0.98456	-0.23315	-1.53648	-1.66316	-0.19236
Cl1	0.49573	1.64367	0.00695	1.88469	0.14108	-0.24082

	CHPB-11			CHPB-12		
C1	2.49113	0.34523	-0.38089	-2.43265	-0.54306	-0.41020
C2	1.54129	-0.36174	0.21406	-1.25012	-1.02383	-0.06371
C3	0.08167	-0.01212	0.26987	0.02156	-0.25136	0.18858
C4	-0.43222	0.15716	1.68972	0.55520	-0.47259	1.59626
H1	2.28302	1.26062	-0.91480	-2.59849	0.51271	-0.56574
H2	3.51849	0.01015	-0.35158	-3.27387	-1.20930	-0.54314
H3	1.77763	-1.28173	0.73269	-1.11636	-2.09069	0.07991
H4	-0.18154	-0.72350	2.27598	-0.18487	-0.14071	2.32004
H5	0.05107	1.02713	2.13771	0.74102	-1.53799	1.74423
H6	-1.50903	0.29185	1.69844	1.47809	0.07743	1.74958
H7	-1.32158	2.48961	-0.23346	2.71797	-1.04982	-0.66765
O1	-0.10292	1.09873	-0.54755	0.87508	-0.71807	-0.82404
O2	-1.47411	1.56174	-0.46057	2.22745	-0.21810	-0.61411
Cl1	-0.82901	-1.47818	-0.47274	-0.23222	1.56899	-0.02810

	CHPB-13			CHPB-14		
C1	-1.21542	2.10333	-0.13896	-1.28485	1.89036	0.06216
C2	-0.55913	1.07470	-0.65520	-0.46058	1.11564	0.75360
C3	0.07725	-0.03662	0.12137	0.15407	-0.15542	0.23563
C4	-0.13661	-0.04659	1.62013	0.35489	-1.21437	1.30279
H1	-1.35014	2.23601	0.92553	-1.55050	1.66605	-0.96323
H2	-1.64654	2.85974	-0.77981	-1.69629	2.79188	0.49331
H3	-0.44668	0.97148	-1.72740	-0.18075	1.37908	1.76663
H4	0.24783	0.86627	2.06860	0.99102	-0.82504	2.09525
H5	-1.20001	-0.12821	1.83911	-0.60803	-1.49523	1.72514
H6	0.38785	-0.89451	2.05490	0.82361	-2.09366	0.86754
H7	-1.67883	-2.31164	0.19521	-2.38146	-0.48676	-0.58470
O1	-0.23433	-1.30431	-0.48053	-0.51888	-0.66303	-0.92069
O2	-1.67349	-1.50542	-0.33906	-1.78018	-1.24061	-0.49135
Cl1	1.87956	0.01822	-0.21785	1.78904	0.26327	-0.48404

	CHPB-15			CHPB-16		
C1	-1.40569	1.84362	-0.04118	1.56602	1.82818	-0.08618
C2	-0.41777	1.24204	0.60406	0.34031	1.36136	0.08840
C3	0.15457	-0.09398	0.23848	-0.09058	-0.07609	0.19168
C4	0.31135	-1.03001	1.42359	-0.54319	-0.45466	1.59329
H1	-1.87583	1.39676	-0.90507	2.42029	1.17651	-0.20090
H2	-1.77227	2.80635	0.28651	1.74517	2.89381	-0.12505
H3	0.04198	1.70442	1.46948	-0.49373	2.04548	0.19474
H4	0.94233	-0.56585	2.17859	0.28841	-0.35738	2.28751
H5	-0.66514	-1.23176	1.86020	-1.34096	0.21426	1.91287
H6	0.77252	-1.96372	1.10723	-0.90241	-1.48238	1.60652
H7	-1.71191	-2.12334	-0.58702	-2.91620	-0.24329	-0.30047
O1	-0.50288	-0.70607	-0.87255	-1.10106	-0.37047	-0.79829
O2	-1.81059	-1.17536	-0.42270	-2.27031	0.44808	-0.49973
Cl1	1.81890	0.19168	-0.49398	1.20795	-1.22475	-0.33613

Table S2. Cartesian coordinates of optimized geometries of *syn-trans*-MVKO⋯HCl (PRC1), *syn-cis*-MVKO⋯HCl (PRC3) and two conformers (*trans*- and *cis*-) of CMP [$(C_2H_3)CCl(CH_3)O$] predicted with the B3LYP/aug-cc-pVTZ method

atom	x	y	z		x	y	z
	syn-trans-MVKO…HCl				*syn-cis*-MVKO…HCl		
C1	0.97778	0.29542	0.11845	C1	-0.89281	0.51751	-0.08415
C2	2.15067	-0.01044	-0.66940	C2	-2.12432	-0.11955	-0.51098
C3	3.09199	-0.88810	-0.31248	C3	-2.58196	-1.25899	0.01592
C4	0.63268	-0.26177	1.43841	C4	-0.18974	1.50523	-0.90966
H1	2.22002	0.52285	-1.60884	H1	-2.63515	0.35880	-1.33492
H2	3.93943	-1.07536	-0.95612	H2	-3.49297	-1.70943	-0.35148
H3	3.05091	-1.44592	0.61177	H3	-2.04989	-1.77585	0.80219
H4	1.44716	-0.83854	1.86219	H4	-0.83876	1.89293	-1.68923
H5	0.34727	0.56045	2.09692	H5	0.22244	2.29452	-0.28085
H6	-0.25833	-0.88698	1.33795	H6	0.67884	0.99446	-1.35467
H7	-1.85766	0.38200	-0.05588	H7	1.47877	0.08312	0.74095
O1	0.21573	1.15268	-0.44226	O1	-0.47343	0.16116	1.07025
O2	-0.95592	1.49822	0.23174	O2	0.80420	0.65363	1.44840
Cl1	-2.59330	-0.77861	-0.29728	Cl1	2.27785	-0.73659	-0.45577
	trans-CMP				*cis*-CMP		
C1	-2.25106	-0.63589	-0.35461	C1	-2.32827	-0.62974	-0.03738
C2	-1.07571	-0.75656	0.24927	C2	-1.12963	-0.44709	-0.56838
C3	0.01385	0.31397	0.23486	C3	-0.01296	0.32044	0.10836
C4	-0.15969	1.46653	-0.75110	C4	0.37902	1.60393	-0.68795
H1	-2.54075	0.24525	-0.90865	H1	-2.58850	-0.24487	0.93823
H2	-2.96000	-1.45232	-0.32724	H2	-3.08769	-1.17761	-0.57758
H3	-0.81127	-1.63196	0.82346	H3	-0.86405	-0.84002	-1.54048
H4	-0.21298	1.08431	-1.76708	H4	0.64215	1.29454	-1.69627
H5	-1.06696	2.01661	-0.50452	H5	-0.47617	2.27563	-0.70512
H6	0.69126	2.13741	-0.66644	H6	1.23142	2.08255	-0.21514
O1	0.06840	0.70429	1.49468	O1	-0.26229	0.69538	1.33602
Cl1	1.59936	-0.60953	-0.28691	Cl1	1.51719	-0.82579	0.01296

Table S3. Cartesian coordinates of optimized geometries of four conformers of CPB [$(C_2H_3)CH(CH_3)OOCl$] predicted with the B3LYP/aug-cc-pVTZ method

atom	x	y	z	x	y	z
	CPB-1			CPB-2		
C1	1.84020	1.88212	-0.27700	-2.68884	-1.35997	-0.21686
C2	1.67717	0.72005	0.33883	-2.09253	-0.31031	0.33031
C3	0.89940	-0.42633	-0.22765	-0.82289	0.29487	-0.18692
C4	1.73238	-1.68799	-0.41286	-0.97039	1.76077	-0.56550
H1	1.38378	2.08299	-1.23868	-2.26787	-1.87058	-1.07421
H2	2.43393	2.67531	0.15626	-3.62284	-1.74373	0.17024
H3	2.13380	0.54157	1.30744	-2.53107	0.18252	1.19164
H4	1.11582	-2.50209	-0.79132	-1.66482	1.85413	-1.40037
H5	2.18055	-1.99737	0.53135	-1.35883	2.33711	0.27468
H6	2.53195	-1.49082	-1.12542	-0.01227	2.18040	-0.86557
H7	0.42810	-0.14041	-1.17138	-0.44539	-0.29084	-1.02859
O1	-0.16056	-0.84670	0.67781	0.12336	0.12271	0.91223
O2	-1.07923	0.19837	0.87065	1.38859	0.60624	0.54416
Cl1	-2.30497	0.18178	-0.38729	2.30914	-0.63487	-0.29913
	CPB-3			CPB-4		
C1	-3.13050	-0.67380	-0.03673	-2.86909	-1.01554	-0.18247
C2	-1.82561	-0.90623	-0.06378	-1.54786	-0.91299	-0.13842
C3	-0.75226	0.12542	-0.26138	-0.75587	0.35981	-0.22827
C4	-1.19324	1.57541	-0.29626	-1.53464	1.65459	-0.09036
H1	-3.54694	0.31892	-0.13942	-3.51909	-0.15648	-0.28053
H2	-3.83536	-1.48505	0.08221	-3.35236	-1.98123	-0.12481
H3	-1.45574	-1.91957	0.04432	-0.94424	-1.80845	-0.04702
H4	-1.82276	1.75866	-1.16703	-0.85620	2.50415	-0.13834
H5	-1.75078	1.83557	0.60287	-2.07502	1.68899	0.85478
H6	-0.32342	2.22497	-0.36807	-2.25223	1.74398	-0.90458
H7	-0.19631	-0.12386	-1.17213	-0.18427	0.37091	-1.16230
O1	0.16554	-0.12082	0.84776	0.23262	0.41596	0.84647
O2	1.37177	0.56362	0.63302	1.18047	-0.60229	0.68513
Cl1	2.47309	-0.40452	-0.34000	2.47785	-0.08156	-0.38899

Table S4. Cartesian coordinates of optimized geometries of TS1−TS8 predicted with the B3LYP/aug-cc-pVTZ method

atom	x	y	z	x	y	z
	TS1			TS2		
C1	0.71081	0.44648	0.08058	-0.59576	0.19837	-0.02963
C2	1.83716	-0.05060	-0.67778	-1.78567	-0.25438	0.70779
C3	2.73782	-0.89744	-0.17390	-2.87494	-0.77429	0.14631
C4	0.49899	0.19995	1.52428	-0.68646	0.53139	-1.50396
H1	1.88361	0.27031	-1.70971	-1.70738	-0.19286	1.78717
H2	3.54287	-1.27416	-0.78841	-3.69289	-1.13198	0.75501
H3	2.69251	-1.25410	0.84519	-2.98578	-0.87213	-0.92476
H4	1.45030	0.31101	2.04577	-0.89648	-0.37109	-2.07447
H5	-0.23943	0.89132	1.91565	-1.48801	1.25137	-1.67579
H6	0.12104	-0.81147	1.66916	0.26060	0.93496	-1.84680
H7	-1.70455	0.57872	-0.08942	0.32084	-0.64368	0.00859
O1	-0.00688	1.25679	-0.61700	0.12475	1.09422	0.76752
O2	-1.27003	1.57693	-0.06412	1.29868	1.35028	0.27852
Cl1	-1.89646	-1.15128	-0.17402	2.02698	-0.98454	-0.01884
	TS3			TS4		
C1	2.22532	-1.33401	-0.36063	-0.63105	0.43086	-0.16131
C2	1.93582	-0.27723	0.40399	-1.94931	-0.19904	-0.38560
C3	0.76777	0.54787	0.17657	-2.28587	-1.39785	0.08558
C4	0.19117	1.38523	1.24682	-0.38070	1.79588	-0.76726
H1	2.52425	-0.03798	1.27903	-2.64780	0.36381	-0.99352
H2	3.08466	-1.95316	-0.14551	-3.26723	-1.81278	-0.09568
H3	1.59253	-1.62156	-1.18821	-1.59527	-1.99627	0.66481
H4	1.00118	1.76565	1.87024	-1.13659	2.50140	-0.41512
H5	-0.40815	2.18793	0.82776	0.61030	2.15276	-0.50765
H6	-0.46270	0.76283	1.86134	-0.45133	1.72535	-1.85152
H7	-1.48070	0.34154	-0.70864	0.29887	-0.25592	-0.60255
O1	0.37821	0.58608	-1.04751	-0.21934	0.25921	1.16583
O2	-0.92902	1.11273	-1.23817	1.02386	0.58976	1.33749
Cl1	-1.89207	-0.99806	0.33464	1.95497	-0.77936	-0.52082

	TS5			TS6		
C1	2.60505	0.06678	-0.25663	-2.29749	-1.40985	0.13510
C2	1.44980	-0.56773	-0.39162	-1.21152	-0.77007	0.55954
C3	0.14087	-0.01846	0.13201	-0.62373	0.40995	-0.11340
C4	0.13094	0.34658	1.60416	-1.46101	1.25964	-1.03101
H1	2.69171	1.01871	0.24965	-2.84130	-1.11723	-0.75264
H2	3.51374	-0.35173	-0.66661	-2.67542	-2.26615	0.67562
H3	1.38976	-1.50645	-0.92253	-0.65618	-1.11979	1.41967
H4	0.38956	-0.52459	2.20023	-0.89111	2.12442	-1.36401
H5	0.87098	1.12827	1.77766	-2.37498	1.59679	-0.54164
H6	-0.84575	0.71397	1.90245	-1.73979	0.68177	-1.91106
H7	-2.03453	1.20239	-0.77989	0.36857	-0.09489	-0.67943
O1	-0.15229	1.05225	-0.72433	0.18082	1.22295	0.68718
O2	-1.36520	1.72607	-0.31138	1.06895	0.53693	1.34031
Cl1	-1.16444	-1.34530	-0.11781	2.02203	-0.63659	-0.60987

	TS7			TS8		
C1	0.86460	-0.35178	-0.12242	-2.21534	-0.70083	-0.66685
C2	1.28490	1.05387	-0.37137	-0.97193	-0.99483	-0.31875
C3	0.55222	2.14847	-0.18428	-0.03302	-0.07140	0.40804
C4	1.82511	-1.43035	-0.55370	-0.52550	0.40763	1.76251
H1	2.29833	1.14128	-0.74958	-2.64247	0.28261	-0.51929
H2	0.99200	3.12494	-0.33803	-2.84562	-1.44048	-1.13997
H3	-0.48514	2.10534	0.10918	-0.56022	-1.97634	-0.51028
H4	1.44348	-2.41547	-0.29500	0.21443	1.05682	2.22443
H5	1.94989	-1.38379	-1.63505	-0.70247	-0.45730	2.40141
H6	2.80622	-1.29633	-0.09524	-1.45787	0.95188	1.64083
H7	-0.24751	-0.44090	-0.65547	2.09909	-0.40252	-0.98413
O1	0.36268	-0.66467	1.14684	1.20862	-0.65047	0.66198
O2	-0.61712	0.10311	1.49093	1.76737	-1.20489	-0.54659
Cl1	-1.99311	-0.28611	-0.59131	0.26836	1.46969	-0.65564

Table S5. Comparison of experimentally observed wavenumbers (cm^{-1}) and intensities of (*Z*)-(CH_2I)HCC(CH_3)I with harmonic and anharmonic vibrational wavenumbers and infrared intensities predicted with the B3LYP/aug-cc-pVTZ-pp method

mode	experiment		harmonic		scaled harmonic		anharmonic	
	ν / cm^{-1}	int.[a]	ν / cm^{-1}	int.[b]	ν^c / cm^{-1}	dev.[d]	ν / cm^{-1}	dev.[d]
ν_2	3010	(8)	3117	6.7	3007	−3	3003	−7
ν_4	2974	(28)	3093	12.0	2985	11	2963	−11
ν_5	2965		3081	5.9	2973	8	2942	−23
ν_6	2929	(15)	3024	17.0	2919	−10	2919	−10
ν_7	1641	(17)	1684	33.5	1646	5	1640	−1
ν_{10}	1434	(22)	1467	12.5	1435	1	1425	−9
ν_{11}	1384	(3)	1415	2.1	1384	0	1382	−2
ν_{12}	1295	(29)	1330	29.3	1302	7	1304	9
ν_{13}	1169	(100)	1182	30.8	1158	−11	1154	−15
ν_{14}	1153		1168	73.3	1145	−8	1139	−14
ν_{16}	1062	(25)	1071	31.9	1050	−12	1047	−15
ν_{19}	837	(17)	869	15.3	854	17	854	17
					Avg.[e] 7.8 ± 4.9 cm^{-1}		Avg.[e] 11.1 ± 6.2 cm^{-1}	

[a]Integrated IR intensities relative to the overlapped bands of 1153 and 1169 cm^{-1}. [b]Harmonic IR intensity in unit of km mol^{-1}. [c]Harmonic vibrational wavenumbers x under 2000 cm^{-1} are scaled according to $y = 0.971\,x + 10.4$, and those larger than 2000 cm^{-1} according to $y = 0.955\,x + 30.7$. [d]deviation = predicted vibrational wavenumber – experimental value. [e]Average absolute deviations; the uncertainty represents one standard deviation in fitting.

Table S6. Vibrational wavenumbers and IR intensities of six lowest-energy conformers of CHPB predicted with the B3LYP/aug-cc-pVTZ method

mode	CHPB-1 (*anti-trans*-1)			CHPB-2 (*syn-trans*-1)		
	harm.[a]	int.[b]	anharm.	harm.[a]	int.[b]	anharm.
ν_1	3555	30.3	3506	3553	32.9	3495
ν_2	3120	3.3	3118	3118	4.6	3096
ν_3	3043	2.7	3008	3062	0.7	3048
ν_4	3035	5.2	3012	3049	2.3	3021
ν_5	3030	2.9	3014	3040	3.2	2995
ν_6	3012	6.9	2997	3019	5.7	3007
ν_7	2944	7.2	2941	2948	7.4	2940
ν_8	1674	3.5	1676	1665	0.4	1662
ν_9	1455	2.0	1447	1461	3.6	1459
ν_{10}	1448	2.3	1441	1457	3.4	1449
ν_{11}	1416	18.8	1415	1421	17.3	1416
ν_{12}	1377	7.0	1383	1381	16.9	1380
ν_{13}	1370	62.3	1359	1370	59.9	1358
ν_{14}	1297	2.4	1303	1305	0.6	1313
ν_{15}	1202	37.5	1196	1254	12.0	1250
ν_{16}	1125	72.9	1118	1146	49.9	1141
ν_{17}	1083	36.2	1073	1070	69.7	1070
ν_{18}	1026	7.0	1028	1012	13.5	1025
ν_{19}	999	8.1	997	1003	6.4	997
ν_{20}	964	37.8	962	962	39.2	958
ν_{21}	951	5.7	941	933	1.5	926
ν_{22}	903	11.9	900	895	32.5	891
ν_{23}	805	61.5	802	811	77.7	810
ν_{24}	693	12.3	691	692	18.9	691
ν_{25}	572	29.9	568	583	11.9	580
ν_{26}	522	2.7	518	505	49.7	495
ν_{27}	471	30.5	470	430	12.0	427
ν_{28}	394	12.3	389	382	16.8	375
ν_{29}	330	6.1	334	344	6.5	340
ν_{30}	307	46.9	352	324	25.4	157
ν_{31}	299	28.8	299	288	69.1	404
ν_{32}	250	33.7	193	259	4.3	178
ν_{33}	242	0.0	242	251	6.0	290
ν_{34}	210	6.6	166	224	16.6	269
ν_{35}	150	12.3	148	150	18.5	132
ν_{36}	87	0.5	68	98	0.3	65

mode	CHPB-3 (*anti-cis*)			CHPB-4 (*anti-trans*-2)		
	harm [a]	int. [b]	anharm.	harm. [a]	int. [b]	anharm.
ν_1	3546	31.0	3491	3555	33.4	3498
ν_2	3124	2.7	3103	3114	5.4	3127
ν_3	3059	2.2	3045	3085	0.4	3059
ν_4	3041	3.6	2993	3039	6.3	2971
ν_5	3030	5.1	3002	3036	1.5	3052
ν_6	3014	5.6	3006	3020	4.9	3029
ν_7	2945	5.4	2933	2951	5.2	2958
ν_8	1663	0.5	1659	1662	0.7	1683
ν_9	1454	3.2	1445	1459	2.3	1453
ν_{10}	1448	1.7	1439	1457	3.3	1448
ν_{11}	1422	15.9	1416	1423	19.5	1417
ν_{12}	1380	70.8	1360	1381	9.3	1391
ν_{13}	1378	3.3	1382	1374	66.1	1367
ν_{14}	1303	0.5	1306	1304	1.4	1306
ν_{15}	1232	44.9	1225	1253	22.1	1240
ν_{16}	1125	21.5	1114	1151	51.5	1147
ν_{17}	1061	72.7	1057	1070	67.4	1061
ν_{18}	1038	9.2	1042	1016	12.4	1037
ν_{19}	1006	9.8	995	993	5.0	978
ν_{20}	963	37.4	963	957	37.2	957
ν_{21}	956	4.1	945	949	7.2	941
ν_{22}	902	10.7	898	901	16.6	897
ν_{23}	782	56.9	777	775	84.6	774
ν_{24}	688	38.8	688	721	23.0	717
ν_{25}	641	41.9	643	601	16.7	605
ν_{26}	526	9.7	523	479	44.5	481
ν_{27}	401	10.3	395	443	13.3	442
ν_{28}	376	39.0	339	381	9.2	383
ν_{29}	326	8.6	295	333	14.3	326
ν_{30}	301	15.8	405	314	46.6	339
ν_{31}	294	1.8	286	285	44.6	409
ν_{32}	246	63.2	202	267	14.8	252
ν_{33}	242	13.7	251	250	7.0	176
ν_{34}	212	5.7	162	234	8.8	211
ν_{35}	174	19.2	116	152	16.2	158
ν_{36}	112	0.1	97	91	0.9	86

mode	CHPB-5 (*syn-cis*)			CHPB-6 (*syn-trans*-2)		
	harm.[a]	int.[b]	anharm.	harm.[a]	int.[b]	anharm.
ν_1	3555	33.0	3497	3572	35.4	3519
ν_2	3125	2.4	3095	3124	2.3	3119
ν_3	3062	1.4	3034	3045	3.6	3015
ν_4	3044	2.5	3021	3044	4.8	2995
ν_5	3042	4.7	2995	3033	3.7	3008
ν_6	3013	6.6	2999	3013	6.4	2984
ν_7	2944	7.2	2935	2943	9.3	2924
ν_8	1660	1.9	1658	1672	1.2	1668
ν_9	1457	3.2	1455	1458	3.0	1459
ν_{10}	1448	1.7	1439	1448	1.9	1441
ν_{11}	1421	14.7	1424	1416	17.0	1411
ν_{12}	1380	14.3	1378	1377	13.6	1361
ν_{13}	1377	65.7	1364	1363	58.8	1352
ν_{14}	1307	1.4	1317	1300	2.8	1303
ν_{15}	1221	25.9	1214	1196	30.8	1198
ν_{16}	1164	41.9	1159	1121	68.3	1112
ν_{17}	1058	65.8	1054	1095	40.8	1087
ν_{18}	1036	7.5	1045	1023	7.2	1030
ν_{19}	1006	11.3	956	1000	10.4	1011
ν_{20}	964	37.1	970	973	39.1	967
ν_{21}	954	4.0	939	924	4.1	917
ν_{22}	903	15.0	901	883	18.1	885
ν_{23}	802	76.8	801	827	65.7	821
ν_{24}	691	29.6	695	675	8.1	670
ν_{25}	593	16.0	593	605	26.4	597
ν_{26}	516	36.8	506	521	24.0	516
ν_{27}	456	12.5	455	417	29.4	407
ν_{28}	366	31.2	355	397	4.7	384
ν_{29}	333	11.3	321	351	2.1	341
ν_{30}	304	14.3	283	331	5.4	317
ν_{31}	296	42.1	369	285	63.7	138
ν_{32}	263	29.9	118	259	20.2	409
ν_{33}	247	8.2	257	239	14.2	198
ν_{34}	214	3.8	219	227	8.3	226
ν_{35}	140	15.8	134	143	13.0	108
ν_{36}	82	1.0	85	75	0.1	17

[a]Harmonic vibrational wavenumbers x under 2000 cm^{-1} are scaled according to $y = 0.971\,x + 10.4$, and those larger than 2000 cm^{-1} according to $y = 0.955\,x + 30.7$. [b]Harmonic IR intensity in unit of km mol^{-1}.

Table S7. Vibrational wavenumbers and IR intensities of four conformers of CPB predicted with the B3LYP/aug-cc-pVTZ method

mode	CPB-1			CPB-2		
	harm.[a]	int.[b]	anharm.	harm.	int.	anharm.
ν_1	3102	9.4	3072	3104	9.2	3076
ν_2	3028	2.7	3028	3034	4.8	3020
ν_3	3021	10.8	2971	3023	8.0	2979
ν_4	3008	11.9	2968	3011	10.9	2991
ν_5	3002	13.0	2980	2997	14.7	2965
ν_6	2936	12.6	2856	2935	14.3	2935
ν_7	2929	5.7	2883	2931	4.0	2911
ν_8	1670	2.0	1658	1669	1.3	1670
ν_9	1465	2.2	1464	1466	5.0	1457
ν_{10}	1454	7.2	1447	1457	4.1	1451
ν_{11}	1432	11.9	1430	1432	10.4	1429
ν_{12}	1377	6.0	1368	1377	12.8	1371
ν_{13}	1317	11.0	1317	1324	10.6	1324
ν_{14}	1302	10.7	1259	1304	10.5	1314
ν_{15}	1291	1.2	1292	1291	5.8	1285
ν_{16}	1178	7.7	1181	1185	2.8	1182
ν_{17}	1129	18.3	1131	1103	8.4	1098
ν_{18}	1043	30.6	1043	1046	49.4	1039
ν_{19}	1011	10.4	1026	1012	10.8	972
ν_{20}	971	6.5	974	978	3.7	993
ν_{21}	958	37.5	949	962	43.0	963
ν_{22}	868	39.6	869	865	16.6	853
ν_{23}	864	19.3	873	856	32.6	853
ν_{24}	815	4.0	810	833	7.3	829
ν_{25}	695	10.6	697	669	5.6	670
ν_{26}	608	45.9	596	597	49.4	597
ν_{27}	520	6.7	513	535	7.2	535
ν_{28}	485	1.7	476	484	5.2	481
ν_{29}	377	3.2	373	375	2.6	373
ν_{30}	333	1.9	327	325	1.8	320
ν_{31}	286	1.4	273	280	7.8	286
ν_{32}	247	5.8	242	265	1.1	260
ν_{33}	212	0.1	211	218	0.0	179
ν_{34}	120	0.7	74	114	0.5	109
ν_{35}	85	0.2	15	84	0.1	84
ν_{36}	59	0.2	9	59	0.1	45

mode	CPB-3			CPB-4		
	harm.	int.	anharm.	harm.	int.	anharm.
ν_{1}	3111	7.6	3089	3106	8.9	3083
ν_{2}	3043	3.6	3034	3046	1.5	3040
ν_{3}	3033	6.4	2981	3032	7.1	2977
ν_{4}	3013	9.5	2993	3012	12.2	2994
ν_{5}	3000	12.9	2982	3007	9.8	2982
ν_{6}	2938	10.4	2944	2942	9.2	2948
ν_{7}	2897	5.8	2878	2904	5.8	2877
ν_{8}	1669	3.4	1665	1667	4.7	1671
ν_{9}	1468	5.7	1459	1468	2.8	1459
ν_{10}	1463	3.8	1457	1461	7.3	1452
ν_{11}	1424	11.4	1422	1426	11.2	1431
ν_{12}	1379	14.7	1382	1381	9.1	1378
ν_{13}	1348	3.5	1342	1344	6.4	1335
ν_{14}	1308	13.9	1319	1312	24.3	1313
ν_{15}	1297	13.2	1290	1295	7.1	1290
ν_{16}	1157	8.5	1154	1166	1.6	1159
ν_{17}	1084	20.7	1084	1098	28.7	1094
ν_{18}	1058	11.8	1056	1049	12.0	1047
ν_{19}	1016	14.6	1009	1019	10.8	1014
ν_{20}	985	8.0	986	980	5.0	977
ν_{21}	959	38.7	956	953	39.0	948
ν_{22}	860	42.8	848	869	47.2	865
ν_{23}	856	13.0	851	857	8.7	847
ν_{24}	833	4.5	833	808	7.1	806
ν_{25}	661	5.5	663	687	10.0	683
ν_{26}	594	48.8	591	593	45.8	590
ν_{27}	541	2.8	543	563	7.2	562
ν_{28}	466	2.6	464	453	7.0	450
ν_{29}	438	10.1	441	410	4.9	406
ν_{30}	323	2.7	322	323	3.6	316
ν_{31}	283	4.1	305	307	1.9	313
ν_{32}	259	3.3	257	237	4.4	278
ν_{33}	240	0.2	212	223	1.3	163
ν_{34}	125	0.4	130	130	0.5	121
ν_{35}	85	0.2	70	90	0.2	38
ν_{36}	65	0.0	53	63	0.0	54

[a]Harmonic vibrational wavenumbers x under 2000 cm^{-1} are scaled according to $y = 0.971\ x + 10.4$, and those larger than 2000 cm^{-1} according to $y = 0.955\ x + 30.7$. [b]Harmonic IR intensity in unit km mol^{-1}.

Table S8. Vibrational wavenumbers and IR intensities of two conformers of CMP $(C_2H_3)CCl(CH_3)O$ predicted with the B3LYP/aug-cc-pVTZ method

mode	*trans*-CMP			*cis*-CMP		
	harm.[a]	int.[b]	anharm.	harm.	int.	anharm.
ν_1	3119	1.6	3100	3121	1.0	3103
ν_2	3090	1.8	3070	3069	0.5	3053
ν_3	3039	2.0	3036	3044	3.8	3034
ν_4	3035	2.5	3013	3039	3.2	2991
ν_5	3019	6.3	3005	3033	5.0	3016
ν_6	2946	8.6	2939	2950	4.6	2956
ν_7	1607	6.0	1614	1646	11.9	1654
ν_8	1458	6.0	1450	1452	4.7	1443
ν_9	1446	3.3	1433	1429	4.3	1421
ν_{10}	1400	23.3	1394	1405	8.8	1401
ν_{11}	1363	1.6	1357	1355	4.9	1361
ν_{12}	1290	3.2	1290	1290	2.7	1293
ν_{13}	1184	42.9	1148	1162	38.3	1127
ν_{14}	1082	0.1	1053	1052	55.4	1046
ν_{15}	1065	62.7	1059	1023	8.7	1023
ν_{16}	988	14.2	984	995	18.9	974
ν_{17}	977	5.4	952	967	5.6	962
ν_{18}	955	39.8	953	962	34.8	955
ν_{19}	921	28.7	921	880	4.3	848
ν_{20}	766	41.2	749	773	61.6	764
ν_{21}	663	45.5	655	679	31.1	677
ν_{22}	558	36.7	552	592	33.8	581
ν_{23}	428	23.7	422	427	23.3	417
ν_{24}	353	26.0	315	359	20.4	354
ν_{25}	349	10.1	345	349	23.0	335
ν_{26}	330	3.9	324	293	2.7	315
ν_{27}	277	0.7	253	273	2.3	266
ν_{28}	270	0.9	336	253	0.6	279
ν_{29}	237	1.3	164	241	0.3	174
ν_{30}	78	1.4	68	95	0.9	84

[a]Harmonic vibrational wavenumbers x under 2000 cm^{-1} are scaled according to $y = 0.971\,x + 10.4$, and those larger than 2000 cm^{-1} according to $y = 0.955\,x + 30.7$. [b]Harmonic IR intensity in unit km mol^{-1}.

Table S9. Rotational parameters and *a*-/*b*-/*c*-type ratios for each vibrational state of six lowest-energy conformers of CHPB predicted with the B3LYP/aug-cc-pVTZ method

mode	CHPB-1 (*anti*-*trans*-1)				CHPB-2 (*syn*-*trans*-1)			
	A'/*A*"	*B*'/*B*"	*C*'/*C*"	*a*-/*b*-/*c*-ratio	*A*'/*A*"	*B*'/*B*"	*C*'/*C*"	*a*-/*b*-/*c*-ratio
	A" / cm^{-1}	*B*" / cm^{-1}	*C*" / cm^{-1}		*A*" / cm^{-1}	*B*" / cm^{-1}	*C*" / cm^{-1}	
	0.07124	0.06609	0.05813		0.08733	0.06011	0.04732	
ν_1	1.00039	1.00009	1.00146	15/13/72	1.00313	0.99860	1.00055	59/36/6
ν_2	0.99999	0.99973	0.99917	27/71/2	0.99982	0.99955	0.99970	19/4/78
ν_3	0.99934	0.99997	0.99959	48/48/4	1.00017	0.99937	0.99977	38/0/62
ν_4	0.99969	1.00008	1.00019	86/0/14	1.00016	0.99947	1.00004	97/1/3
ν_5	0.99829	1.00035	1.00058	32/14/54	0.99982	0.99948	0.99973	78/1/21
ν_6	1.00001	0.99994	0.99988	12/71/17	1.00001	0.99963	0.99987	1/85/14
ν_7	0.99992	0.99992	0.99986	1/0/99	0.99979	0.99978	0.99977	30/46/24
ν_8	1.00056	0.99806	0.99842	59/41/0	0.99992	0.99827	0.99854	3/83/15
ν_9	1.00004	1.00097	1.00002	85/12/3	1.00129	1.00003	0.99989	4/69/26
ν_{10}	1.00133	1.00000	0.99979	2/56/43	1.00010	1.00050	0.99985	2/7/91
ν_{11}	0.99989	1.00017	0.99971	81/1/18	1.00001	0.99997	0.99987	90/0/9
ν_{12}	0.99839	0.99849	0.99988	0/28/72	0.99831	0.99860	0.99994	3/1/96
ν_{13}	0.99921	0.99956	0.99873	14/32/55	0.99833	1.00012	0.99894	95/3/2
ν_{14}	1.00077	0.99980	0.99998	2/55/43	1.00015	1.00003	1.00030	36/9/55
ν_{15}	0.99912	0.99935	1.00014	87/13/0	0.99993	0.99859	0.99918	15/6/80
ν_{16}	0.99841	1.00032	0.99950	46/48/7	0.99920	0.99837	0.99987	74/18/8
ν_{17}	0.99985	0.99605	0.99725	5/12/83	0.99943	0.99940	0.99941	4/95/1
ν_{18}	0.99942	1.00197	1.00077	9/64/27	0.99970	0.99985	0.99977	19/50/31
ν_{19}	1.00028	0.99722	0.99964	16/33/51	1.00007	0.99955	0.99994	1/51/48
ν_{20}	0.99994	0.99953	0.99938	23/17/60	0.99891	1.00008	0.99958	0/94/6
ν_{21}	0.99818	0.99961	1.00005	64/31/5	0.99967	0.99907	0.99896	34/47/19
ν_{22}	0.99861	0.99870	0.99972	77/8/15	0.99913	0.99797	0.99877	42/49/8
ν_{23}	0.99898	0.99834	0.99807	11/67/22	0.99752	0.99892	0.99861	2/94/4
ν_{24}	1.00167	0.99832	0.99811	8/32/60	1.00001	0.99958	0.99962	4/96/0
ν_{25}	0.99770	0.99986	1.00055	3/59/38	0.99968	0.99968	0.99954	25/75/0
ν_{26}	0.99961	0.99997	0.99991	12/58/30	0.99977	0.99894	0.99909	1/97/1
ν_{27}	0.99861	0.99970	0.99905	10/77/12	0.99768	1.00035	0.99894	25/74/1
ν_{28}	0.99937	0.99918	0.99780	8/17/75	0.99942	1.00005	0.99896	20/50/30
ν_{29}	1.00086	1.00014	1.00105	76/18/6	0.99703	0.99958	1.00030	26/63/10
ν_{30}	0.99992	0.99896	0.99835	99/1/0	0.99921	0.99932	0.99973	16/48/36
ν_{31}	0.99973	0.99912	0.99895	95/2/3	0.99868	0.99972	0.99922	24/4/72
ν_{32}	0.99888	1.00012	0.99893	99/0/1	0.99985	0.99938	0.99947	54/8/38
ν_{33}	0.99855	0.99941	1.00134	83/5/12	1.00050	0.99937	0.99937	67/2/31
ν_{34}	1.00091	0.99868	0.99948	93/2/4	1.00053	0.99889	0.99949	42/0/58
ν_{35}	0.99910	1.00023	0.99677	33/56/12	0.99489	1.00215	0.99856	67/29/4
ν_{36}	0.99872	1.00133	1.00046	79/3/18	0.99861	1.00186	1.00002	32/44/23

mode	CHPB-3 (*anti-cis*)				CHPB-4 (*anti-trans*-2)			
	A'/*A*"	*B*'/*B*"	*C*'/*C*"	*a*-/*b*-/*c*-ratio	*A*'/*A*"	*B*'/*B*"	*C*'/*C*"	*a*-/*b*-/*c*-ratio
	A" / cm^{-1}	*B*" / cm^{-1}	*C*" / cm^{-1}		*A*" / cm^{-1}	*B*" / cm^{-1}	*C*" / cm^{-1}	
	0.08296	0.06153	0.05388		0.07656	0.06332	0.05196	
ν_{1}	0.99931	1.00050	1.00111	48/37/15	1.00353	0.99741	1.00106	80/6/14
ν_{2}	0.99995	0.99976	0.99918	14/24/62	0.99982	0.99954	0.99983	3/29/67
ν_{3}	0.99893	0.99992	1.00020	0/76/24	1.00050	0.99877	0.99942	61/23/16
ν_{4}	0.99970	0.99974	0.99955	95/0/5	0.99969	0.99968	0.99987	90/2/7
ν_{5}	1.00010	1.00015	0.99980	20/22/58	0.99978	0.99986	0.99998	0/1/99
ν_{6}	0.99982	0.99997	1.00002	90/1/9	0.99980	0.99986	0.99994	39/56/5
ν_{7}	0.99989	0.99990	0.99991	1/44/55	0.99976	0.99983	0.99990	38/1/61
ν_{8}	1.00047	0.99828	0.99846	63/0/37	1.00012	0.99814	0.99846	97/1/2
ν_{9}	1.00111	1.00010	1.00000	18/20/62	1.00001	1.00039	1.00008	22/23/55
ν_{10}	1.00027	1.00037	1.00032	19/81/0	1.00107	1.00003	1.00017	3/76/21
ν_{11}	1.00029	0.99989	0.99954	95/1/3	0.99987	1.00006	0.99994	97/2/1
ν_{12}	0.99969	0.99954	0.99881	5/86/9	0.99852	0.99859	0.99963	0/24/76
ν_{13}	0.99836	0.99919	0.99926	24/21/55	0.99779	1.00082	0.99904	38/34/28
ν_{14}	1.00104	1.00023	0.99942	27/61/12	1.00064	0.99973	1.00004	89/1/10
ν_{15}	0.99996	0.99966	0.99879	0/40/60	1.00012	0.99853	0.99906	38/1/61
ν_{16}	0.99784	0.99954	0.99863	72/28/0	0.99892	0.99896	0.99931	58/31/11
ν_{17}	0.99855	0.99880	0.99959	65/9/25	0.99901	0.99932	0.99963	16/64/21
ν_{18}	0.99976	1.00120	1.00046	6/76/18	0.99974	1.00069	1.00164	9/64/27
ν_{19}	1.00025	0.99880	0.99892	0/46/54	1.00020	0.99847	0.99740	18/16/67
ν_{20}	0.99896	0.99989	0.99991	8/54/38	0.99845	1.00002	1.00008	1/68/31
ν_{21}	0.99948	0.99995	0.99872	4/35/62	0.99926	0.99942	0.99906	2/25/73
ν_{22}	0.99834	0.99982	0.99905	0/92/8	0.99868	0.99885	0.99921	78/19/3
ν_{23}	0.99750	0.99813	0.99920	66/0/34	0.99790	0.99888	0.99873	5/77/17
ν_{24}	1.00075	0.99940	0.99942	40/18/42	0.99969	0.99924	1.00008	8/71/21
ν_{25}	0.99755	0.99911	1.00013	88/2/10	0.99867	0.99987	0.99944	36/32/32
ν_{26}	0.99973	0.99914	0.99915	82/4/14	0.99988	0.99907	0.99915	6/80/14
ν_{27}	0.99929	0.99867	1.00007	15/11/74	0.99877	0.99962	0.99877	25/37/38
ν_{28}	0.99978	0.99880	0.99865	43/7/50	0.99798	1.00017	0.99963	0/19/81
ν_{29}	0.99942	1.00047	1.00095	30/65/6	1.00004	0.99967	1.00006	2/58/40
ν_{30}	0.99980	0.99946	0.99820	0/41/58	0.99837	1.00093	0.99981	58/19/23
ν_{31}	0.99965	0.99868	1.00017	87/8/5	0.99828	0.99942	0.99942	81/0/19
ν_{32}	0.99937	0.99980	1.00002	11/52/37	0.99907	0.99951	0.99950	63/24/13
ν_{33}	0.99836	0.99979	1.00052	8/59/33	0.99884	0.99841	1.00090	82/1/17
ν_{34}	0.99967	1.00132	0.99900	13/56/31	1.00150	0.99766	1.00031	81/0/19
ν_{35}	1.00012	0.99696	0.99631	12/79/9	0.99561	1.00276	0.99759	41/59/0
ν_{36}	0.99752	1.00018	1.00089	84/3/13	0.99922	1.00204	1.00098	45/52/3

mode	CHPB-5 (*syn-cis*)				CHPB-6 (*syn-trans*-2)			
	A'/*A*"	*B*'/*B*"	*C*'/*C*"	*a*-/*b*-/*c*-ratio	*A*'/*A*"	*B*'/*B*"	*C*'/*C*"	*a*-/*b*-/*c*-ratio
	A" / cm^{-1}	*B*" / cm^{-1}	*C*" / cm^{-1}		*A*" / cm^{-1}	*B*" / cm^{-1}	*C*" / cm^{-1}	
	0.07902	0.06399	0.04992		0.08989	0.0603	0.04939	
ν_1	1.00338	0.99739	1.00248	53/7/40	0.99990	1.00095	1.00085	26/55/19
ν_2	1.00005	0.99952	0.99952	69/29/2	0.99987	0.99964	0.99951	46/52/3
ν_3	0.99989	0.99925	1.00022	10/47/43	0.99960	0.99965	0.99955	52/47/1
ν_4	0.99995	0.99980	0.99996	87/11/2	0.99983	0.99968	1.00012	89/11/0
ν_5	1.00003	0.99953	0.99954	93/0/7	0.99947	0.99967	0.99966	39/55/6
ν_6	1.00003	0.99978	1.00016	5/92/3	0.99958	0.99998	1.00022	8/74/18
ν_7	0.99981	0.99977	1.00008	11/12/77	0.99967	0.99998	0.99996	0/64/35
ν_8	0.99963	0.99861	0.99866	96/3/1	1.00028	0.99833	0.99887	54/37/9
ν_9	1.00128	1.00005	0.99972	0/82/17	1.00072	1.00051	0.99984	19/25/56
ν_{10}	1.00001	1.00108	0.99994	74/20/6	1.00059	1.00055	1.00004	45/55/0
ν_{11}	1.00018	0.99995	0.99946	61/21/18	1.00006	0.99998	0.99957	84/13/2
ν_{12}	0.99860	0.99836	1.00000	23/0/77	0.99844	0.99864	0.99980	7/0/93
ν_{13}	0.99822	1.00061	0.99828	74/23/3	0.99949	0.99899	0.99907	95/4/1
ν_{14}	1.00009	1.00045	0.99960	0/82/18	1.00093	1.00022	0.99966	26/73/1
ν_{15}	0.99982	0.99950	0.99866	46/53/1	0.99962	0.99942	0.99919	21/65/14
ν_{16}	0.99932	0.99814	0.99866	13/54/33	1.00056	0.99806	0.99964	48/14/38
ν_{17}	0.99922	0.99911	0.99978	24/51/25	0.99919	0.99849	0.99812	6/75/19
ν_{18}	1.00022	1.00105	1.00064	69/12/19	0.99959	1.00224	1.00014	21/74/5
ν_{19}	0.99913	0.99834	1.00016	0/20/79	0.99954	0.99763	1.00012	13/0/86
ν_{20}	0.99934	0.99986	0.99982	7/25/68	1.00008	0.99919	0.99947	6/0/93
ν_{21}	0.99961	0.99887	0.99854	51/49/0	0.99849	0.99887	1.00002	61/34/5
ν_{22}	0.99887	0.99884	0.99860	10/78/11	0.99869	0.99799	0.99933	63/1/36
ν_{23}	0.99747	0.99917	0.99764	13/82/5	0.99801	0.99828	0.99830	10/83/7
ν_{24}	0.99999	0.99959	0.99980	8/48/44	1.00000	0.99955	1.00008	1/14/85
ν_{25}	0.99814	0.99964	1.00074	59/39/1	0.99930	0.99962	0.99935	18/82/0
ν_{26}	0.99892	0.99866	1.00128	5/95/0	0.99839	0.99967	0.99858	0/100/0
ν_{27}	1.00032	1.00006	0.99708	5/89/6	0.99851	1.00013	0.99919	17/72/12
ν_{28}	0.99787	0.99930	0.99850	40/60/0	0.99891	1.00048	0.99960	62/38/0
ν_{29}	0.99871	1.00005	1.00124	0/20/80	0.99695	1.00030	1.00099	5/90/5
ν_{30}	0.99900	0.99992	0.99922	6/45/49	1.00029	0.99879	0.99937	12/1/87
ν_{31}	0.99860	0.99978	1.00006	0/41/58	0.99991	0.99920	0.99947	17/7/76
ν_{32}	0.99995	0.99908	1.00040	4/7/89	0.99955	0.99973	1.00057	8/2/90
ν_{33}	0.99981	0.99953	0.99972	18/18/65	0.99907	1.00002	0.99854	18/0/82
ν_{34}	1.00123	0.99886	0.99920	1/19/80	1.00036	0.99975	0.99966	11/2/87
ν_{35}	0.99462	1.00334	0.99685	27/65/8	0.99880	0.99952	0.99824	80/9/11
ν_{36}	0.99958	1.00072	1.00086	4/94/1	0.99735	1.00078	1.00154	2/3/95

Table S10. Rotational parameters and *a*-/*b*-/*c*-type ratios for each vibrational state of four conformers of CPB predicted with the B3LYP/aug-cc-pVTZ method

mode	CPB-1				CPB-2			
	A'/*A*"	*B*'/*B*"	*C*'/*C*"	*a*-/*b*-/*c*-ratio	*A*'/*A*"	*B*'/*B*"	*C*'/*C*"	*a*-/*b*-/*c*-ratio
	A" / cm^{-1}	*B*" / cm^{-1}	*C*" / cm^{-1}		*A*" / cm^{-1}	*B*" / cm^{-1}	*C*" / cm^{-1}	
	0.10551	0.04332	0.03535		0.12364	0.03811	0.03293	
ν_{1}	0.99968	0.99979	0.99977	36/16/48	0.99964	0.99990	0.99985	61/0/39
ν_{2}	0.99954	0.99977	0.99963	42/1/56	0.99909	0.99995	0.99964	54/7/39
ν_{3}	0.99970	0.99979	0.99977	3/34/63	0.99967	0.99987	0.99985	1/56/43
ν_{4}	0.99956	1.00032	1.00017	48/50/2	0.99714	1.00118	0.99991	86/3/11
ν_{5}	1.00003	0.99986	0.99997	11/1/88	0.99908	1.00050	1.00012	5/10/85
ν_{6}	0.99984	1.00000	1.00003	32/68/1	0.99918	1.00029	1.00000	15/84/0
ν_{7}	0.99928	1.00145	1.00079	3/0/97	1.00272	0.99879	1.00009	0/5/94
ν_{8}	0.99787	0.99970	0.99929	1/62/37	0.99829	0.99945	0.99918	13/53/34
ν_{9}	1.00033	1.00005	1.00031	15/0/85	1.00146	1.00003	1.00018	1/1/98
ν_{10}	1.00094	0.99993	1.00008	4/1/95	1.00106	0.99950	1.00024	23/14/62
ν_{11}	0.99986	0.99970	0.99989	17/69/13	1.00061	0.99950	0.99994	26/65/10
ν_{12}	0.99883	0.99931	0.99898	32/64/5	0.99825	0.99961	0.99924	7/82/11
ν_{13}	1.00109	0.99748	0.99830	71/26/3	1.00015	1.00079	1.00061	40/57/3
ν_{14}	0.99999	0.99831	0.99822	81/18/1	0.99961	0.99753	0.99778	90/0/10
ν_{15}	1.00033	0.99940	0.99989	57/16/27	1.00154	0.99822	0.99888	94/6/0
ν_{16}	1.00064	0.99905	0.99918	57/40/3	1.00185	0.99822	0.99879	76/20/4
ν_{17}	0.99867	0.99861	0.99859	51/48/2	1.00032	0.99861	0.99879	33/67/0
ν_{18}	0.99886	0.99894	0.99836	77/2/20	0.99647	1.00003	0.99882	73/4/23
ν_{19}	1.00026	1.00016	1.00031	63/13/24	1.00121	0.99997	1.00052	24/29/47
ν_{20}	0.99930	0.99831	0.99833	27/20/53	0.99878	0.99861	0.99866	10/44/46
ν_{21}	1.00072	0.99898	0.99952	69/9/22	1.00094	0.99887	0.99945	34/31/35
ν_{22}	0.99713	1.00025	0.99887	99/1/0	0.99855	0.99871	0.99848	53/12/35
ν_{23}	1.00154	0.99723	0.99782	65/7/28	1.00027	0.99961	0.99854	66/22/12
ν_{24}	0.99727	1.00000	0.99955	33/14/54	0.99995	0.99693	0.99921	67/12/22
ν_{25}	1.00044	0.99827	0.99904	27/46/27	0.99841	1.00079	1.00012	4/75/21
ν_{26}	0.99621	1.00081	1.00031	93/1/6	0.99967	0.99801	1.00018	77/19/4
ν_{27}	0.99998	0.99732	0.99805	54/9/37	0.99550	1.00055	0.99800	94/2/4
ν_{28}	0.99973	0.99898	0.99884	40/0/60	1.00125	0.99748	0.99860	7/36/57
ν_{29}	1.00187	0.99582	0.99703	46/51/4	1.00153	0.99738	0.99800	94/4/2
ν_{30}	0.99929	1.00187	1.00031	36/30/34	0.99753	1.00210	1.00033	55/6/38
ν_{31}	1.00069	0.99857	0.99941	92/8/0	0.99450	1.00126	0.99854	84/12/4
ν_{32}	1.00008	0.99859	0.99861	88/11/0	0.99973	0.99848	0.99787	27/66/6
ν_{33}	0.99907	0.99979	0.99963	40/39/21	1.00310	0.99819	1.00052	60/9/31
ν_{34}	1.00299	0.99938	0.99915	22/8/70	1.00290	0.99987	0.99924	36/33/31
ν_{35}	1.00178	1.00072	1.00071	61/26/14	1.00154	1.00084	1.00140	6/0/94
ν_{36}	1.00109	1.00196	0.99969	11/82/7	1.00139	1.00123	0.99976	0/44/56

mode	CPB-3				CPB-4			
	A'/A''	B'/B''	C'/C''	*a*-/*b*-/*c*-ratio	A'/A''	B'/B''	C'/C''	*a*-/*b*-/*c*-ratio
	A'' / cm^{-1}	B'' / cm^{-1}	C'' / cm^{-1}		A'' / cm^{-1}	B'' / cm^{-1}	C'' / cm^{-1}	
	0.16556	0.03428	0.03124		0.14653	0.03617	0.03218	
ν_1	0.99912	0.99991	0.99978	4/94/2	0.99923	0.99992	0.99978	0/99/1
ν_2	0.99924	0.99980	0.99974	22/78/0	1.00164	0.99837	0.99863	0/95/5
ν_3	0.99920	0.99988	0.99971	71/29/0	0.99904	0.99997	0.99981	99/1/1
ν_4	0.99757	1.00064	0.99984	71/11/17	0.99928	1.00030	1.00016	56/38/6
ν_5	0.99938	1.00000	0.99990	8/3/89	1.00002	0.99975	0.99975	2/2/96
ν_6	0.99891	1.00015	0.99990	38/61/1	0.99967	0.99997	0.99994	36/63/1
ν_7	1.00265	0.99930	1.00051	2/17/81	0.99587	1.00202	1.00146	13/0/87
ν_8	1.00027	0.99860	0.99862	90/8/2	0.99978	0.99867	0.99885	80/19/1
ν_9	1.00095	1.00018	0.99997	1/7/92	1.00000	0.99997	1.00025	4/21/75
ν_{10}	1.00040	0.99994	1.00026	0/53/47	1.00102	1.00019	1.00012	3/20/77
ν_{11}	0.99992	0.99994	0.99987	96/4/0	1.00015	0.99983	0.99978	99/1/0
ν_{12}	0.99764	0.99985	0.99942	4/96/1	0.99822	0.99956	0.99916	4/96/0
ν_{13}	0.99908	0.99985	0.99984	0/99/0	1.00272	0.99809	0.99984	40/55/4
ν_{14}	0.99982	0.99892	0.99866	94/2/4	0.99855	0.99925	0.99838	94/5/1
ν_{15}	1.00016	0.99918	0.99891	95/0/4	0.99977	0.99942	0.99879	93/0/7
ν_{16}	1.00114	0.99936	0.99910	71/29/1	1.00061	0.99920	0.99891	4/92/4
ν_{17}	1.00100	1.00012	0.99901	91/3/7	1.00114	0.99931	0.99891	86/4/10
ν_{18}	0.99738	0.99886	0.99946	29/3/69	0.99895	0.99967	0.99953	16/5/79
ν_{19}	0.99975	1.00096	0.99990	14/5/82	0.99985	1.00025	0.99984	4/8/88
ν_{20}	1.00085	0.99828	0.99914	42/31/26	1.00093	0.99795	0.99876	21/21/58
ν_{21}	0.99610	0.99974	1.00010	2/1/97	0.99663	0.99959	0.99994	0/1/99
ν_{22}	1.00116	0.99866	0.99827	86/10/3	1.00040	0.99862	0.99823	93/0/7
ν_{23}	0.99940	0.99816	0.99837	14/0/86	0.99865	0.99917	0.99835	64/7/30
ν_{24}	0.99859	0.99851	0.99907	73/3/24	0.99661	0.99939	0.99963	12/0/88
ν_{25}	0.99931	0.99956	0.99978	26/18/56	0.99879	0.99898	0.99941	45/0/55
ν_{26}	0.99871	0.99810	0.99907	89/6/4	0.99885	0.99795	0.99786	96/1/3
ν_{27}	1.00133	0.99851	0.99952	0/5/95	0.99677	0.99981	1.00028	43/14/43
ν_{28}	0.99604	1.00006	0.99866	18/67/14	0.99933	0.99900	0.99960	64/2/34
ν_{29}	0.99859	0.99883	0.99834	68/0/31	1.00266	0.99801	0.99798	35/33/31
ν_{30}	1.00258	0.99842	0.99891	92/3/5	0.99894	0.99936	0.99888	80/18/2
ν_{31}	0.99544	1.00085	0.99875	95/0/5	0.99793	1.00077	0.99988	99/1/0
ν_{32}	0.99953	0.99912	0.99914	44/56/0	0.99911	0.99964	0.99922	77/22/1
ν_{33}	0.99892	0.99927	1.00000	66/34/0	0.99765	0.99939	0.99897	94/5/1
ν_{34}	1.00233	0.99889	0.99882	32/1/67	1.00059	0.99895	0.99879	34/0/66
ν_{35}	1.00828	1.00058	1.00138	68/17/15	1.00261	1.00100	1.00137	82/3/16
ν_{36}	0.99138	1.00079	0.99994	2/23/75	0.99661	1.00122	1.00034	20/73/7

Table S11. Rotational parameters and *a*-/*b*-/*c*-type ratios for each vibrational state of two conformers of CMP predicted with the B3LYP/aug-cc-pVTZ method

mode	*trans*-CMP				*cis*-CMP			
	A'/*A*"	*B*'/*B*"	*C*'/*C*"	*a*-/*b*-/*c*-ratio	*A*'/*A*"	*B*'/*B*"	*C*'/*C*"	*a*-/*b*-/*c*-ratio
	A" / cm^{-1}	*B*" / cm^{-1}	*C*" / cm^{-1}		*A*" / cm^{-1}	*B*" / cm^{-1}	*C*" / cm^{-1}	
	0.13007	0.07183	0.06481		0.13025	0.07316	0.06260	
ν_1	0.99915	0.99997	0.99958	4/79/17	0.99948	0.99979	0.99971	9/30/61
ν_2	0.99768	1.00088	1.00006	67/13/20	0.99863	1.00033	1.00003	0/0/100
ν_3	0.99938	0.99979	0.99952	91/0/9	1.00069	0.99979	1.00008	78/20/2
ν_4	1.00075	0.99965	0.99998	4/73/23	0.99960	0.99969	0.99971	90/10/0
ν_5	1.00141	0.99893	0.99951	83/7/10	0.99995	1.00008	1.00010	13/83/4
ν_6	1.00018	0.99957	0.99971	18/76/5	1.00005	0.99988	0.99995	2/72/26
ν_7	1.00544	0.99481	0.99741	34/65/1	1.00021	0.99785	0.99829	85/12/3
ν_8	1.00114	1.00045	0.99980	0/70/29	1.00186	1.00030	1.00021	5/30/65
ν_9	1.00056	1.00035	0.99995	28/72/1	0.99992	1.00023	1.00022	70/0/30
ν_{10}	1.00141	0.99879	0.99927	94/0/6	1.00104	0.99895	0.99971	51/14/35
ν_{11}	0.99646	0.99971	0.99963	7/62/31	0.99808	0.99933	0.99909	13/86/0
ν_{12}	1.00184	0.99904	0.99995	1/57/42	1.00074	0.99940	1.00019	10/89/1
ν_{13}	0.99220	1.00422	1.00046	35/20/44	1.00108	0.99692	0.99994	64/35/1
ν_{14}	0.99173	1.00216	1.00034	71/12/17	0.99795	0.99958	0.99906	74/26/0
ν_{15}	0.99622	1.00150	1.00039	76/12/12	1.00193	0.99966	1.00265	24/55/20
ν_{16}	1.00104	0.99884	0.99931	76/2/21	0.99929	0.99896	0.99751	2/83/15
ν_{17}	0.99254	1.00409	1.00052	3/9/87	0.99945	0.99923	0.99965	60/34/7
ν_{18}	0.99743	1.00031	0.99968	15/19/66	0.99800	1.00038	0.99984	13/68/19
ν_{19}	1.00158	0.99663	0.99906	64/21/15	0.99717	0.99743	0.99768	37/3/60
ν_{20}	0.99599	0.99884	0.99764	72/12/16	0.99719	0.99835	0.99738	58/38/4
ν_{21}	1.00327	0.99759	0.99840	11/56/34	1.00068	0.99854	0.99883	20/69/12
ν_{22}	0.99317	1.00274	1.00056	91/8/1	0.99419	1.00154	0.99935	80/20/0
ν_{23}	1.00178	0.99787	0.99773	82/17/1	1.00596	0.99543	0.99843	61/38/1
ν_{24}	0.99483	1.00455	0.99907	52/18/30	0.99992	0.99914	0.99963	51/48/2
ν_{25}	0.99862	0.99986	0.99960	71/23/7	0.99913	0.99937	0.99861	78/20/2
ν_{26}	0.99604	1.00014	1.00079	62/32/6	0.99830	1.00044	1.00070	98/2/0
ν_{27}	0.99687	1.00035	0.99810	2/97/1	0.99985	0.99872	0.99765	30/0/69
ν_{28}	0.99808	0.99981	0.99836	86/0/13	0.99932	0.99902	0.99827	0/4/96
ν_{29}	1.00484	0.99623	0.99634	61/33/5	0.99813	1.00053	0.99920	0/54/46
ν_{30}	1.00788	0.99722	0.99765	28/41/31	1.00002	1.00220	1.00037	98/2/1

Table S12. Comparison of Observed Wavenumbers (cm^{-1}) and Relative Intensities of Bands in Group B with Scaled Harmonic and Anharmonic Vibrational Wavenumbers and Infrared Intensities of Two Conformers of $(C_2H_3)CCl(CH_3)O$, *trans*-CMP and *cis*-CMP, Predicted with the B3LYP/aug-cc-pVTZ Method

experiments		*trans*-CMP				*cis*-CMP			
		mode	scaled harmonic		anharm.	mode	scaled harmonic		anharm.
		ν_7	1607[a]	(6)[b]	1614	ν_7	1646	(12)	1654
		ν_8	1458	(6)	1450	ν_8	1452	(5)	1443
1423	(29)[c]	ν_9	1446	(3)	1433	ν_9	1429	(4)	1421
1360	(100)	ν_{10}	1400	(23)	1394	ν_{10}	1405	(9)	1401
		ν_{11}	1363	(2)	1357	ν_{11}	1355	(5)	1361
		ν_{12}	1290	(3)	1290	ν_{12}	1290	(3)	1293
1144	(65)	ν_{13}	1184	(43)	1148	ν_{13}	1162	(38)	1127
		ν_{14}	1082	(0)	1053				
1080	(35)	ν_{15}	1065	(63)	1059	ν_{14}	1052	(55)	1046
						ν_{15}	1023	(9)	1023
		ν_{16}	988	(14)	984	ν_{16}	995	(19)	974
		ν_{17}	977	(5)	952	ν_{17}	967	(6)	962
		ν_{18}	955	(40)	953	ν_{18}	962	(35)	955
		ν_{19}	921	(29)	921	ν_{19}	880	(4)	848

[a]Scaling equation $y = 0.971\ x + 10.4$, in which y is the scaled harmonic vibrational wavenumber and x is the harmonic vibrational wavenumber calculated with the B3LYP/aug-cc-pVTZ method. [b]Harmonic IR intensities (in km mol^{-1}) are listed in parentheses. [c]Percentage integrated intensities relative to that of the most intense band near 1360 cm^{-1} (ν_{10}).

Table S13. Comparison of Experimental Vibrational Wavenumbers (cm^{-1}) and Relative IR Intensities of *s-trans*-and *s-cis*-$C_2H_3C(O)Cl$ with Scaled Harmonic Vibrational Wavenumbers and Relative IR intensities Predicted with the B3LYP/aug-cc-pVTZ Method

mode	sym.	*s-trans*			*s-cis*		
		expt.[a]	harmonic[b]	intensity[c]	expt.[a]	harmonic[b]	intensity[c]
ν_1	a'	3122	3130	0.4		3127	0.3
ν_2	a'	3069	3063	1.5	3087	3098	0.6
ν_3	a'	3043	3043	1.8		3039	2.4
ν_4	a'	1789	1788	361	1755	1801	262
ν_5	a'	1628	1645	2.5	1620	1629	36
ν_6	a'	1397	1409	26		1405	60
ν_7	a'	1285	1290	3.0		1294	1.9
ν_8	a'	1152	1134	118		1075	4.3
ν_9	a'	939	927	113	979	954	300
ν_{10}	a'	609	590	65	708	697	101
ν_{11}	a'	494	489	28	446	430	22
ν_{12}	a'	445	421	25	386	383	26
ν_{13}	a'	257	255	<0.1		252	1.0
ν_{14}	a"	981/977	1009	9.2	983	1007	27
ν_{15}	a"	981/977	998	39	975	1003	19
ν_{16}	a"	757	767	14	744	753	16
ν_{17}	a"	452	455	<0.1	485	474	<0.1
ν_{18}	a"	108	122	0.8	95	90	<0.1

[a] Obtained from J. Chem. Phys. **87**, 6303 (1987)[2] & J. Chem. Phys. **139**, 084320 (2013).[3] [b] Harmonic vibrational wavenumbers x under 2000 cm^{-1} are scaled according to y = 0.971 x + 10.4, and those larger than 2000 cm^{-1} according to y = 0.955 x + 30.7. [c] In km mol^{-1}

Table S14. Scaled Harmonic Vibrational Wavenumbers and IR Intensities of Six Conformers of $C_2H_3C(OH)Cl(CH_3)$ Predicted with the B3LYP/aug-cc-pVTZ Method

mode	$C_2H_3C(OH)Cl(CH_3)$-1		$C_2H_3C(OH)Cl(CH_3)$-2		$C_2H_3C(OH)Cl(CH_3)$-3	
	harmonic[a]	intensity[b]	harmonic[a]	intensity[b]	harmonic[a]	intensity[b]
ν_1	3638	36	3659	49	3644	42
ν_2	3112	4.7	3124	2.7	3115	5.7
ν_3	3063	1.1	3063	2.5	3040	2.8
ν_4	3033	3.8	3040	4.7	3037	4.6
ν_5	3030	6.2	3018	7.4	3024	7.2
ν_6	3009	7.5	2993	13	3014	6.6
ν_7	2941	8.4	2930	10	2946	8.6
ν_8	1657	2.7	1664	0.5	1664	2.7
ν_9	1456	1.8	1461	5.0	1460	1.3
ν_{10}	1451	1.1	1451	5.6	1459	3.0
ν_{11}	1420	12	1424	25.4	1423	12
ν_{12}	1385	13	1381	13	1396	34
ν_{13}	1333	46	1335	23	1357	38
ν_{14}	1293	20	1306	2.8	1300	14
ν_{15}	1242	61	1214	138	1181	83
ν_{16}	1098	93	1095	12	1141	63
ν_{17}	1042	43	1056	69	1072	59
ν_{18}	1025	27	1029	24	1006	11
ν_{19}	1012	11	1008	13	1002	45
ν_{20}	960	19	962	39	952	45
ν_{21}	952	38	951	47	943	13
ν_{22}	779	49	783	50	784	79
ν_{23}	703	48	700	37	718	29
ν_{24}	629	47	605	26	561	40
ν_{25}	479	5.6	487	42	456	7.8
ν_{26}	432	77	411	11	440	24
ν_{27}	400	5.6	391	27	398	59
ν_{28}	353	69	341	90	344	67
ν_{29}	309	3.1	311	3.1	315	11
ν_{30}	279	8.5	283	5.2	277	9.2
ν_{31}	272	1.2	270	5.3	269	1.5
ν_{32}	251	3.4	253	14	254	7.6
ν_{33}	115	0.7	108	1.4	108	0.3

mode	$C_2H_3C(OH)Cl(CH_3)$-4		$C_2H_3C(OH)Cl(CH_3)$-5		$C_2H_3C(OH)Cl(CH_3)$-6	
	harmonic[a]	intensity[b]	harmonic[a]	intensity[b]	harmonic[a]	intensity[b]
ν_1	3642	41	3643	43	3645	38
ν_2	3121	2.8	3114	6.5	3120	3.6
ν_3	3040	5.3	3059	0.7	3042	4.5
ν_4	3032	6.6	3038	4.7	3028	6.3
ν_5	3017	8.4	3024	6.3	3023	5.8
ν_6	3006	7.2	2998	12	2990	15
ν_7	2940	11	2937	8.5	2929	12
ν_8	1669	4.4	1669	1.5	1677	3.1
ν_9	1456	1.3	1469	0.8	1460	2.3
ν_{10}	1451	2.1	1453	3.9	1450	2.8
ν_{11}	1416	25	1424	17	1419	16
ν_{12}	1385	15	1385	23	1380	11
ν_{13}	1330	65	1353	38	1337	54
ν_{14}	1298	4.6	1303	2.7	1297	6.1
ν_{15}	1184	43	1194	28	1149	142
ν_{16}	1128	119	1118	167	1116	53
ν_{17}	1050	34	1065	55	1067	35
ν_{18}	1012	30	1019	15	1023	8.9
ν_{19}	1006	17	1011	8.5	1000	8.5
ν_{20}	971	40	952	41	965	42.1
ν_{21}	944	22	939	40	940	45
ν_{22}	812	61	779	56	809	45
ν_{23}	701	13	714	17	691	8.8
ν_{24}	581	31	555	44	584	28
ν_{25}	475	38	462	43	469	33
ν_{26}	440	52	441	15	436	38
ν_{27}	399	10	409	31	400	11
ν_{28}	361	52	336	58	347	0.9
ν_{29}	353	4.8	313	34	331	83
ν_{30}	297	4.0	285	5.4	294	3.3
ν_{31}	255	1.7	275	1.0	274	0.7
ν_{32}	245	0.8	256	5.3	238	0.8
ν_{33}	97	0.2	106	0.2	85	0.5

[a] Harmonic vibrational wavenumbers x under 2000 cm^{-1} are scaled according to $y = 0.971\ x + 10.4$, and those larger than 2000 cm^{-1} according to $y = 0.955\ x + 30.7$. [b] In km mol^{-1}.

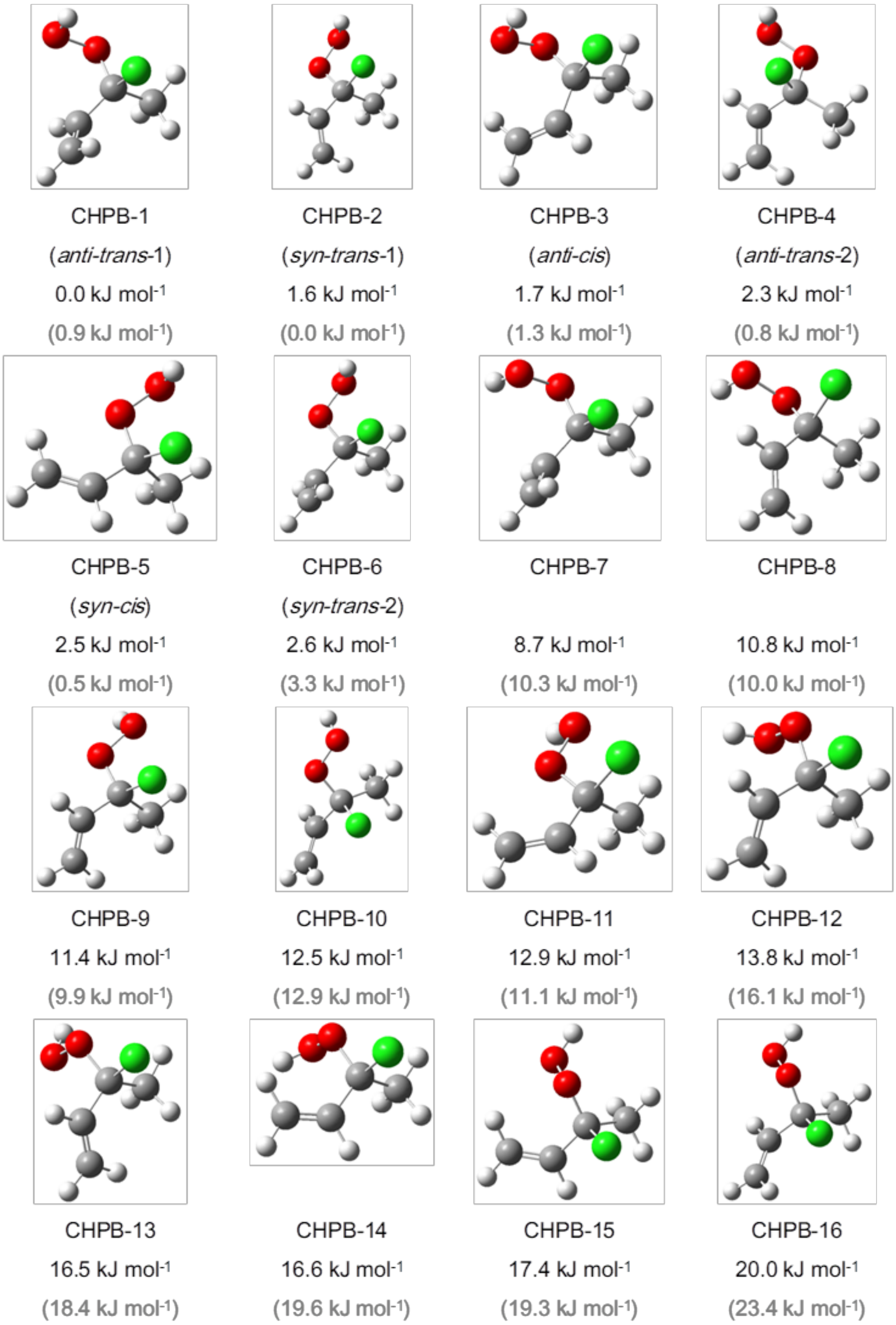

Figure S1. Geometries of sixteen conformers of $(C_2H_3)C(CH_3)(Cl)OOH$ (CHPB) calculated with the B3LYP/aug-cc-pVTZ method. Energies were calculated with the CCSD(T)/aug-cc-pVTZ//B3LYP/aug-cc-pVTZ method; those in parentheses were calculated with the B3LYP/aug-cc-pVTZ method.

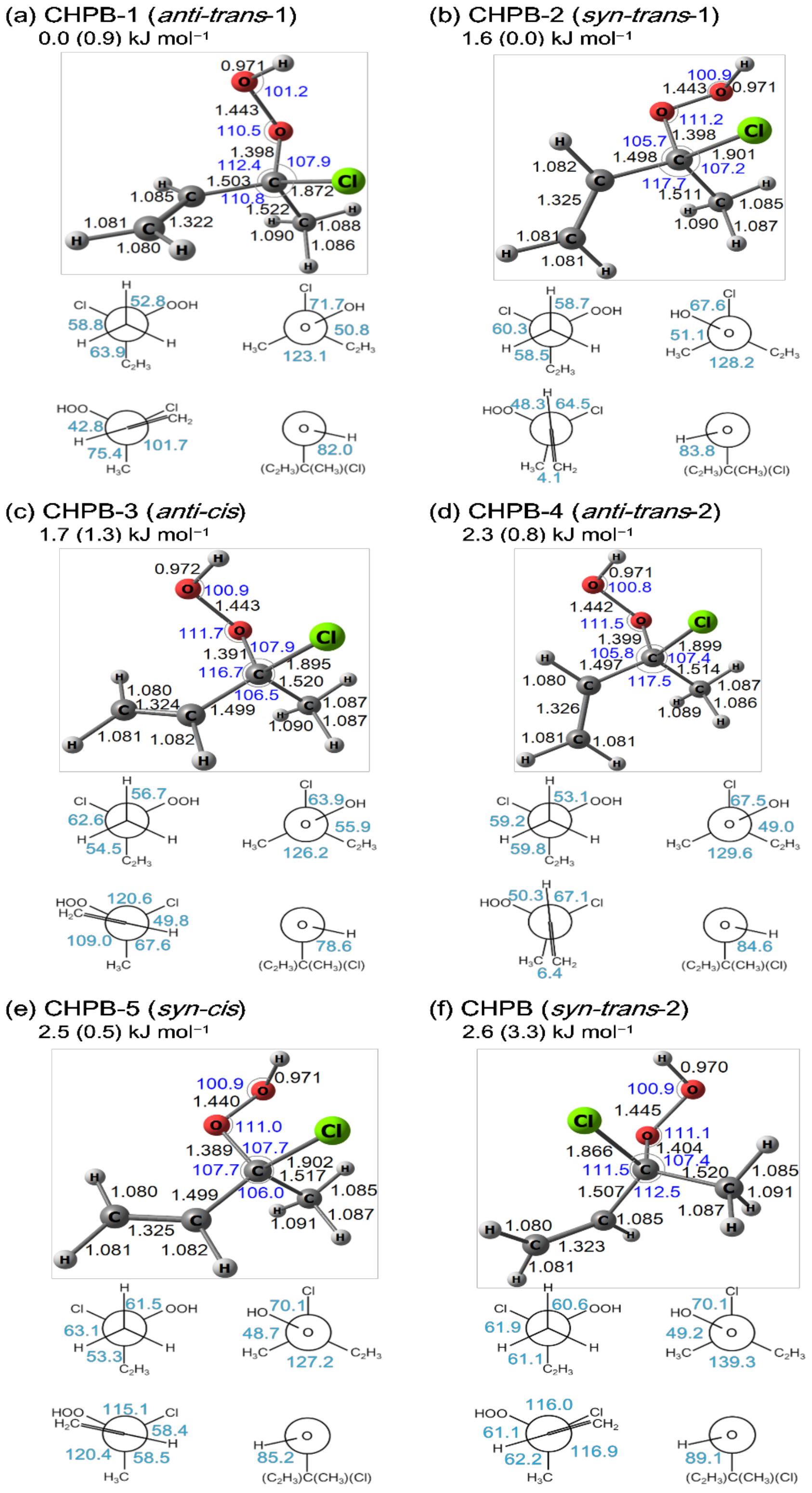

Figure S2. Geometries of six lowest-energy conformers of $(C_2H_3)C(CH_3)(Cl)OOH$ (CHPB) calculated with the B3LYP/aug-cc-pVTZ method. (a) CHPB-1, (b) CHPB-2, (c) CHPB-3, (d) CHPB-4, (e) CHPB-5, and (f) CHPB-6. Bond lengths are in Å and bond angles in degrees. Energies were calculated with the CCSD(T)/aug-cc-pVTZ//B3LYP/aug-cc-pVTZ method; those in parentheses were calculated with the B3LYP/aug-cc-pVTZ method.

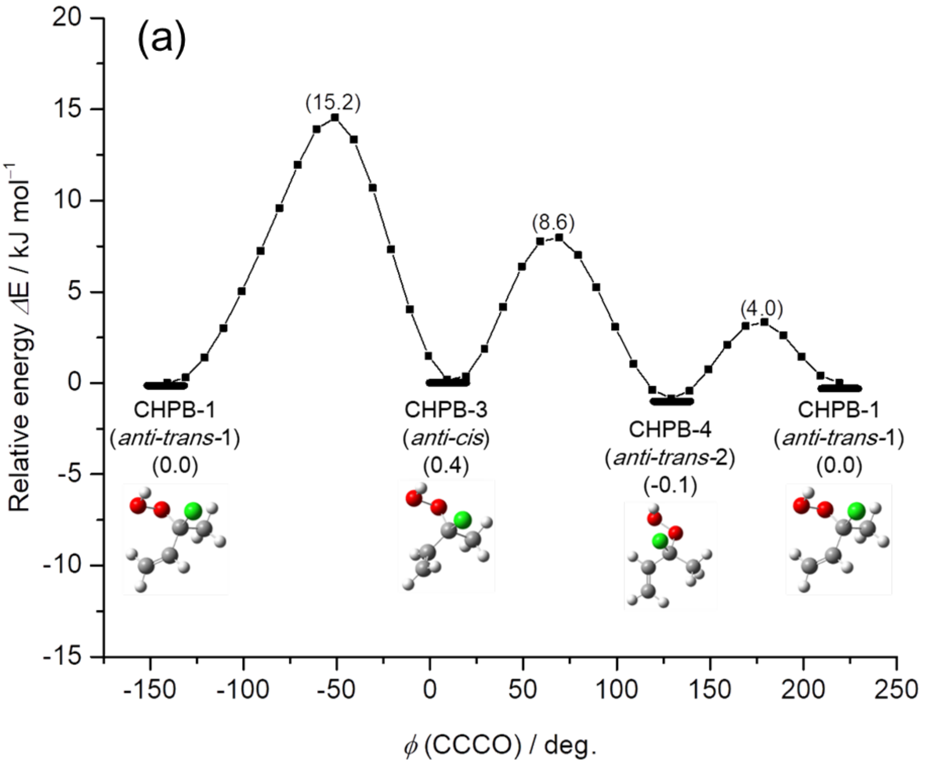
(a)
Relative energy ΔE / kJ mol⁻¹
(15.2)
(8.6)
(4.0)
CHPB-1
(anti-trans-1)
(0.0)
CHPB-3
(anti-cis)
(0.4)
CHPB-4
(anti-trans-2)
(-0.1)
CHPB-1
(anti-trans-1)
(0.0)
φ (CCCO) / deg.

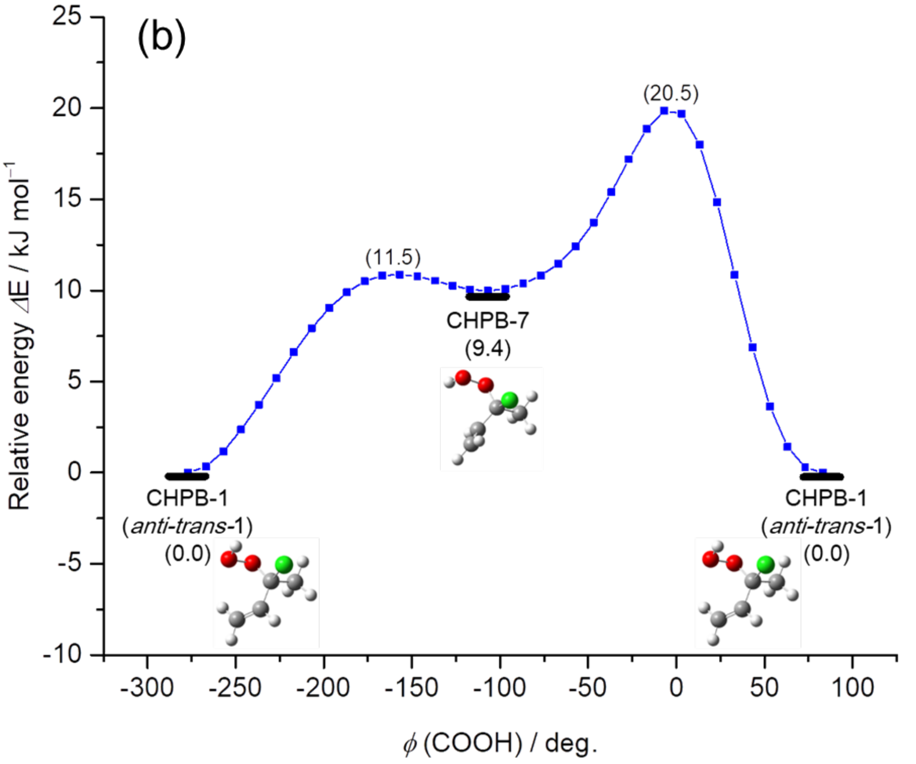
(b)
Relative energy ΔE / kJ mol⁻¹
(11.5)
(20.5)
CHPB-7
(9.4)
CHPB-1
(anti-trans-1)
(0.0)
CHPB-1
(anti-trans-1)
(0.0)
φ (COOH) / deg.

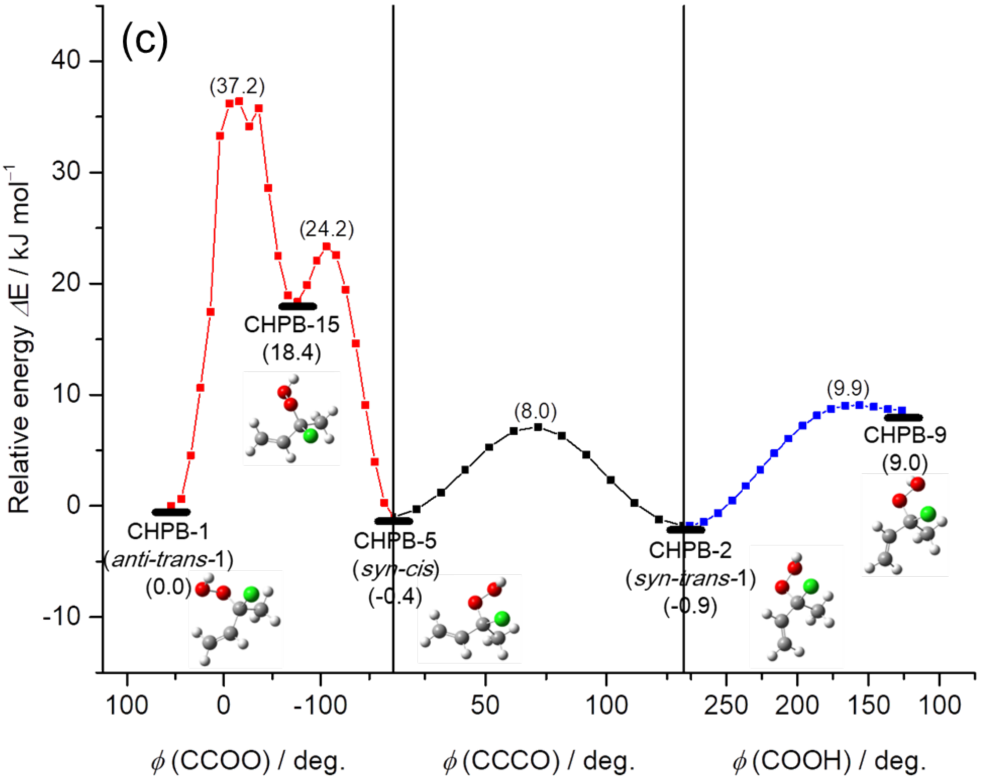
(c)
Relative energy ΔE / kJ mol⁻¹
40
30
20
10
0
-10
(37.2)
(24.2)
CHPB-15
(18.4)
(8.0)
(9.9)
CHPB-9
(9.0)
CHPB-1
(anti-trans-1)
(0.0)
CHPB-5
(syn-cis)
(-0.4)
CHPB-2
(syn-trans-1)
(-0.9)
100
0
-100
50
100
250
200
150
100
ϕ (CCOO) / deg.
ϕ (CCCO) / deg.
ϕ (COOH) / deg.

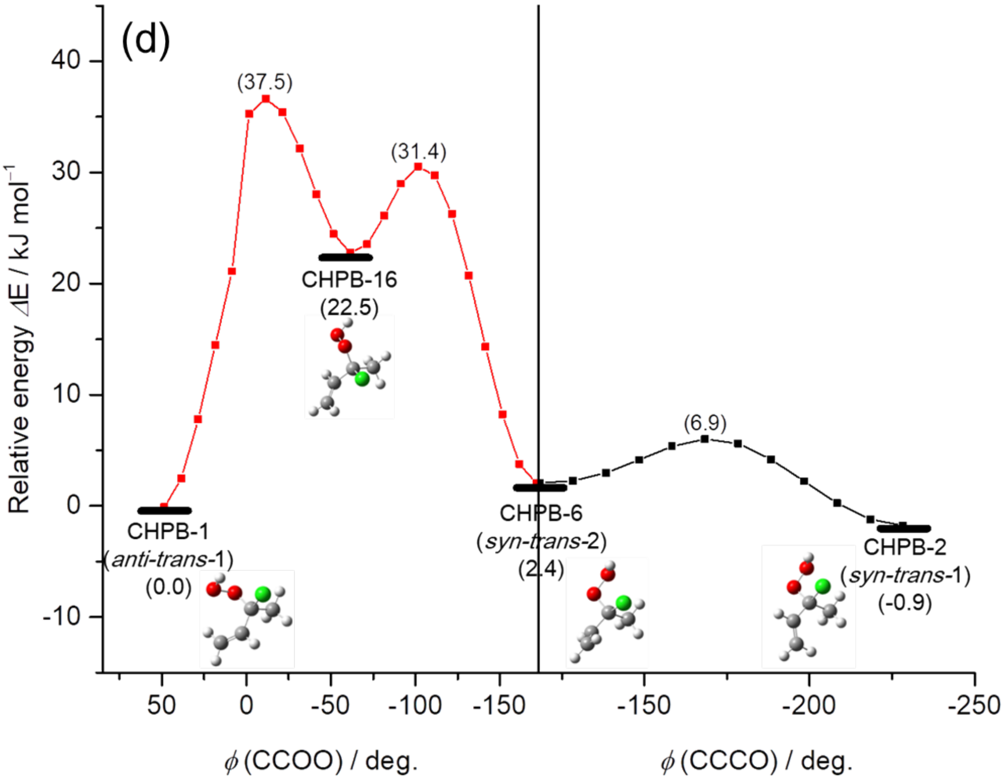
(d)
Relative energy ΔE / kJ mol⁻¹
40
30
20
10
0
-10
(37.5)
(31.4)
CHPB-16
(22.5)
(6.9)
CHPB-1
(anti-trans-1)
(0.0)
CHPB-6
(syn-trans-2)
(2.4)
CHPB-2
(syn-trans-1)
(-0.9)
50
0
-50
-100
-150
-150
-200
-250
ϕ (CCOO) / deg.
ϕ (CCCO) / deg.

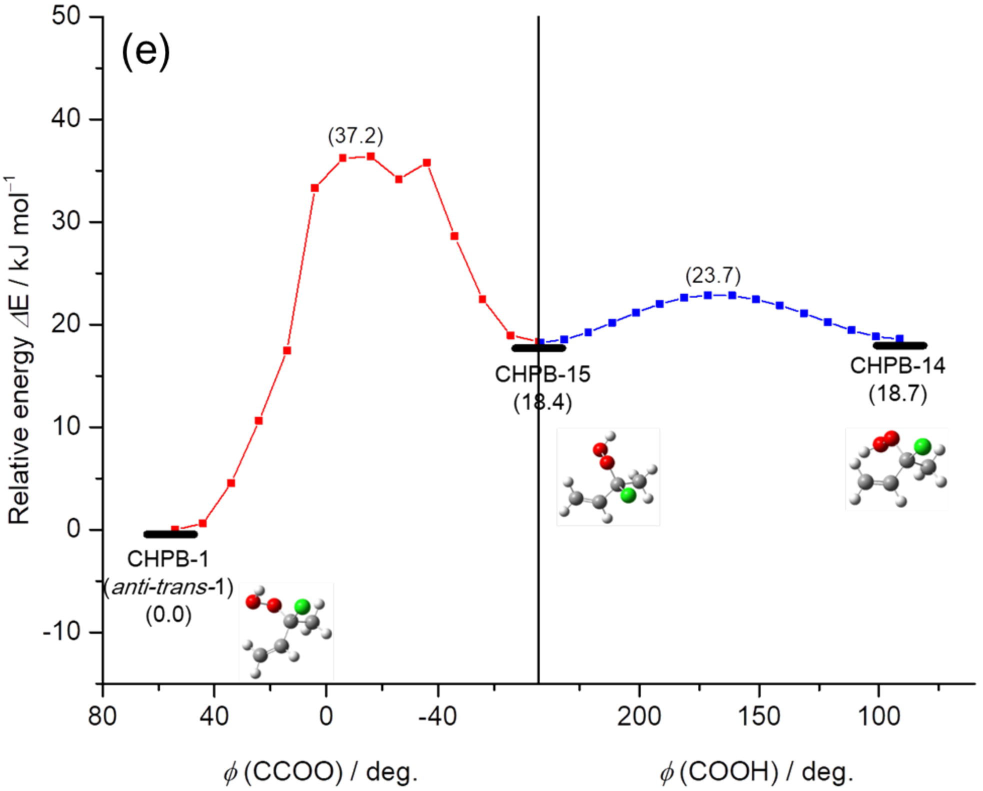
(e)
Relative energy ΔE / kJ mol⁻¹
(37.2)
(23.7)
CHPB-15
(18.4)
CHPB-14
(18.7)
CHPB-1
(anti-trans-1)
(0.0)
φ (CCOO) / deg.
φ (COOH) / deg.

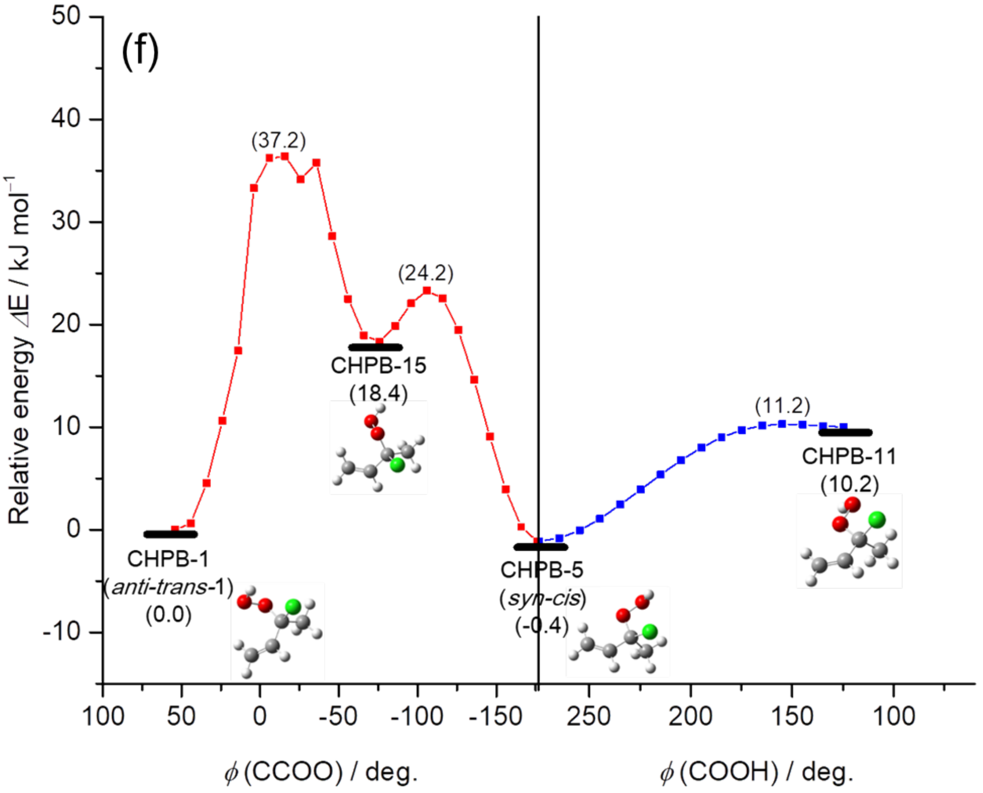
(f)
Relative energy ΔE / kJ mol⁻¹
(37.2)
(24.2)
CHPB-15
(18.4)
(11.2)
CHPB-11
(10.2)
CHPB-1
(anti-trans-1)
(0.0)
CHPB-5
(syn-cis)
(-0.4)
φ (CCOO) / deg.
φ (COOH) / deg.

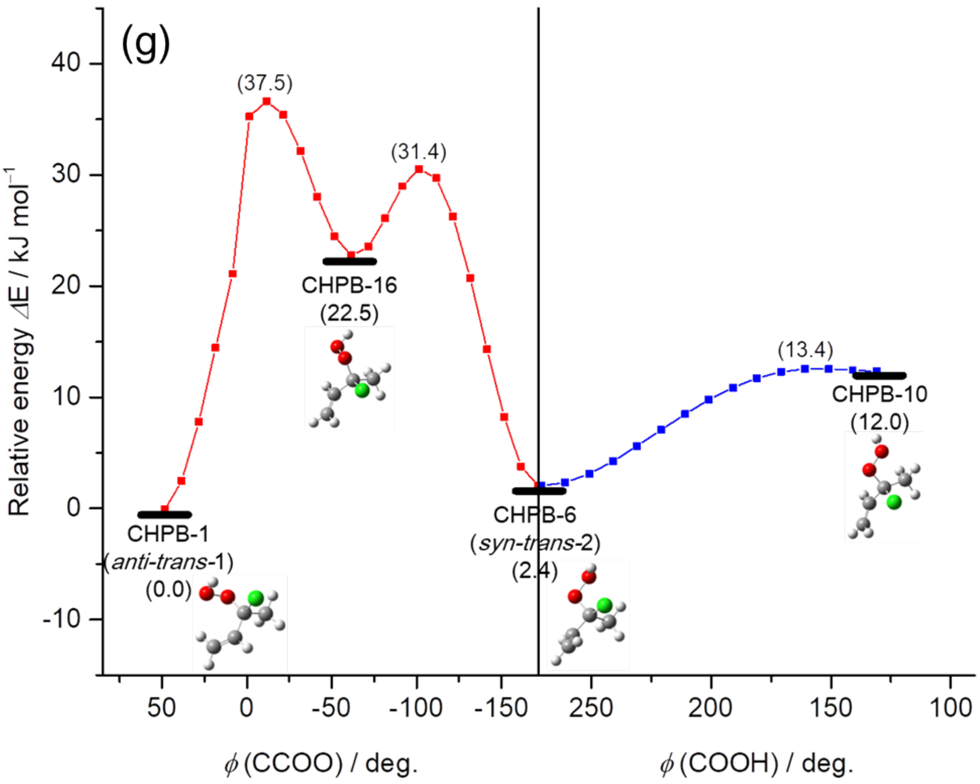

Figure S3. Potential energies for the conformation interconversion started from *anti-trans*1-CHPB (CHPB-1) calculated with the B3LYP/aug-cc-pVTZ method. (a) to CHPB-3 and CHPB-4. (b) to CHPB-7. (c) to CHPB-2, CHPB-5, CHPB-9, and CHPB-15. (d) to CHPB-2, CHPB-6, and CHPB-16. (e) to CHPB-14 and CHPB-15. (f) to CHPB-5, CHPB-11, and CHPB-15. (g) to CHPB-6, CHPB-10, and CHPB-16. The scans for dihedral angles Φ(CCCO), Φ(CCOO), and Φ(COOH) are presented in black, red, and blue, respectively. Energies were calculated with the B3LYP/aug-cc-pVTZ method; the energy of CHPB-1 is set to zero.

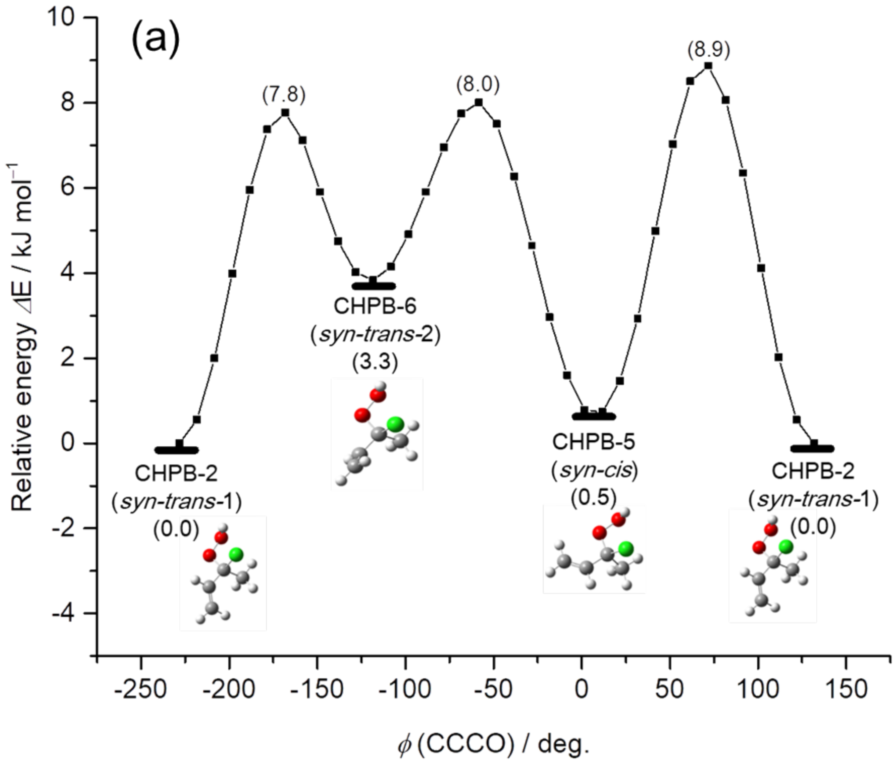
(a)
(7.8)
(8.0)
(8.9)
CHPB-6
(syn-trans-2)
(3.3)
CHPB-5
(syn-cis)
(0.5)
CHPB-2
(syn-trans-1)
(0.0)
CHPB-2
(syn-trans-1)
(0.0)
Relative energy ΔE / kJ mol⁻¹
ϕ (CCCO) / deg.

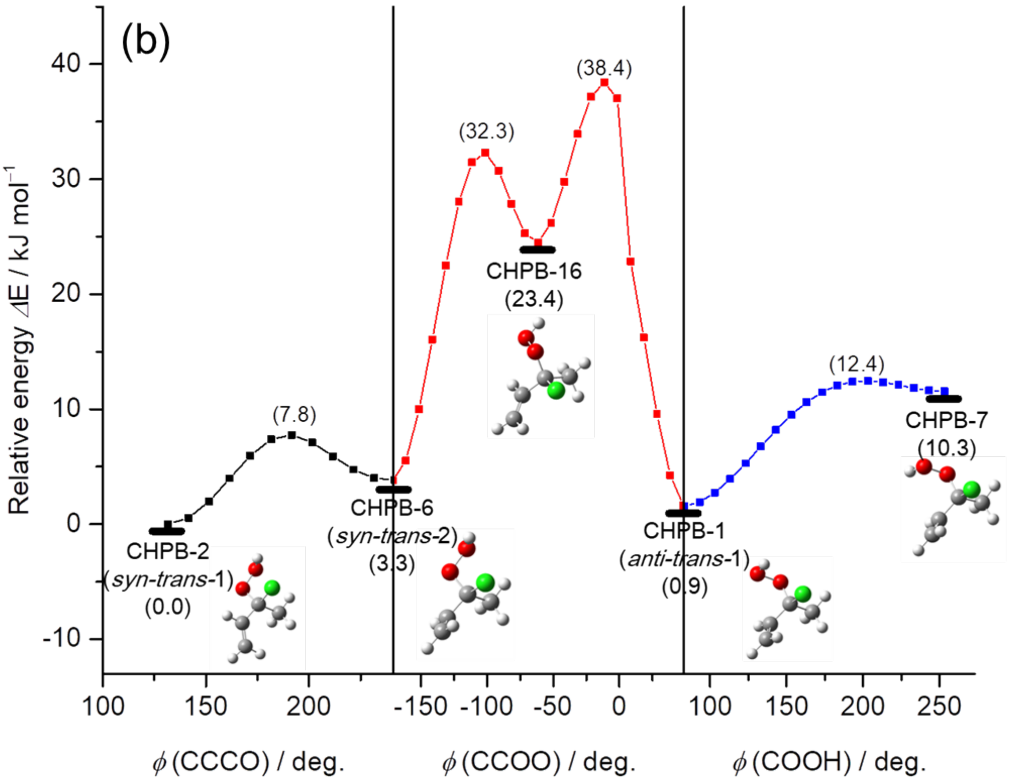
(b)
(7.8)
(32.3)
(38.4)
(12.4)
CHPB-16
(23.4)
CHPB-7
(10.3)
CHPB-2
(syn-trans-1)
(0.0)
CHPB-6
(syn-trans-2)
(3.3)
CHPB-1
(anti-trans-1)
(0.9)
Relative energy ΔE / kJ mol⁻¹
ϕ (CCCO) / deg.
ϕ (CCOO) / deg.
ϕ (COOH) / deg.

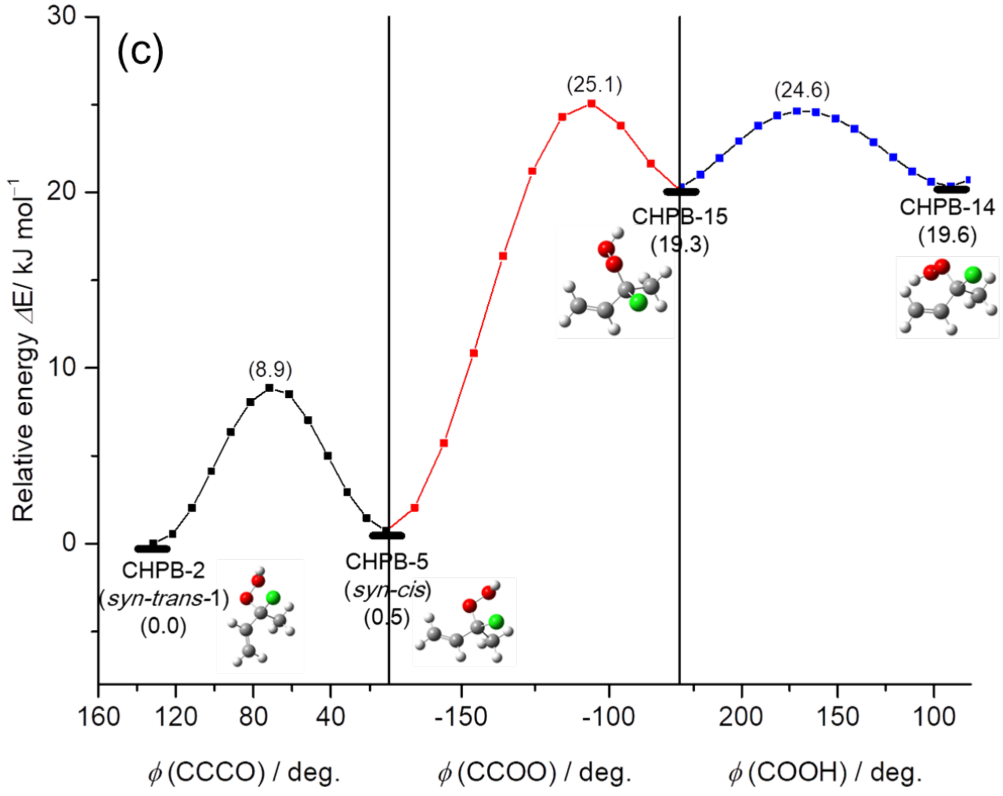
(c)
Relative energy ΔE/ kJ mol⁻¹
30
20
10
0
(8.9)
(25.1)
(24.6)
CHPB-2
(syn-trans-1)
(0.0)
CHPB-5
(syn-cis)
(0.5)
CHPB-15
(19.3)
CHPB-14
(19.6)
160
120
80
40
-150
-100
200
150
100
ϕ (CCCO) / deg.
ϕ (CCOO) / deg.
ϕ (COOH) / deg.

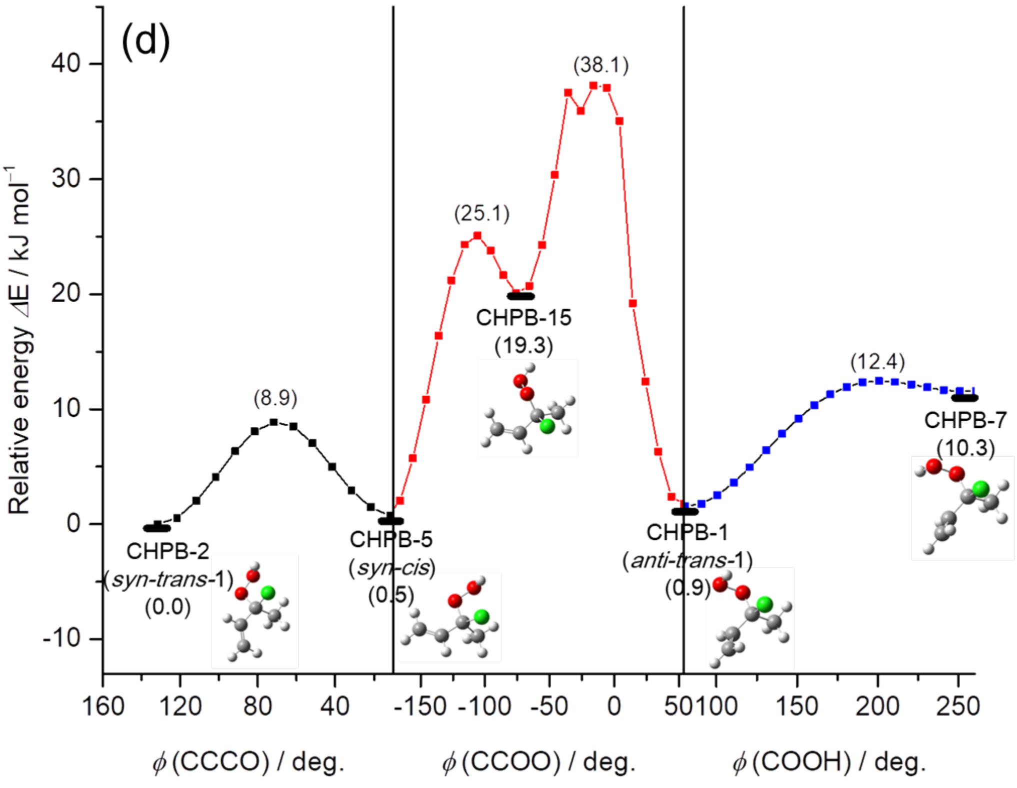
(d)
Relative energy ΔE / kJ mol⁻¹
40
30
20
10
0
-10
(8.9)
(25.1)
(38.1)
(12.4)
CHPB-2
(syn-trans-1)
(0.0)
CHPB-5
(syn-cis)
(0.5)
CHPB-15
(19.3)
CHPB-1
(anti-trans-1)
(0.9)
CHPB-7
(10.3)
160
120
80
40
-150
-100
-50
0
50
100
150
200
250
ϕ (CCCO) / deg.
ϕ (CCOO) / deg.
ϕ (COOH) / deg.

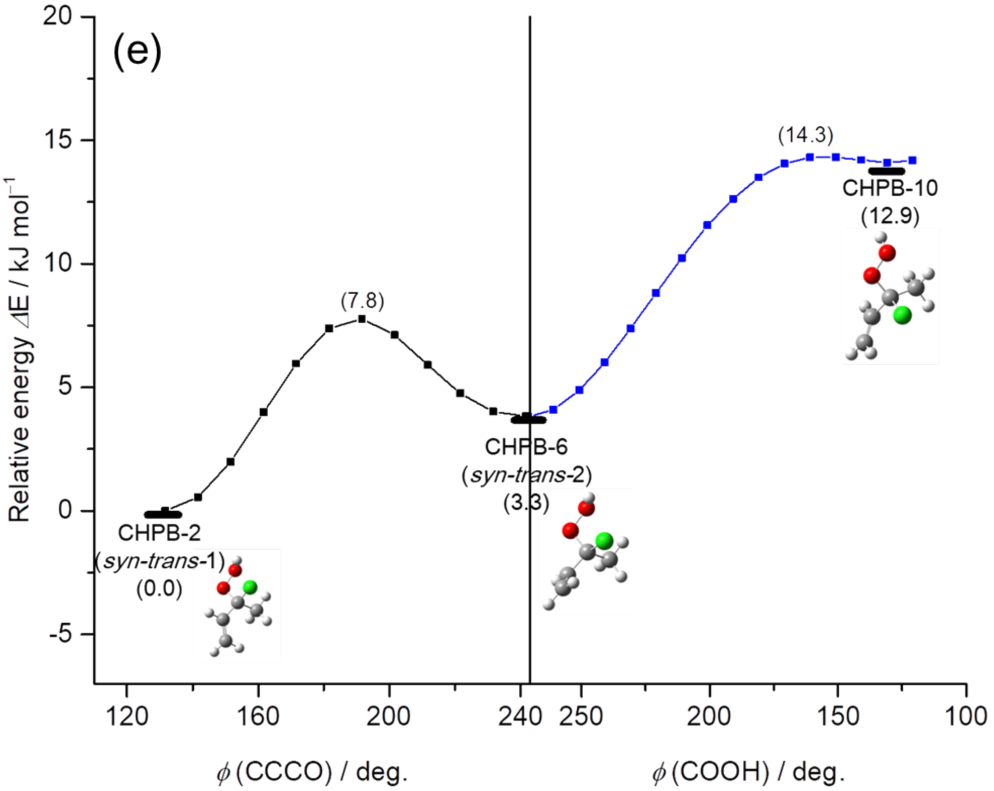
(e)
Relative energy ΔE / kJ mol⁻¹
(7.8)
(14.3)
CHPB-10
(12.9)
CHPB-6
(syn-trans-2)
(3.3)
CHPB-2
(syn-trans-1)
(0.0)
ϕ (CCCO) / deg.
ϕ (COOH) / deg.

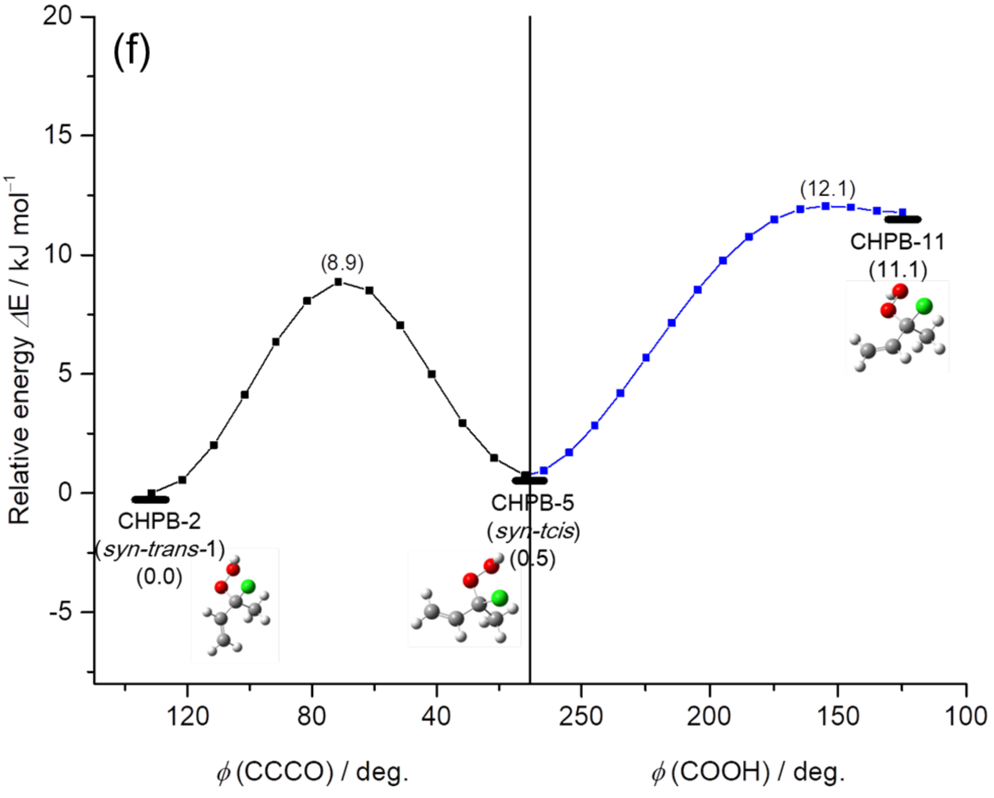
(f)
Relative energy ΔE / kJ mol⁻¹
(8.9)
(12.1)
CHPB-11
(11.1)
CHPB-5
(syn-tcis)
(0.5)
CHPB-2
(syn-trans-1)
(0.0)
ϕ (CCCO) / deg.
ϕ (COOH) / deg.

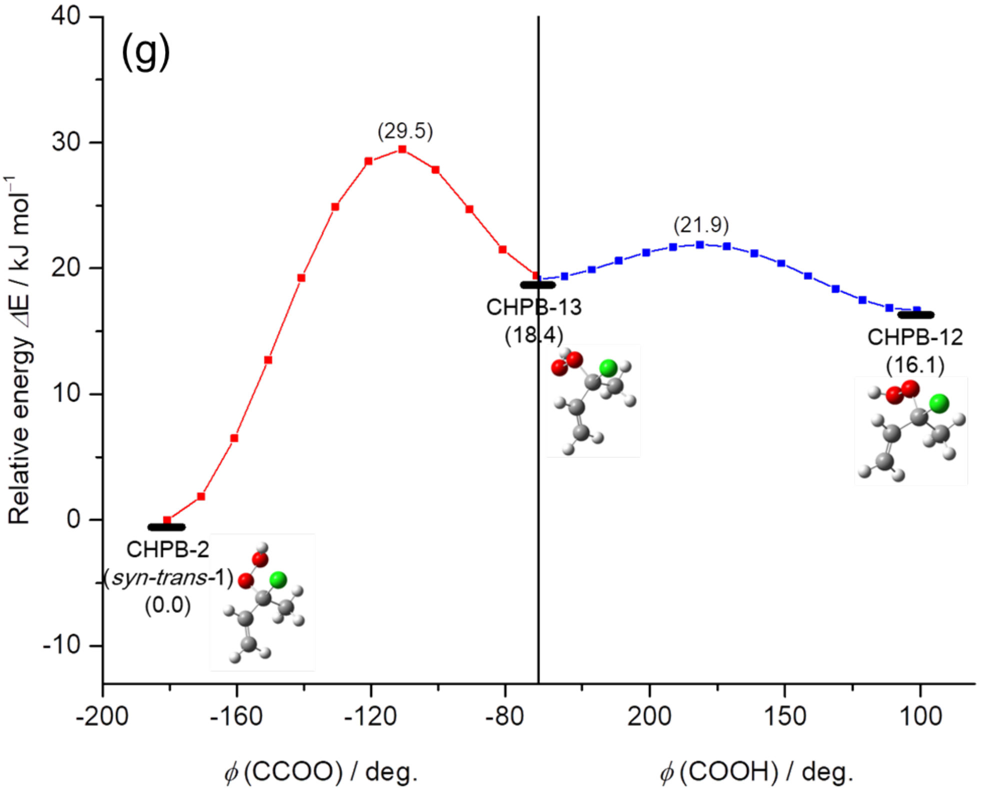

(g)
(29.5)
(21.9)
CHPB-13
(18.4)
CHPB-12
(16.1)
CHPB-2
(syn-trans-1)
(0.0)
Relative energy ΔE / kJ mol⁻¹
ϕ (CCOO) / deg.
ϕ (COOH) / deg.

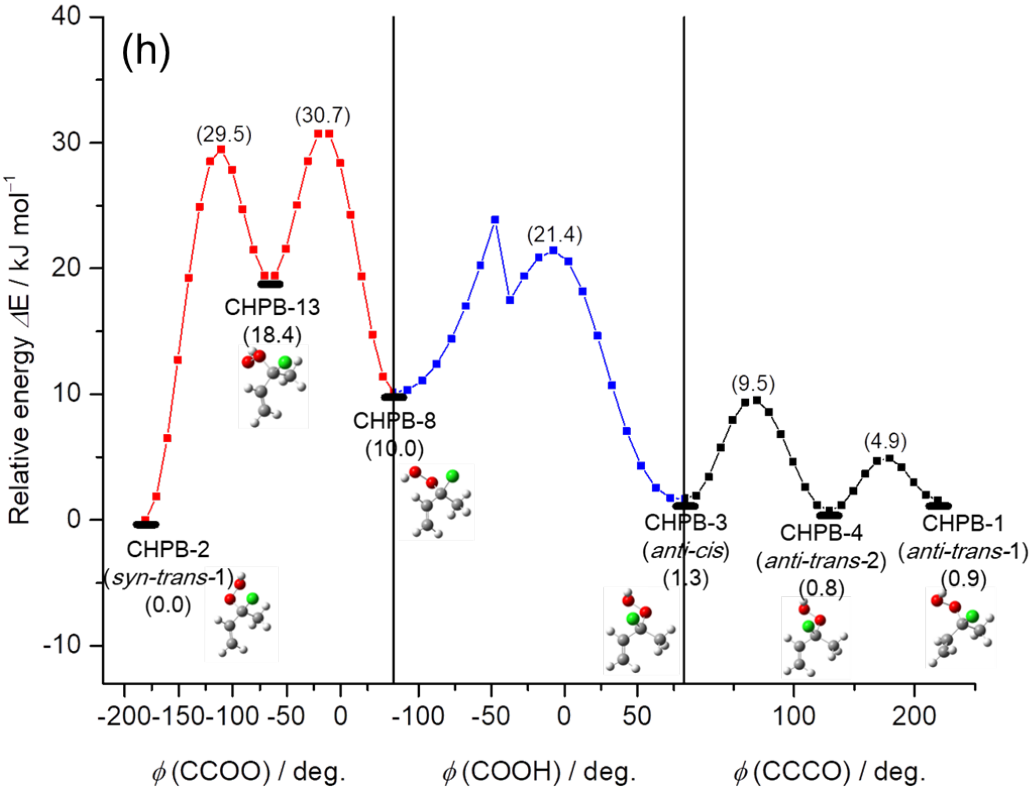

(h)
(29.5)
(30.7)
(21.4)
(9.5)
(4.9)
CHPB-13
(18.4)
CHPB-8
(10.0)
CHPB-2
(syn-trans-1)
(0.0)
CHPB-3
(anti-cis)
(1.3)
CHPB-4
(anti-trans-2)
(0.8)
CHPB-1
(anti-trans-1)
(0.9)
Relative energy ΔE / kJ mol⁻¹
ϕ (CCOO) / deg.
ϕ (COOH) / deg.
ϕ (CCCO) / deg.

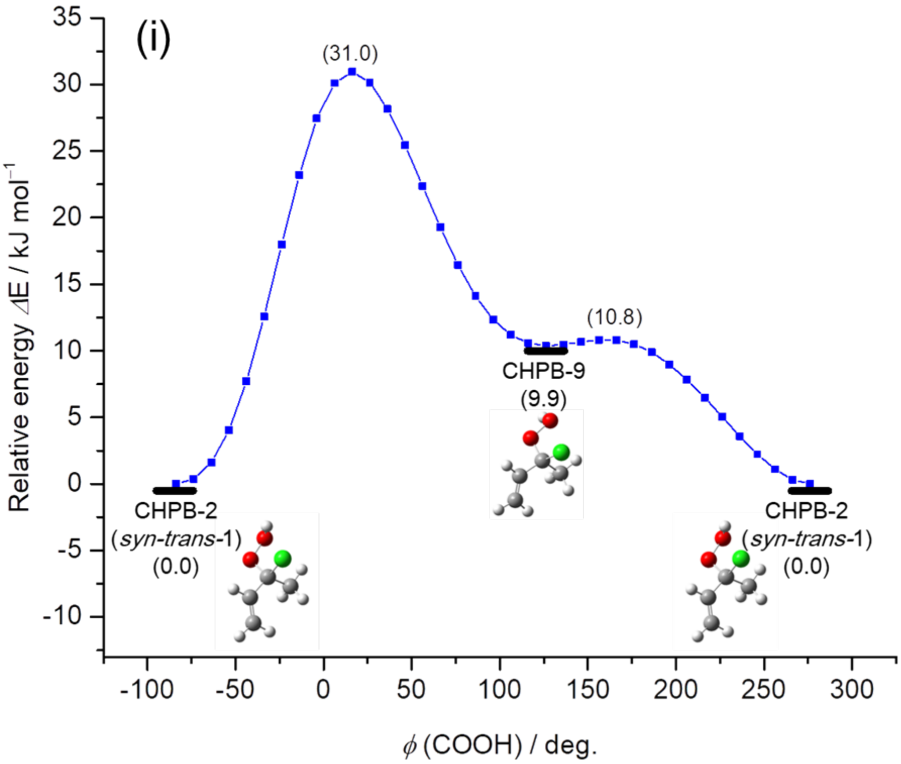

Figure S4. Potential energies for the conformation interconversion started from *syn-trans*1-CHPB (CHPB-2) calculated with the B3LYP/aug-cc-pVTZ method. (a) to CHPB-5 and CHPB-6. (b) to CHPB-1, CHPB-6, CHPB-7, and CHPB-16. (c) to CHPB-5, CHPB-14, and CHPB-15. (d) to CHPB-1, CHPB-5, CHPB-7, and CHPB-15. (e) to CHPB-6 and CHPB-10. (f) to CHPB-5 and CHPB-11. (g) to CHPB-12 and CHPB-13. (h) to CHPB-1, CHPB-3, CHPB-4, CHPB-8, and CHPB-13. (i) to CHPB-9. The scans for dihedral angles Φ(CCCO), Φ(CCOO), and Φ(COOH) are presented in black, red, and blue, respectively. Energies were calculated with the B3LYP/aug-cc-pVTZ method; the energy of CHPB-2 is set to zero.

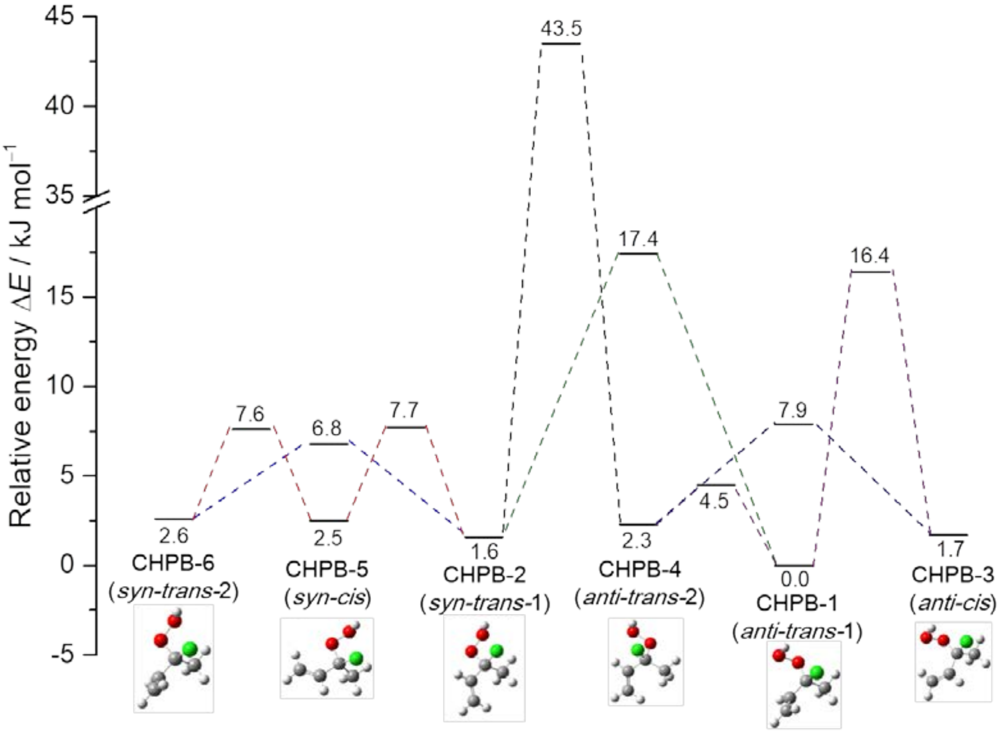

Figure S5. Potential energies for the interconversion among six lowest-energy conformers of CHPB calculated with the CCSD(T)/aug-cc-pVTZ//B3LYP/aug-cc-pVTZ method. Energies relative to CHPB-1 are in kJ mol^{-1}; zero-point energies are corrected with the B3LYP/aug-cc-pVTZ method.

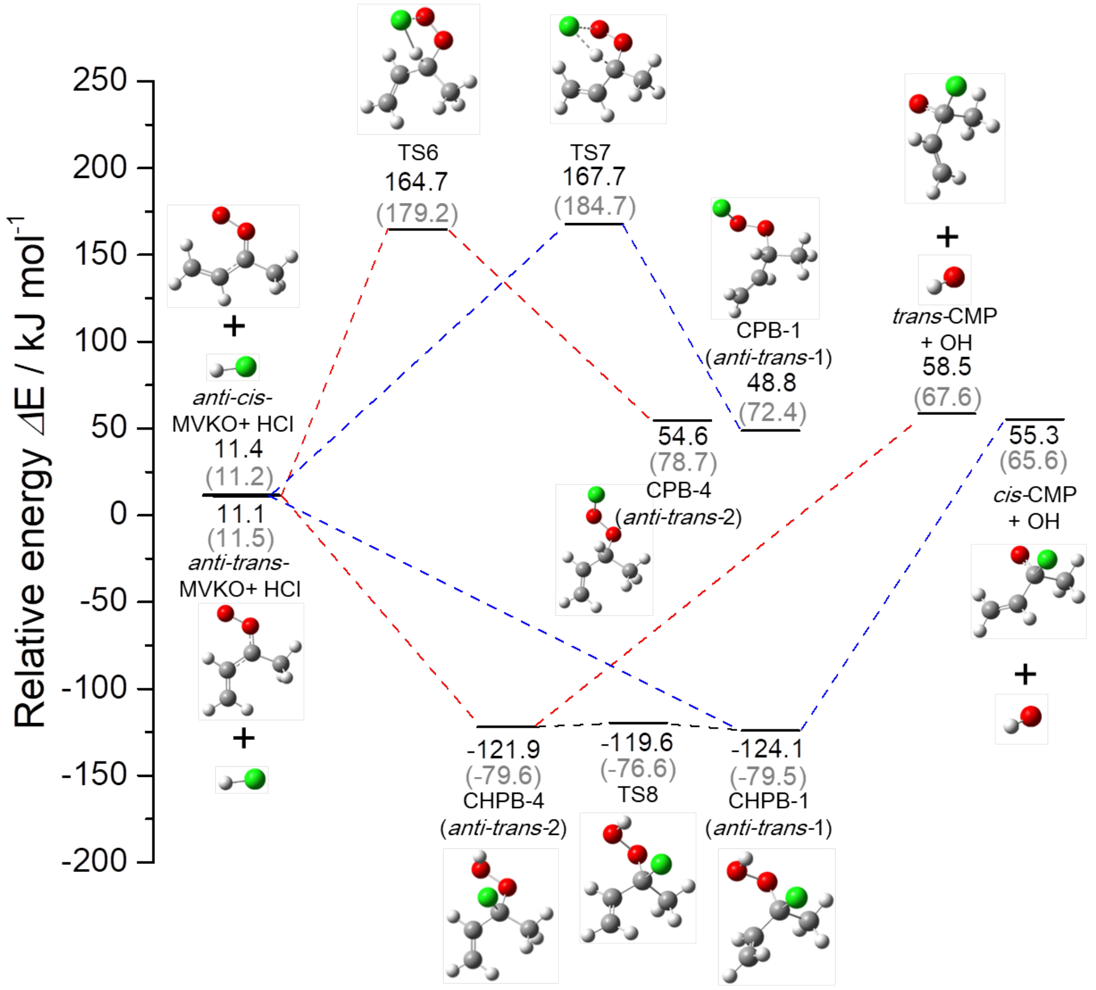

Figure S6. Reaction pathway scheme (RPS) of *anti*-MVKO + HCl predicted with the CCSD(T)/aug-cc-pVTZ//B3LYP/aug-cc-pVTZ method. The dashed red and dashed blue lines indicate the reactions of HCl with *anti-trans*-MVKO and *anti-cis*-MVKO, respectively. Energies calculated with the B3LYP/aug-cc-pVTZ method are listed in parentheses for comparison. Energies in kJ mol^{-1} were corrected for zero-point vibrational energies calculated with the B3LYP/aug-cc-pVTZ method. CHPB indicates $(C_2H_3)CCl(CH_3)OOH$, CPB indicates $(C_2H_3)CH(CH_3)OOCl$, and CMP indicates the $(C_2H_3)CCl(CH_3)O$ radical.

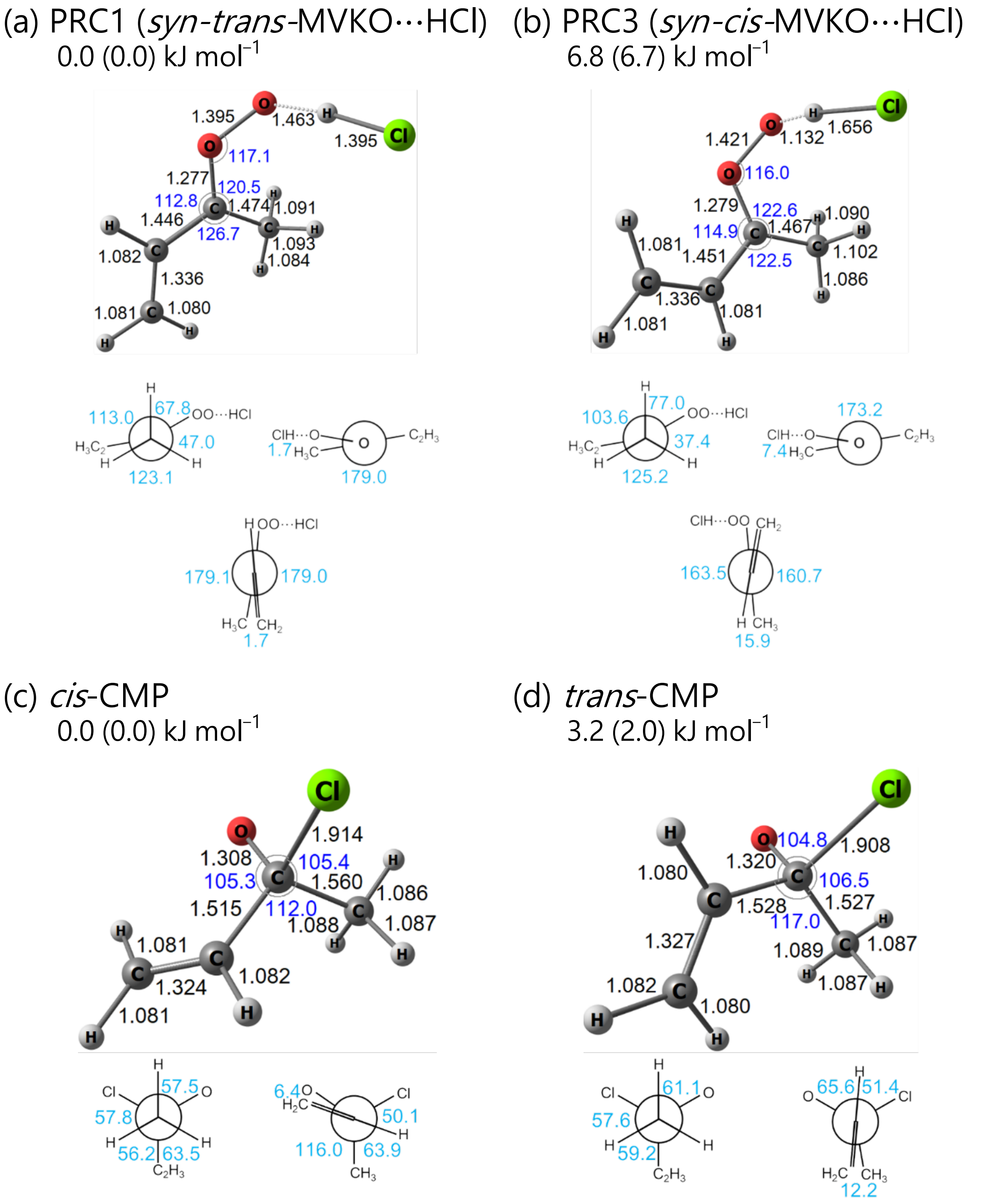

Figure S7. Geometries of two pre-reactive complexes and two conformers of $(C_2H_3)C(CH_3)(Cl)O$ (CMP) calculated with the B3LYP/aug-cc-pVTZ method. (a) PRC1, (b) PRC3, (c) *cis*-CMP, (d) *trans*-CMP. Bond lengths are in Å and bond angles are in degrees. Energies were calculated with the CCSD(T)/aug-cc-pVTZ//B3LYP/aug-cc-pVTZ method; those in parentheses were calculated with the B3LYP/aug-cc-pVTZ method.

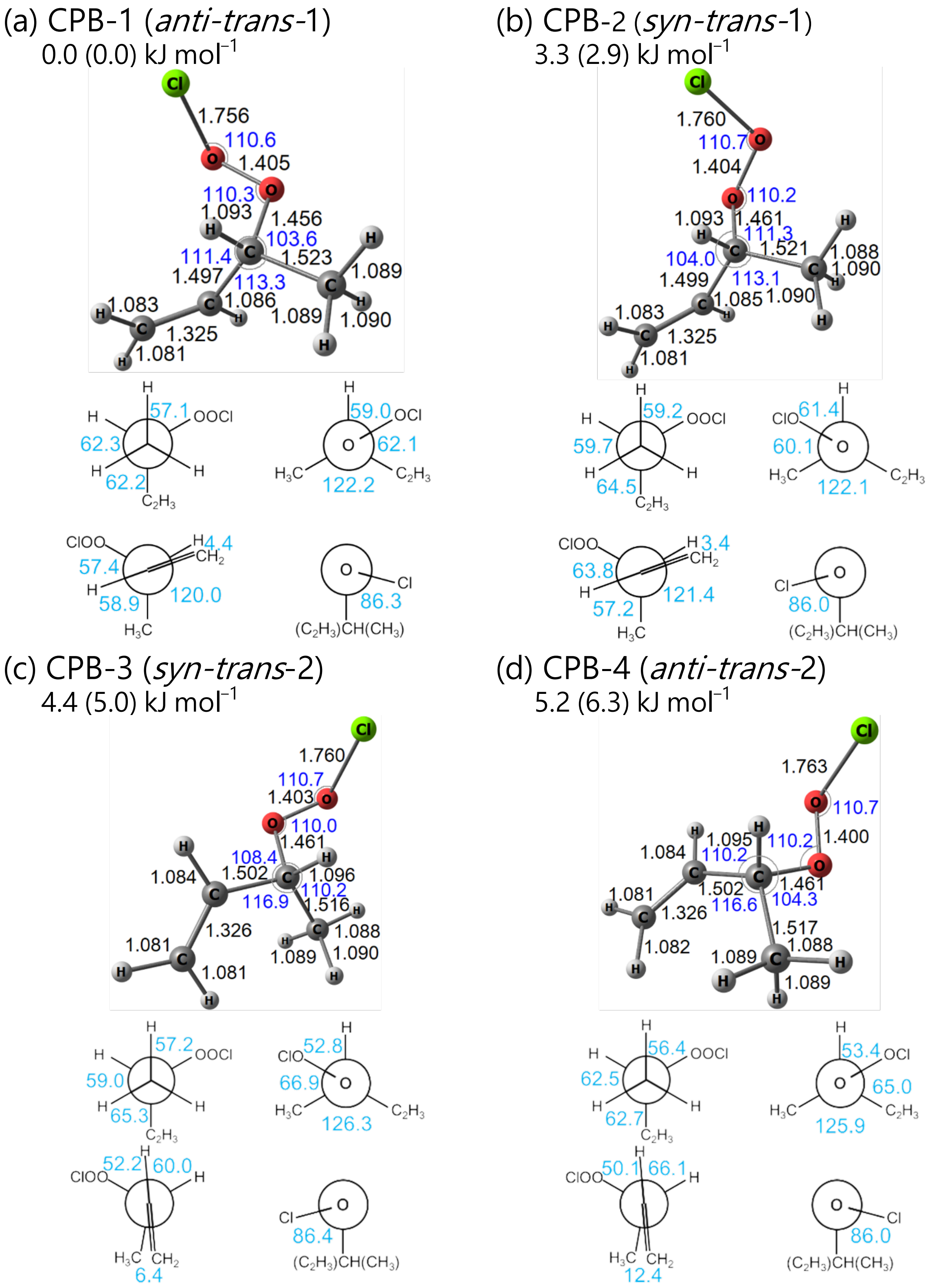

Figure S8. Geometries of four conformers of $(C_2H_3)CH(CH_3)OOCl$ (CPB) calculated with the B3LYP/aug-cc-pVTZ method. (a) CPB-1, (b) CPB-2, (c) CPB-3, and (d) CPB-4. Bond lengths are in Å and bond angles are in degrees. Energies were calculated with the CCSD(T)/aug-cc-pVTZ//B3LYP/aug-cc-pVTZ method; those in parentheses were calculated with the B3LYP/aug-cc-pVTZ method.

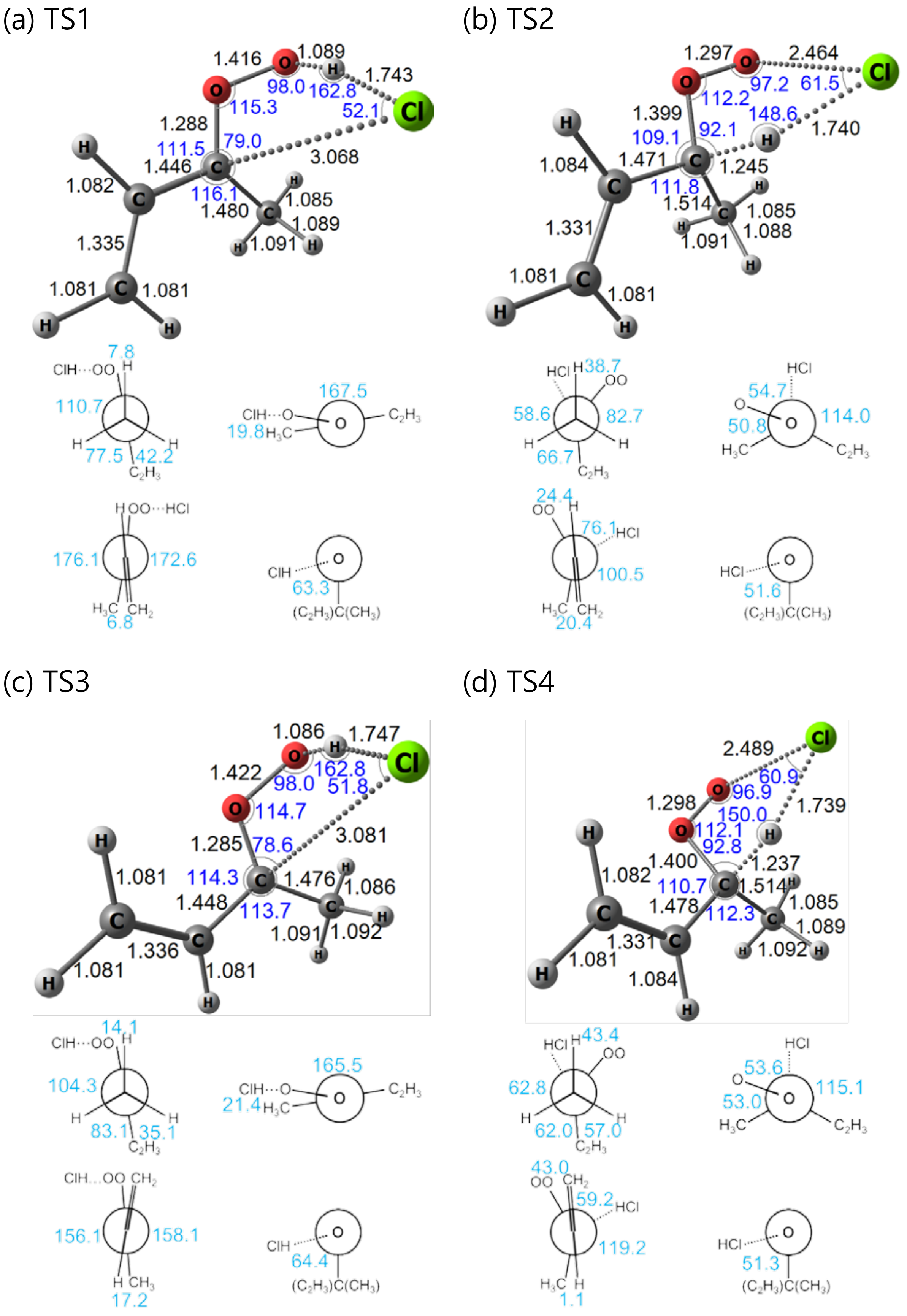
(a) TS1
(b) TS2
(c) TS3
(d) TS4

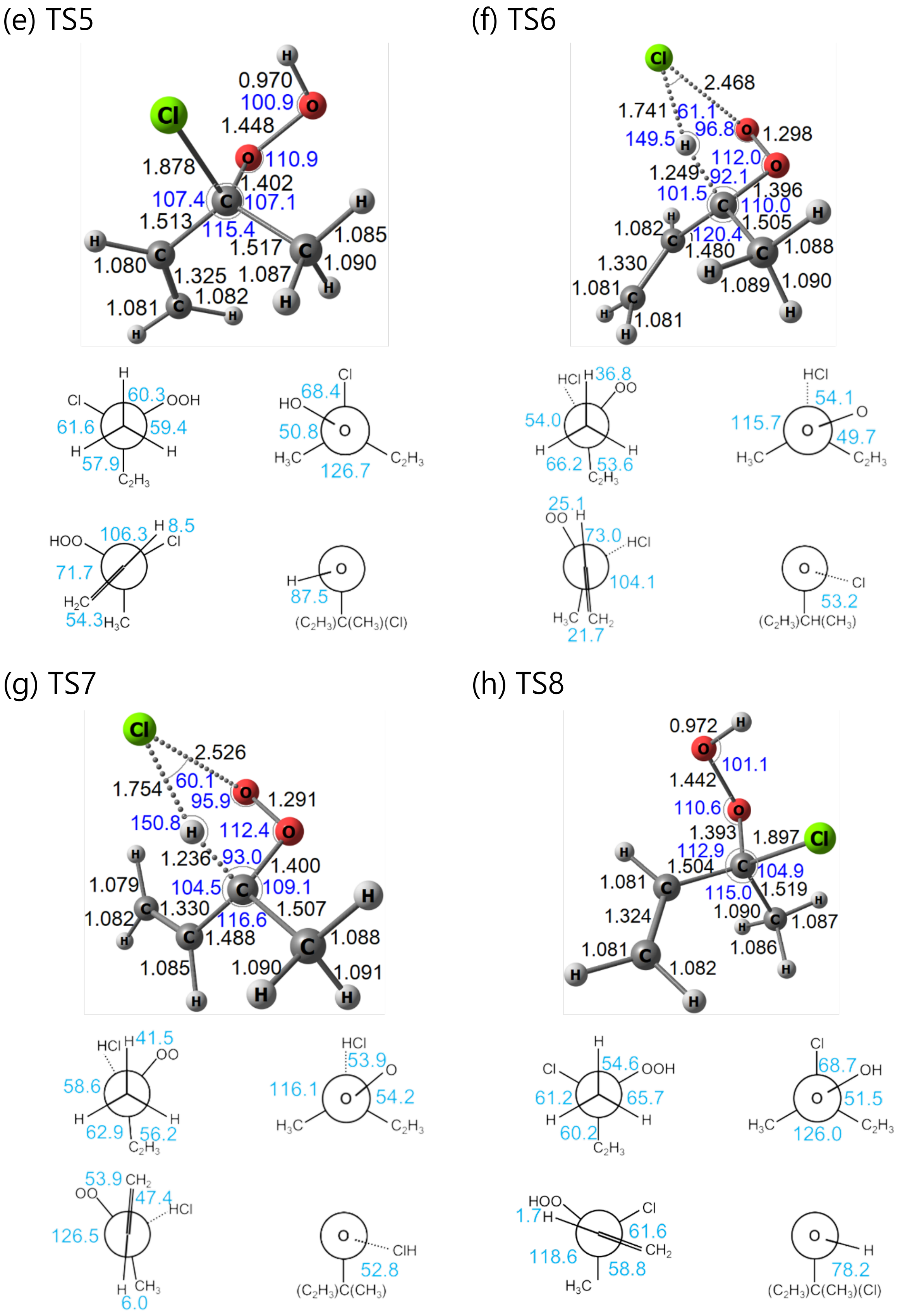

Figure S9. Geometries of TS1−TS8 calculated with the B3LYP/aug-cc-pVTZ method. (a) TS1, (b) TS2, (c) TS3, (d) TS4, (e) TS5, (f) TS6, (g) TS7, and (h) TS8. Bond lengths are in Å and bond angles are in degrees.

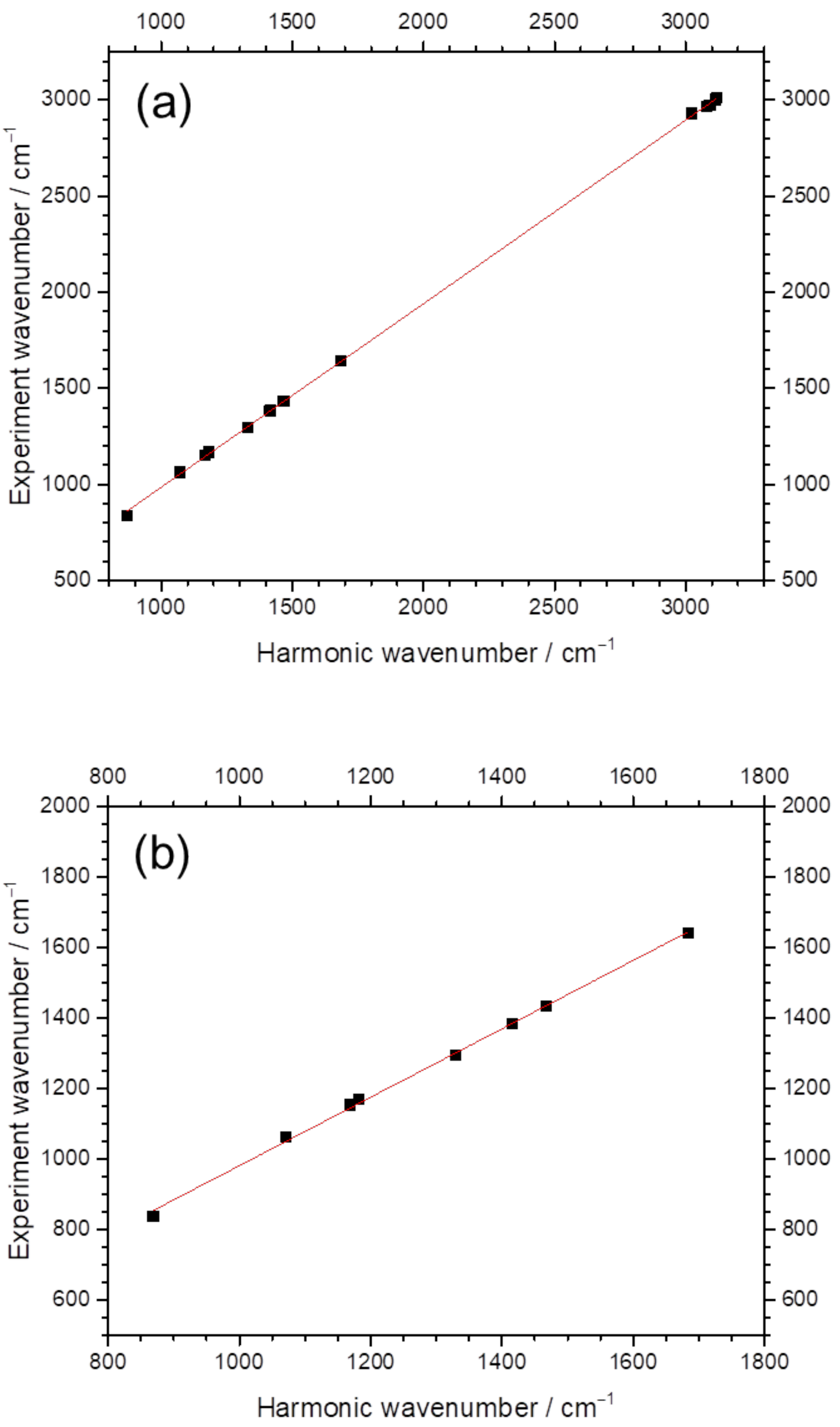

Figure S10. Comparison of experimentally observed wavenumbers (cm^{-1}) with the harmonic vibrational wavenumbers of (*Z*)-(CH_2I)HCC(CH_3)I predicted with the B3LYP/aug-cc-pVTZ-pp method. (a) Region 800−3300 cm^{-1}. Fitted equation is y = (0.955 ±0.003) x + (30.7±6.7). (b) Region 800−2000 cm^{-1}. Fitted equation is y = (0.971±0.016) x + (10.4±20.7).

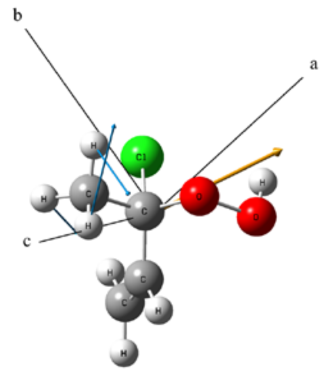

v_9, $a : b : c$

= 0.85 : 0.12 : 0.03

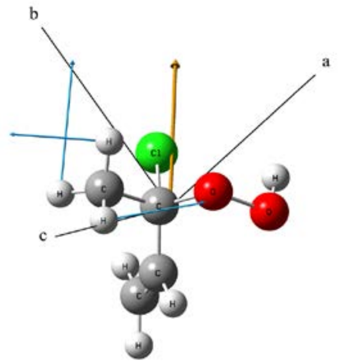

v_{10}, $a : b : c$

= 0.02 : 055 : 0.43

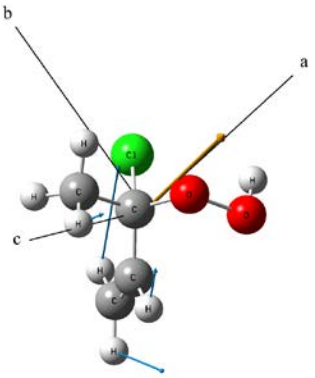

v_{11}, $a : b : c$

= 0.81 : 0.01 : 0.18

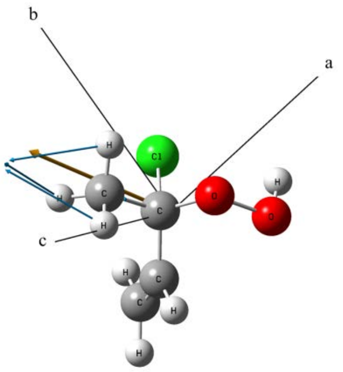

v_{12}, $a : b : c$

= 0.00 : 0.28 : 0.72

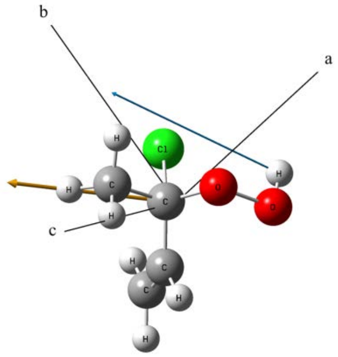

v_{13}, $a : b : c$

= 0.14 : 0.32 : 0.54

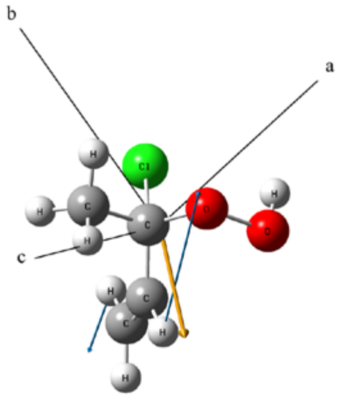

v_{14}, $a : b : c$

= 0.02 : 0.55 : 0.43

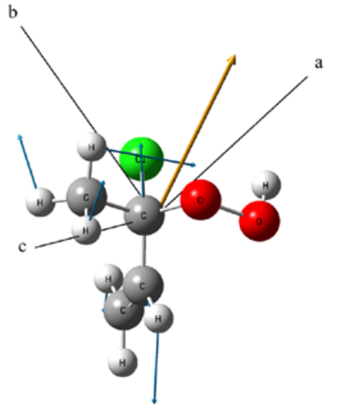

v_{15}, $a : b : c$

= 0.87 : 0.13 : 0.00

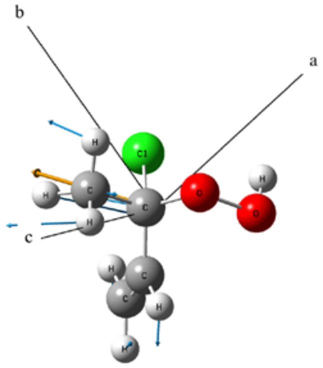

v_{16}, $a : b : c$

= 0.46 : 0.48 : 0.06

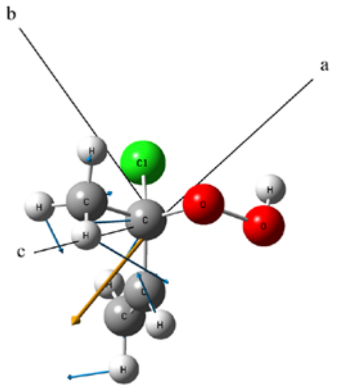

v_{17}, $a : b : c$

= 0.05 : 0.12 : 0.83

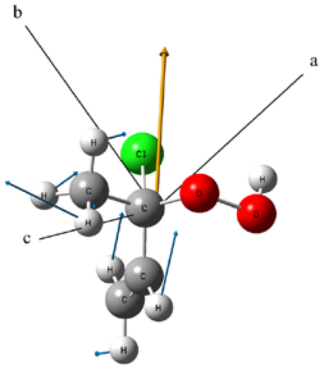

ν_{18}, $a : b : c$

= 0.09 : 0.64 : 0.27

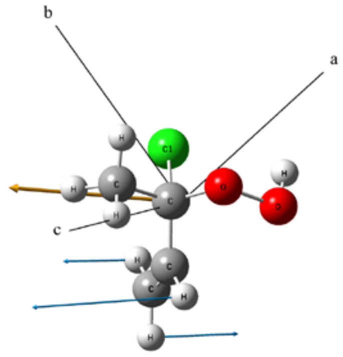

ν_{19}, $a : b : c$

= 0.16 : 0.33 : 0.51

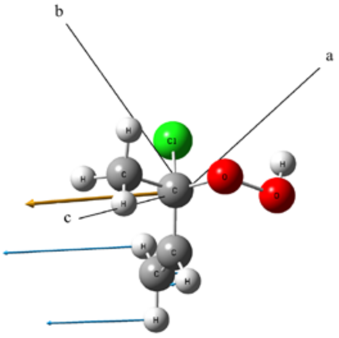

ν_{20}, $a : b : c$

= 0.23 : 0.17 : 0.60

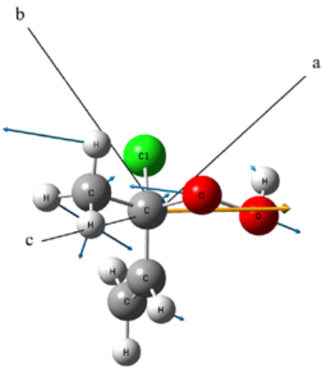

ν_{21}, $a : b : c$

= 0.64 : 0.41 : 0.05

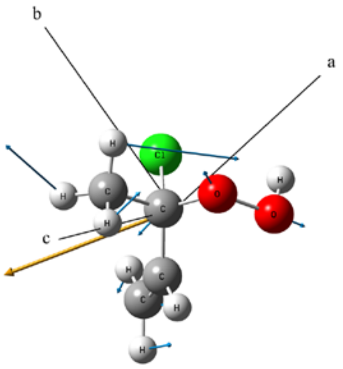

ν_{22}, $a : b : c$

= 0.77 : 0.08 : 0.15

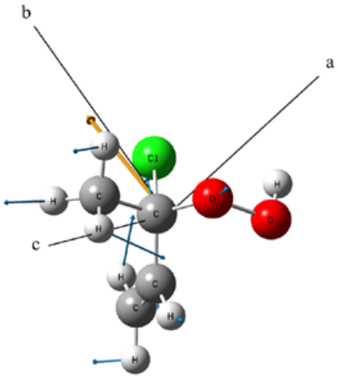

ν_{23}, $a : b : c$

= 0.11 : 0.67 : 0.22

Figure S11. Displacement vectors (blue arrows) and directions of dipole derivatives (brown arrows) for modes ν_9–ν_{23} of CHPB-1 predicted with the B3LYP/aug-cc-pVTZ method. Molecular rotational axes are represented with labels *a*, *b*, and *c*; *a* : *b* : *c* represents the mixing ratio of bands of types *a*, *b*, and *c* in each transition.

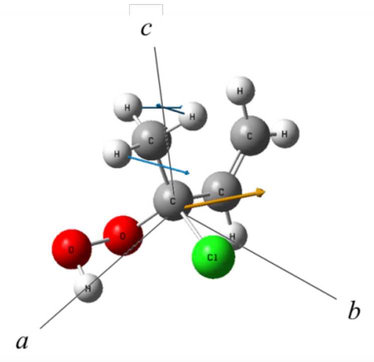

ν_{9}, $a : b : c$

= 0.04 : 0.69 : 0.26

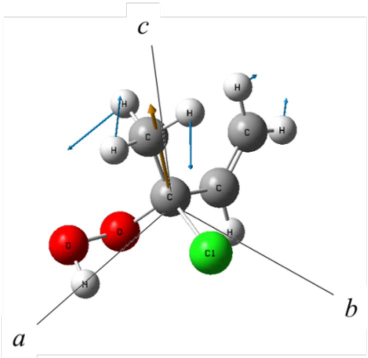

ν_{10}, $a : b : c$

= 0.02 : 0.07 : 0.91

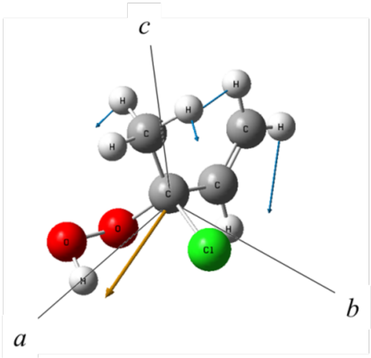

ν_{11}, $a : b : c$

= 0.90 : 0.01 : 0.09

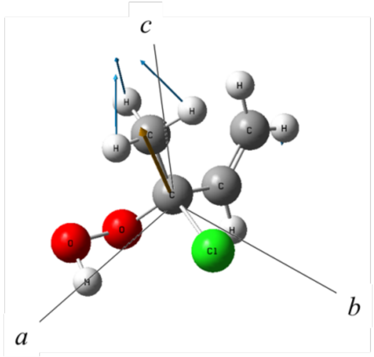

ν_{12}, $a : b : c$

= 0.03 : 0.01 : 0.96

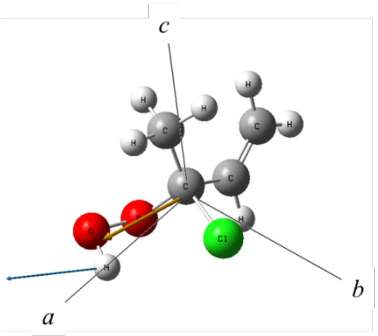

ν_{13}, $a : b : c$

= 0.95 : 0.03 : 0.02

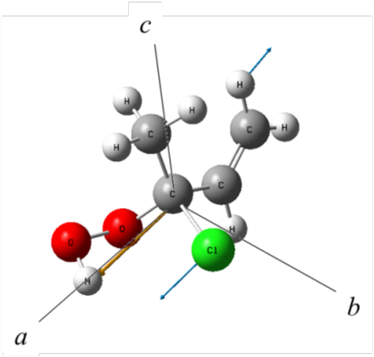

ν_{14}, $a : b : c$

= 0.36 : 0.09 : 0.55

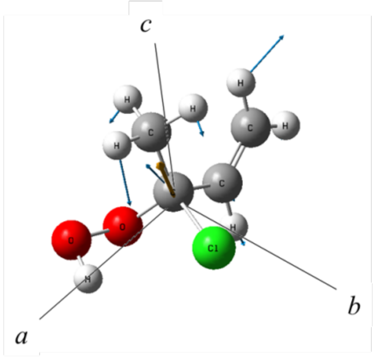

ν_{15}, $a : b : c$

= 0.15 : 0.05 : 0.80

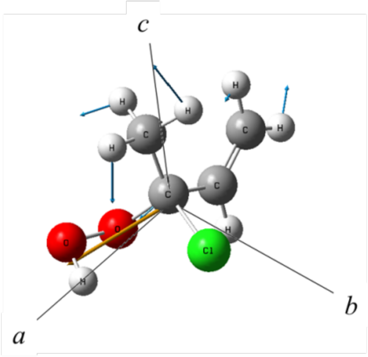

ν_{16}, $a : b : c$

= 0.74 : 0.18 : 0.08

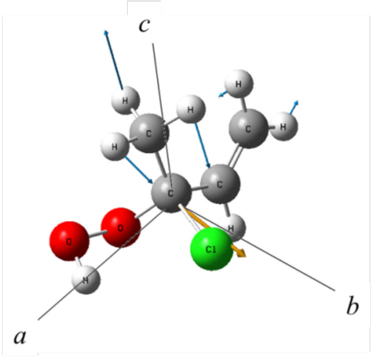

ν_{17}, $a : b : c$

= 0.04 : 0.95 : 0.01

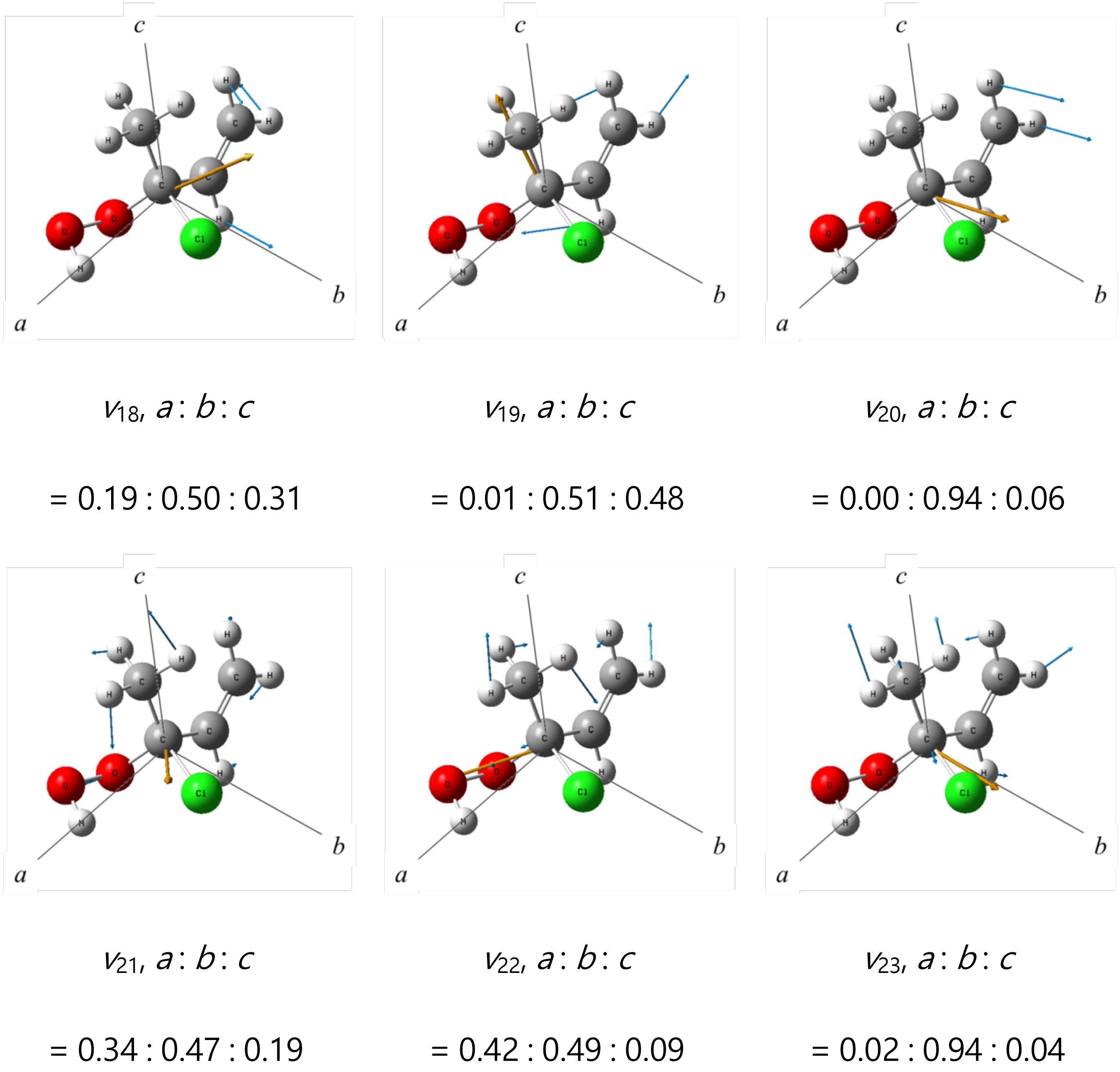

Figure S12. Displacement vectors (blue arrows) and directions of dipole derivatives (brown arrows) for modes ν_9–ν_{23} of CHPB-2 predicted with the B3LYP/aug-cc-pVTZ method. Molecular rotational axes are represented with labels *a*, *b*, and *c*; *a* : *b* : *c* represents the mixing ratio of bands of types *a*, *b*, and *c* in each transition.

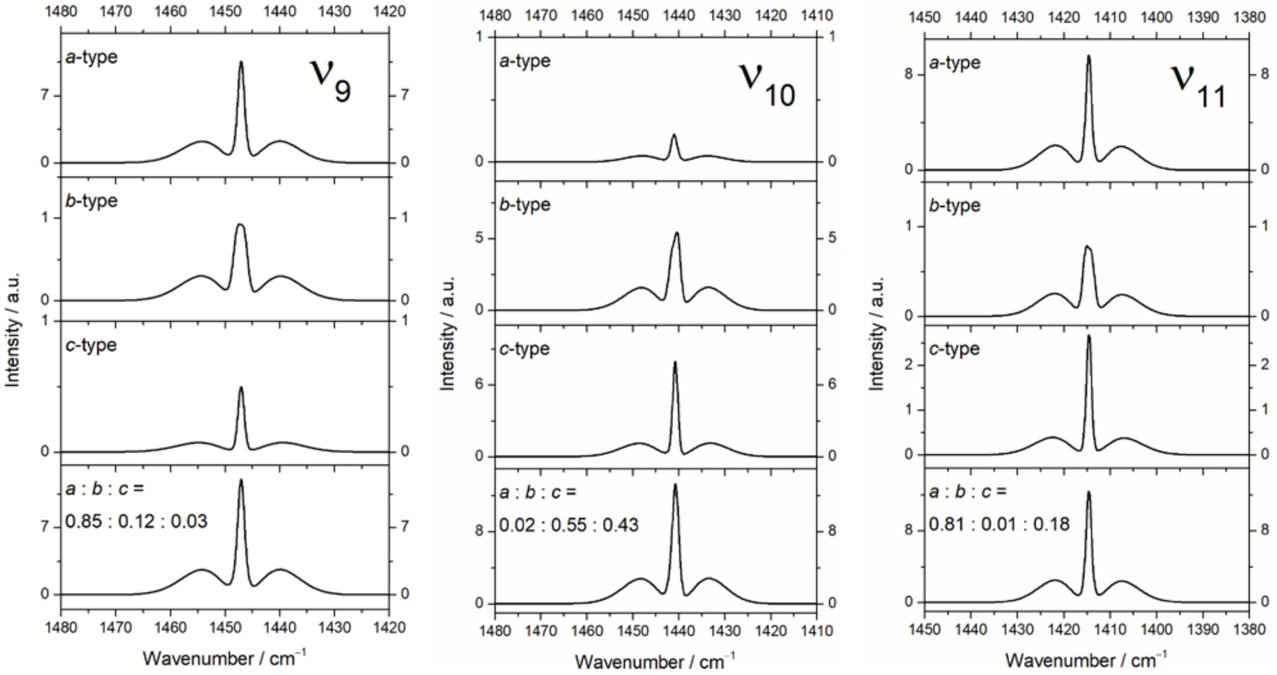
ν9
a-type
b-type
c-type
a : b : c =
0.85 : 0.12 : 0.03
ν10
a-type
b-type
c-type
a : b : c =
0.02 : 0.55 : 0.43
ν11
a-type
b-type
c-type
a : b : c =
0.81 : 0.01 : 0.18
Intensity / a.u.
Wavenumber / cm-1

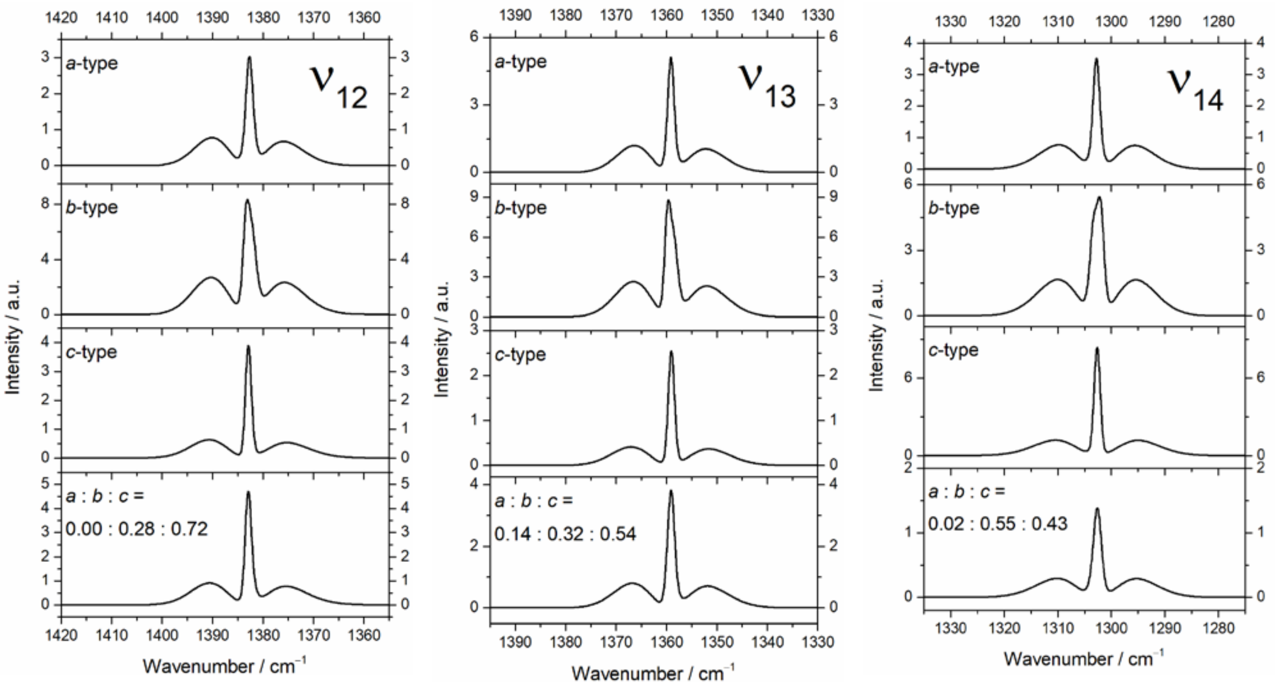
ν12
a-type
b-type
c-type
a : b : c =
0.00 : 0.28 : 0.72
ν13
a-type
b-type
c-type
a : b : c =
0.14 : 0.32 : 0.54
ν14
a-type
b-type
c-type
a : b : c =
0.02 : 0.55 : 0.43
Intensity / a.u.
Wavenumber / cm-1

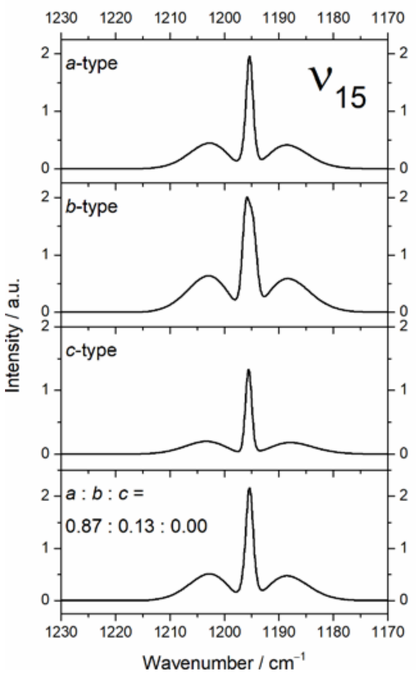
ν15
a-type
b-type
c-type
a : b : c =
0.87 : 0.13 : 0.00
Intensity / a.u.
Wavenumber / cm-1

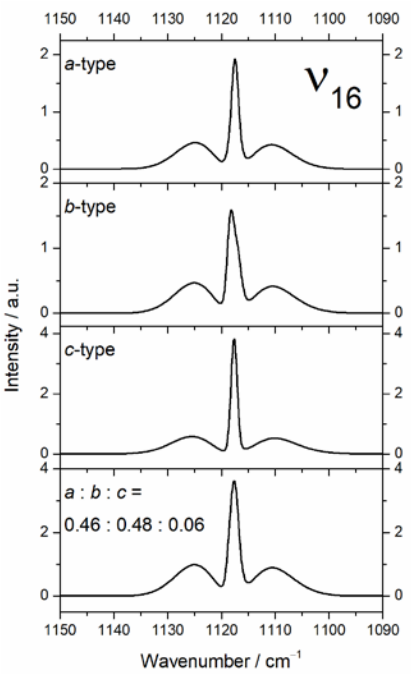
ν16
a-type
b-type
c-type
a : b : c =
0.46 : 0.48 : 0.06
Intensity / a.u.
Wavenumber / cm-1

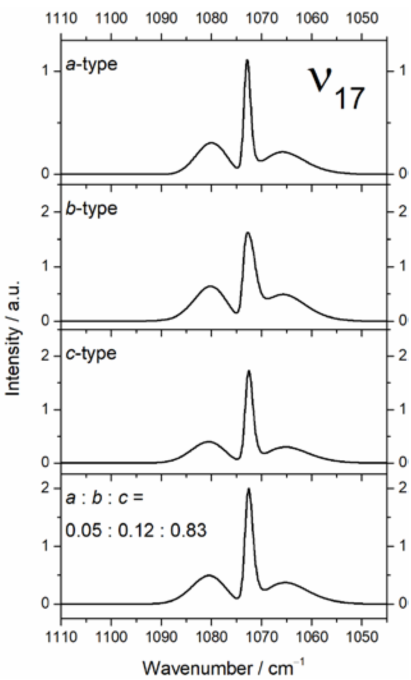
ν17
a-type
b-type
c-type
a : b : c =
0.05 : 0.12 : 0.83
Intensity / a.u.
Wavenumber / cm-1

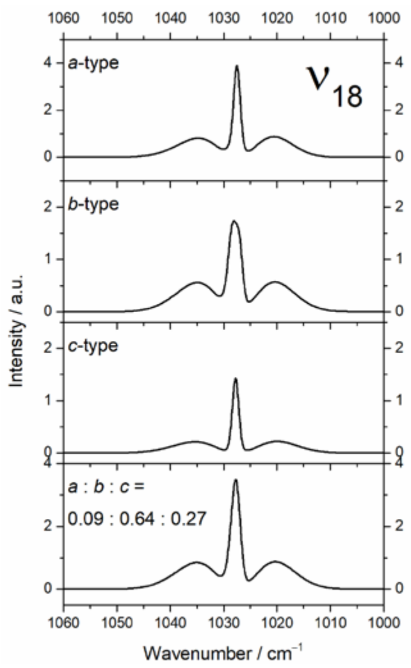
ν18
a-type
b-type
c-type
a : b : c =
0.09 : 0.64 : 0.27
Intensity / a.u.
Wavenumber / cm-1

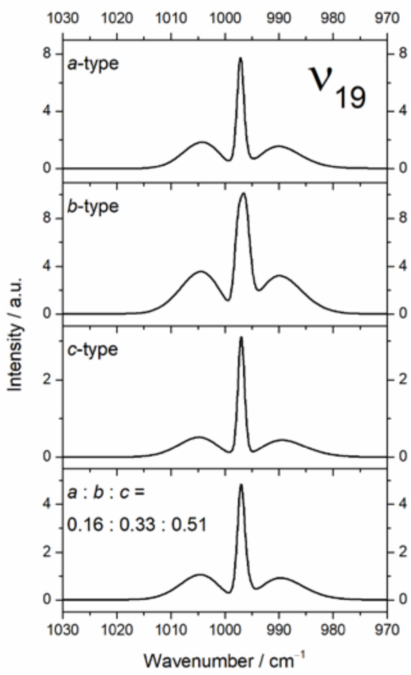
ν19
a-type
b-type
c-type
a : b : c =
0.16 : 0.33 : 0.51
Intensity / a.u.
Wavenumber / cm-1

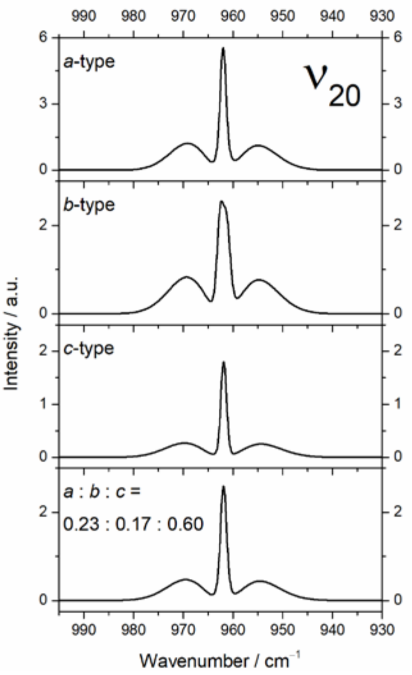
ν20
a-type
b-type
c-type
a : b : c =
0.23 : 0.17 : 0.60
Intensity / a.u.
Wavenumber / cm-1

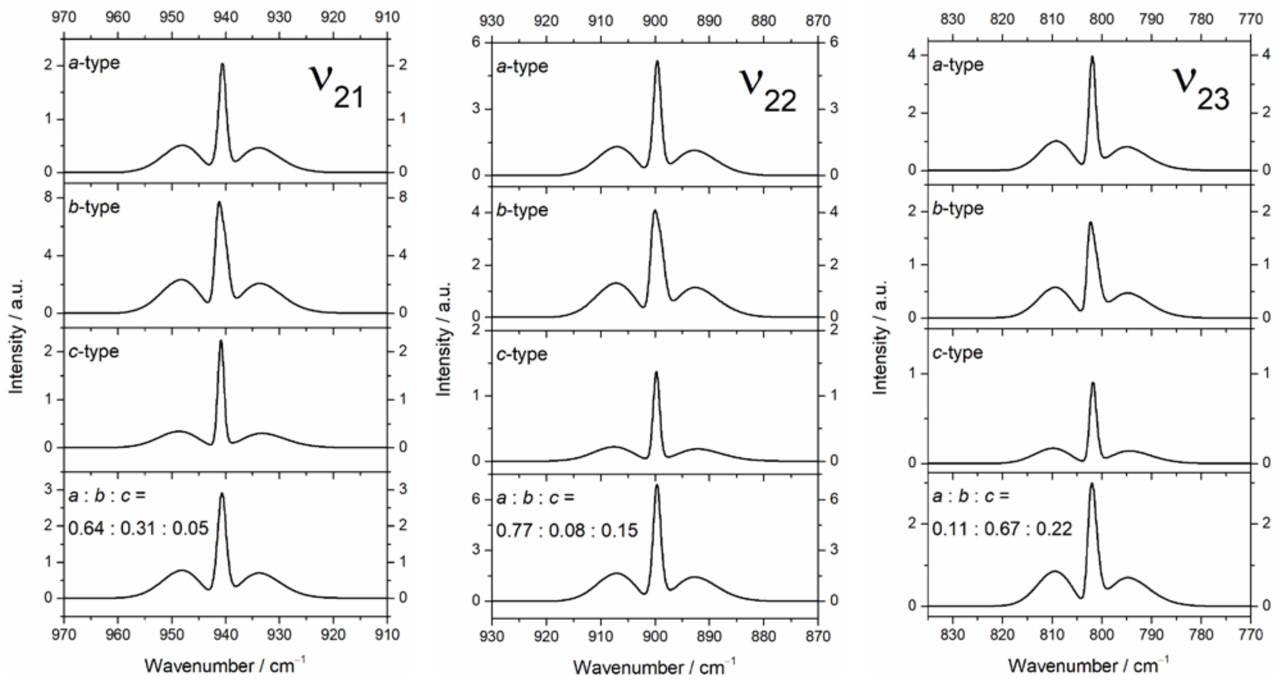

Figure S13. Rotational contours simulated for modes ν_9–ν_{23} of CHPB-1. Rotational parameters used in the PGOPHER simulations are listed in Table S9, J_{max} = 150, T = 298 K, and Gaussian width (FWHM) = 1.28 cm^{-1}; type ratios for the resultant spectra are listed in figures.

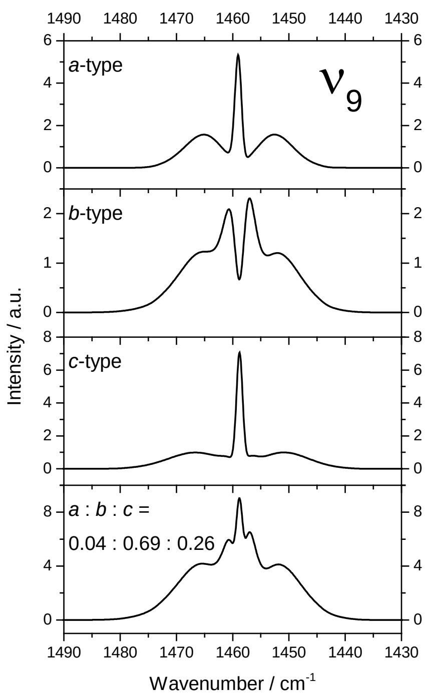
ν9
a-type
b-type
c-type
a : b : c =
0.04 : 0.69 : 0.26
Intensity / a.u.
Wavenumber / cm-1

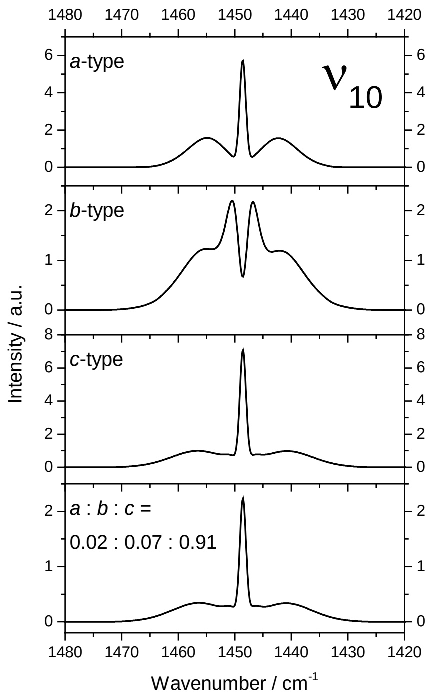
ν10
a-type
b-type
c-type
a : b : c =
0.02 : 0.07 : 0.91
Intensity / a.u.
Wavenumber / cm-1

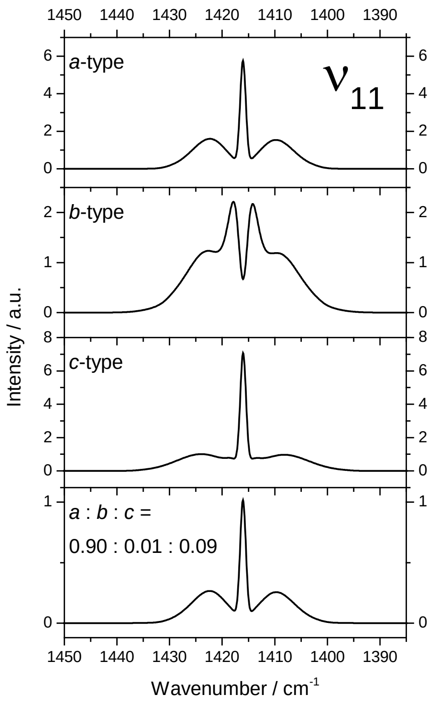
ν11
a-type
b-type
c-type
a : b : c =
0.90 : 0.01 : 0.09
Intensity / a.u.
Wavenumber / cm-1

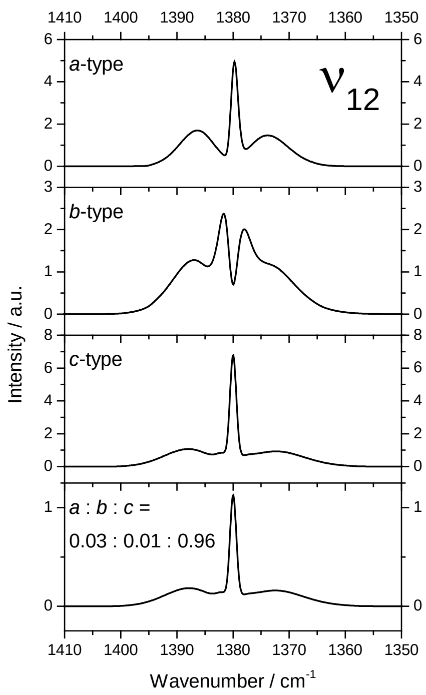
ν12
a-type
b-type
c-type
a : b : c =
0.03 : 0.01 : 0.96
Intensity / a.u.
Wavenumber / cm-1

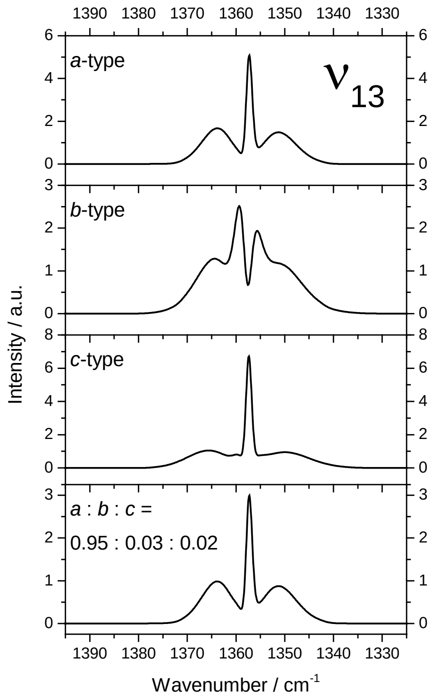
ν13
a-type
b-type
c-type
a : b : c =
0.95 : 0.03 : 0.02
Intensity / a.u.
Wavenumber / cm-1

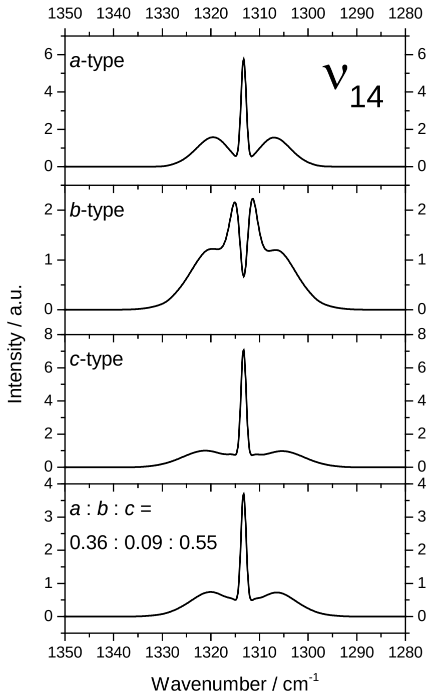
ν14
a-type
b-type
c-type
a : b : c =
0.36 : 0.09 : 0.55
Intensity / a.u.
Wavenumber / cm-1

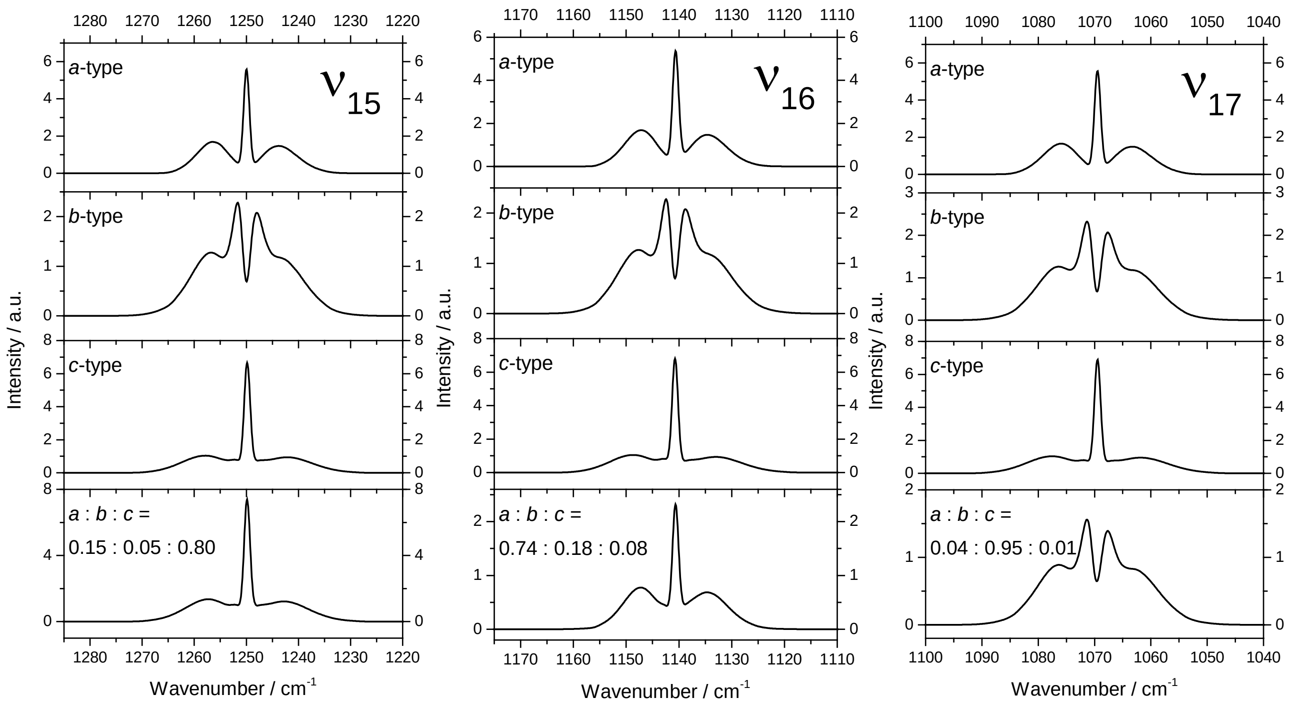
ν15
ν16
ν17
a-type
b-type
c-type
a : b : c =
0.15 : 0.05 : 0.80
0.74 : 0.18 : 0.08
0.04 : 0.95 : 0.01
Intensity / a.u.
Wavenumber / cm-1

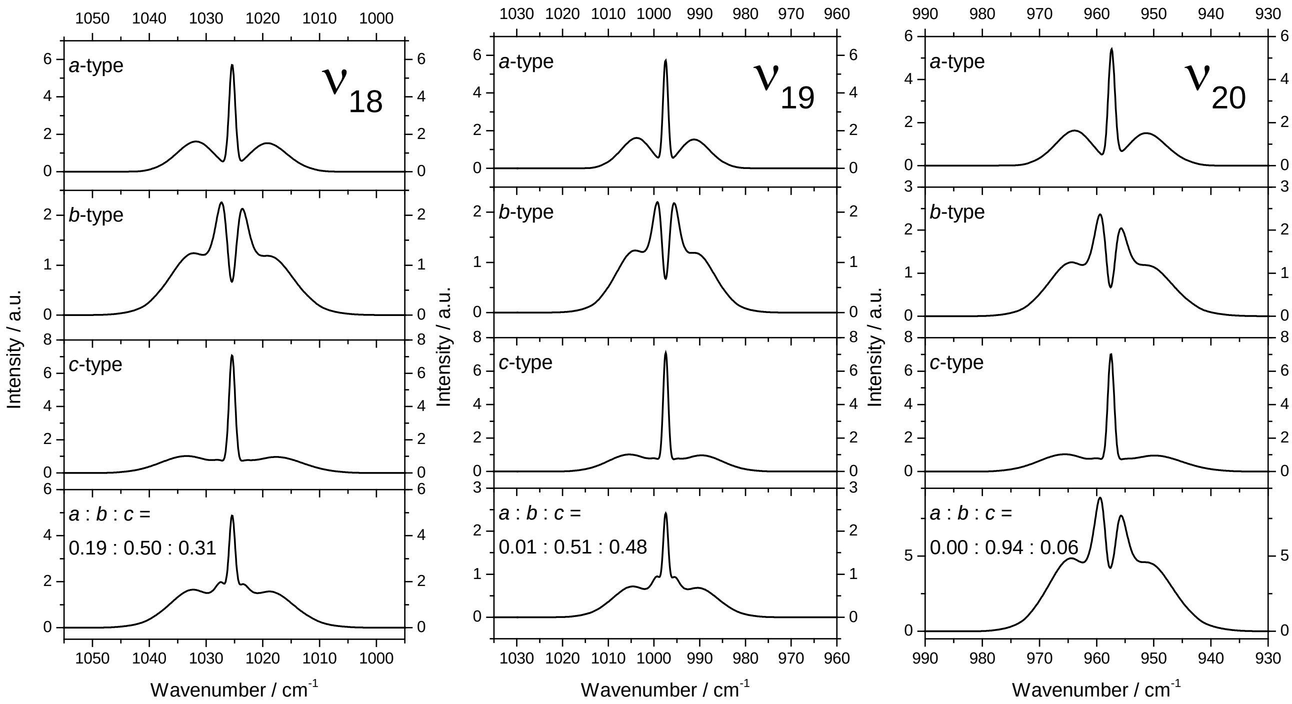
ν18
ν19
ν20
a-type
b-type
c-type
a : b : c =
0.19 : 0.50 : 0.31
0.01 : 0.51 : 0.48
0.00 : 0.94 : 0.06
Intensity / a.u.
Wavenumber / cm-1

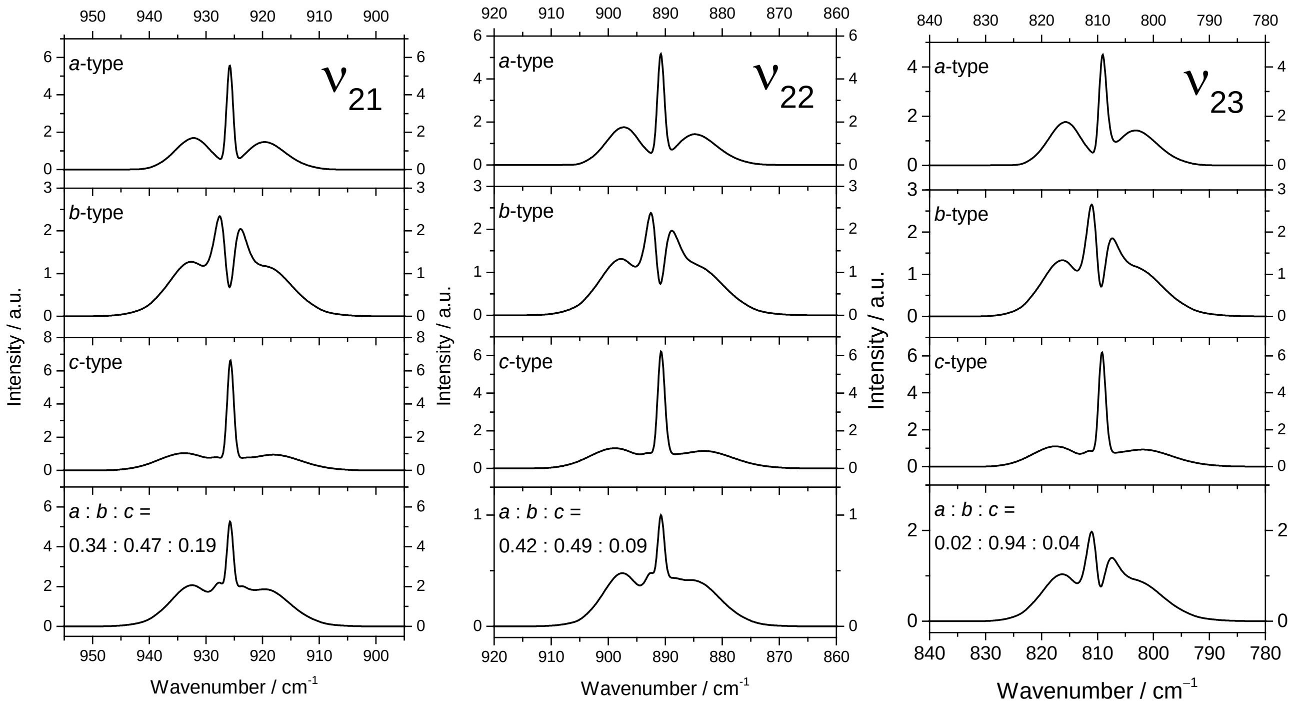

Figure S14. Rotational contours simulated for modes ν_9–ν_{23} of CHPB-2. Rotational parameters used in the PGOPHER simulations are listed in Table S9; J_{max} = 150, T = 298 K, and Gaussian width (FWHM) = 1.28 cm^{-1}; type ratios for the resultant spectra are listed in figures.

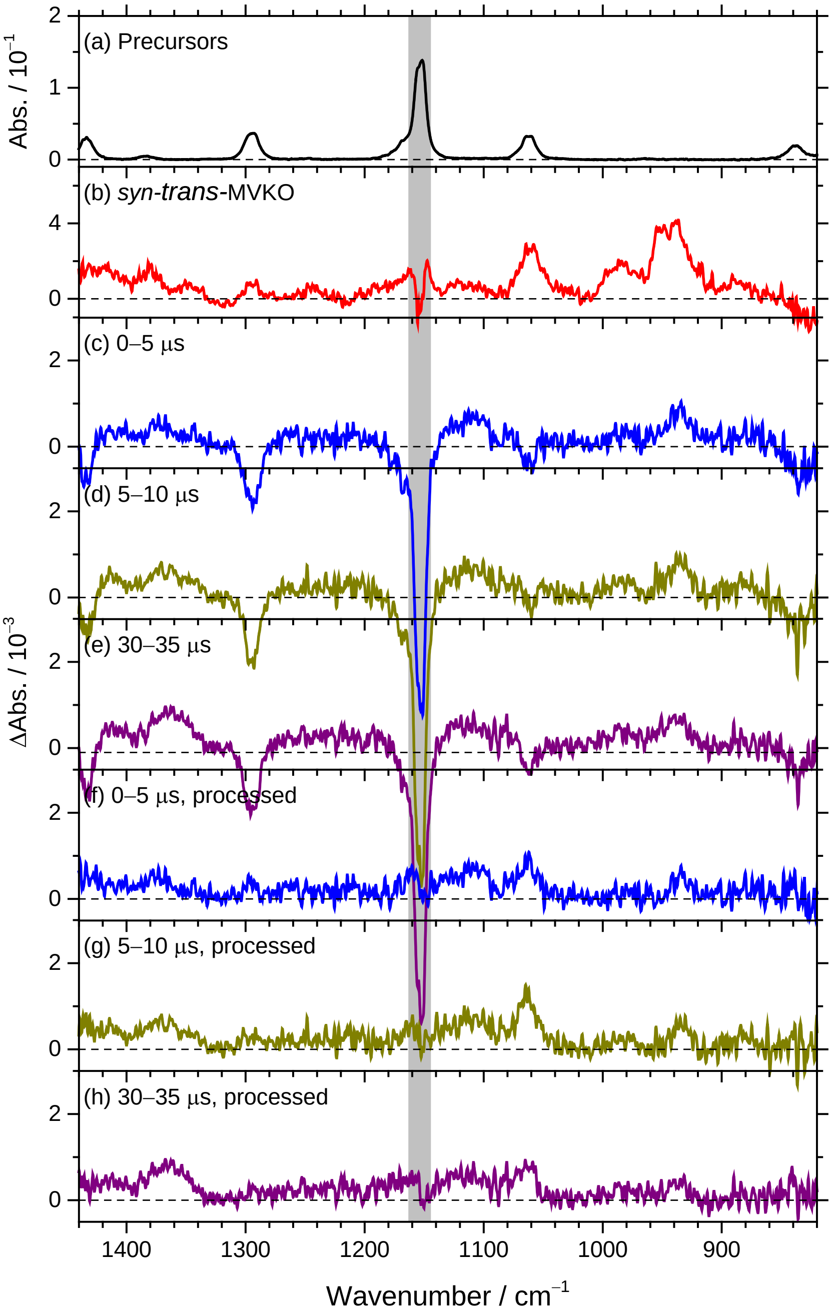

Figure S15. IR spectra recorded with an external ADC upon photolysis at 248 nm of a flowing mixture of (*Z*)-$(CH_2I)HC{=}C(CH_3)I/HCl/O_2$ (0.03/0.03/85.0, P_T = 85.1 Torr) at 298 K. (a) Absorption spectrum before photolysis. (b) Spectrum of *syn*-*trans*-MVKO; taken from C.-A. Chung and Y.-P. Lee, Commun. Chem. **2021**, *4*, 8. Difference spectra recorded 0−5 μs (c), 5−10 μs (d), and 30−35 μs (e) after irradiation; negative bands of precursor are truncated. (f)−(h) Processed spectra of (c)−(e) with absorption bands of the precursor (*Z*)-$(CH_2I)HCC(CH_3)I$, spectrum (a), added back and those of MVKO, spectrum (b), subtracted. The region interfered with by absorption of the parent molecule (1140−1160 cm^{-1}) is shaded gray. Instrumental resolution is 1.0 cm^{-1}.

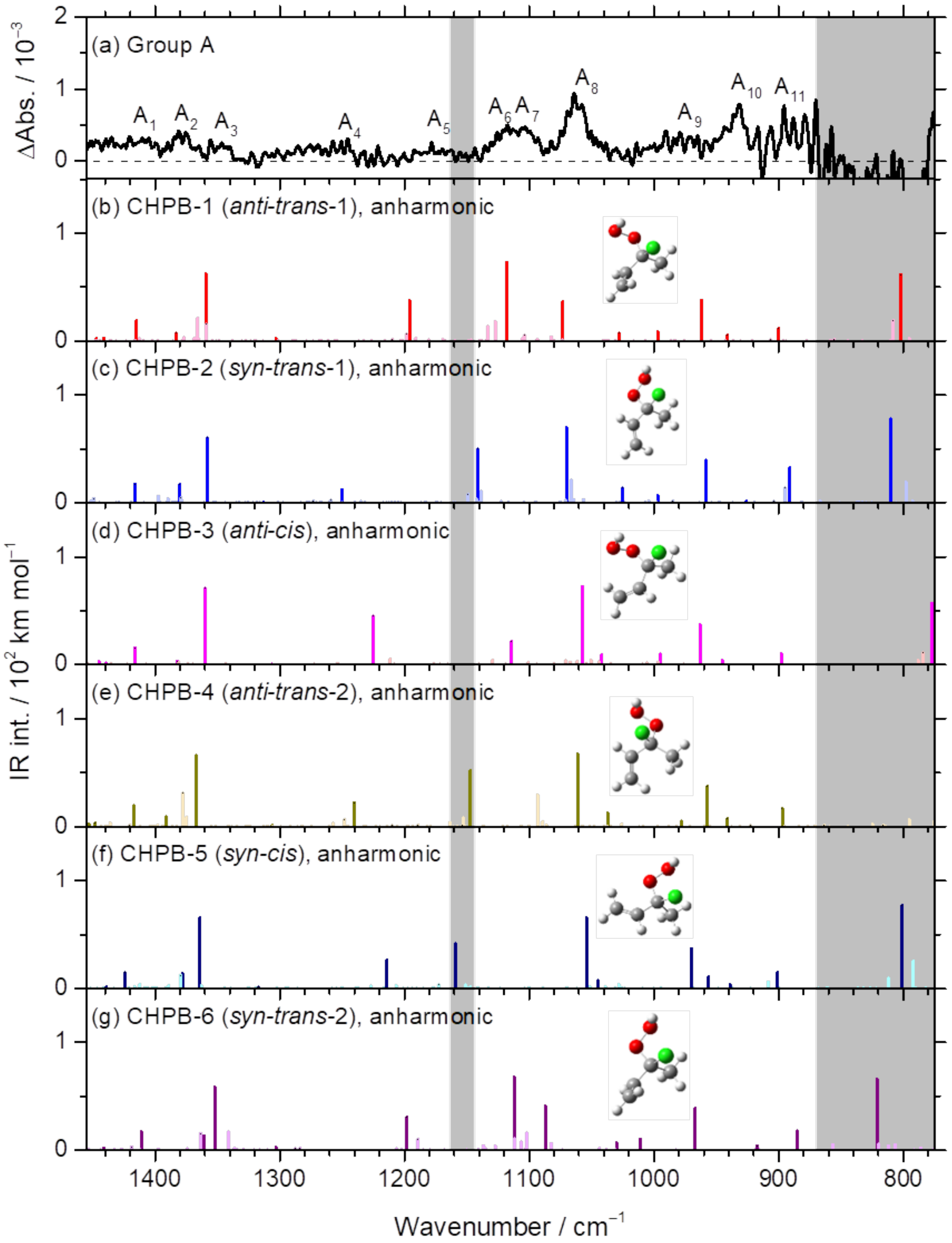

Figure S16. Comparison of bands in group A with anharmonic vibrational stick spectra of six conformers of CHPB. (a) Processed absorption spectrum of group A; instrumental resolution is 2.0 cm^{-1} ; taken from Figure 3g. IR stick spectra of CHPB-1 (b), CHPB-2 (c), CHPB-3 (d), CHPB-4 (e), CHPB-5 (f), and CHPB-6 (g) simulated according to anharmonic vibrational wavenumbers and IR intensities predicted with the B3LYP/aug-cc-pVTZ method. IR stick spectra of overtone and combination bands of each conformer are presented with light colors. The region interfered with by absorption of the parent molecule (1140−1160 cm^{-1}) and the region with poor S/N due to the cut-off of the filter (770−860 cm^{-1}) are both shaded gray.

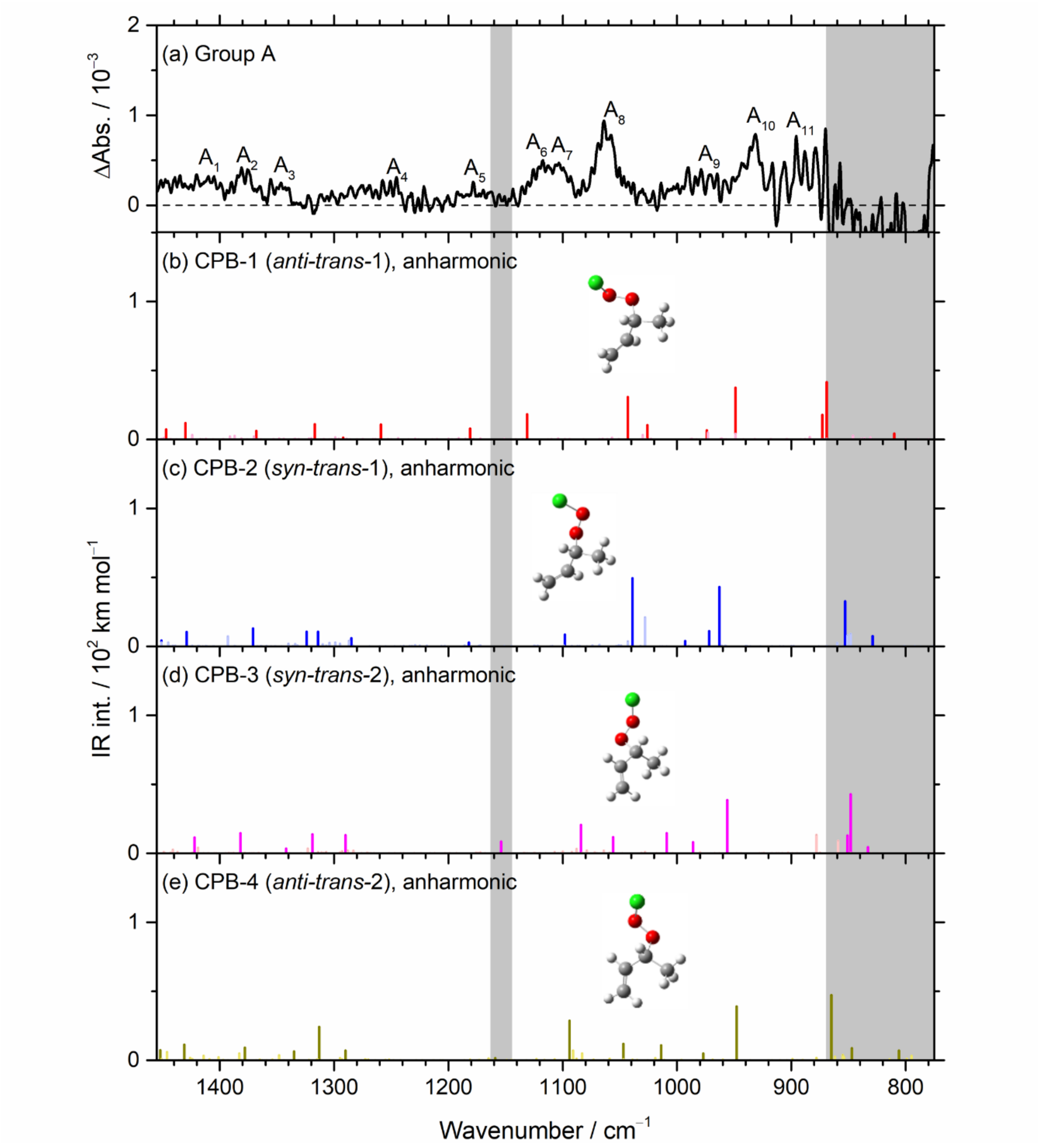

Figure S17. Comparison of bands in group A with anharmonic vibrational stick spectra of four conformers of CPB. (a) Processed absorption spectrum of group A; instrumental resolution is 2.0 cm^{-1} ; taken from Figure 3g. IR stick spectra of CPB-1 (b), CPB-2 (c), CPB-3 (d), and CPB-4 (e) simulated according to scaled harmonic vibrational wavenumbers and IR intensities predicted with the B3LYP/aug-cc-pVTZ method. IR stick spectra of overtone and combination bands of each conformer are presented with light colors. The region interfered with by absorption of the parent molecule (1140−1160 cm^{-1}) and the region with poor S/N due to the cut-off of the filter (770−860 cm^{-1}) are both shaded gray.

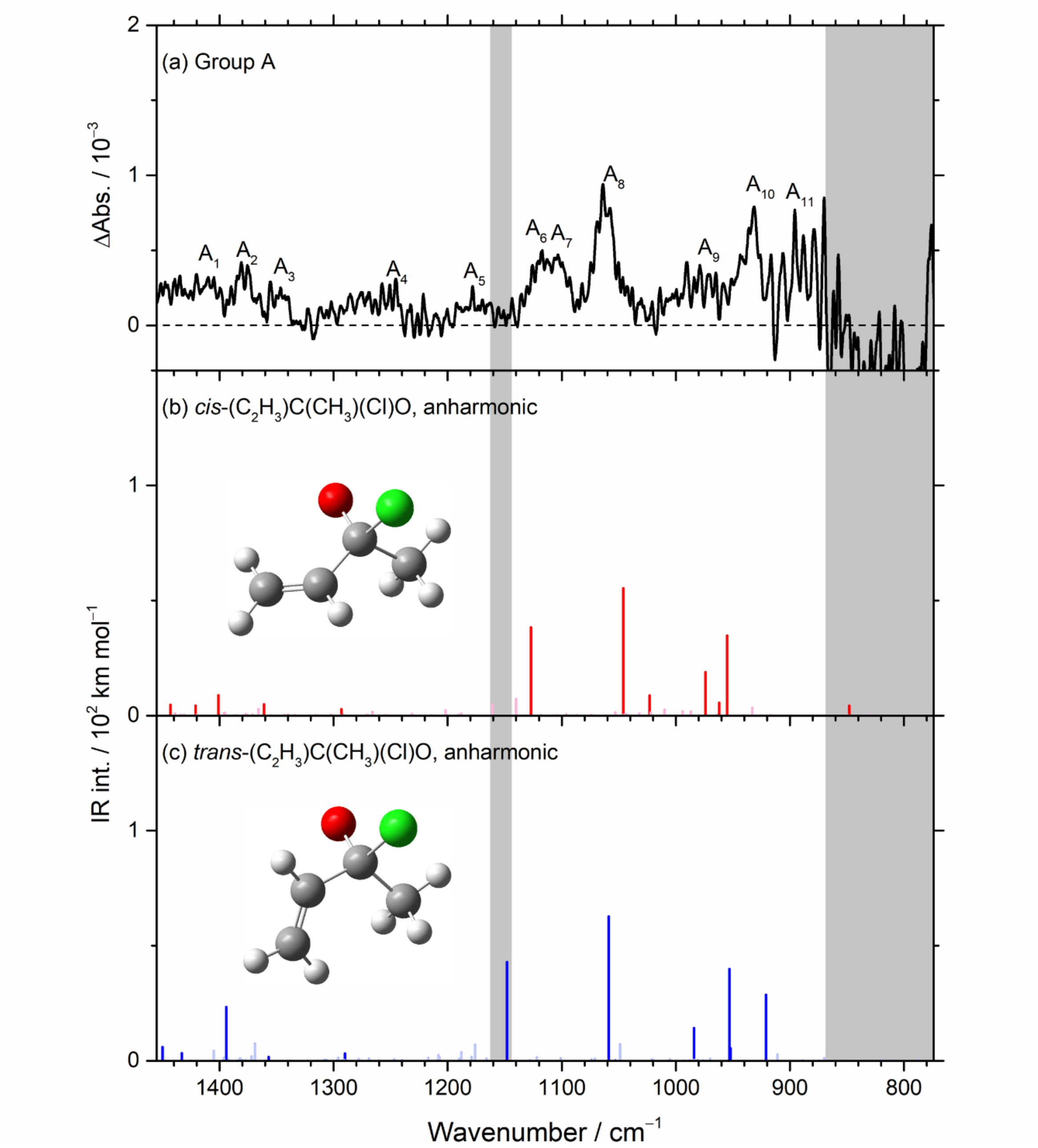

Figure S18. Comparison of bands in group A with anharmonic vibrational stick spectra of two conformers of $(C_2H_3)C(CH_3)(Cl)O$ (CMP). (a) Processed absorption spectrum of group A; instrumental resolution is 2.0 cm^{-1}; taken from Figure 3g. IR stick spectra of *cis*-CMP (b) and *trans*-CMP (c) simulated according to scaled harmonic vibrational wavenumbers and IR intensities predicted with the B3LYP/aug-cc-pVTZ method. IR stick spectra of overtone and combination bands of each conformer are presented with light colors. The region interfered with by absorption of the parent molecule (1140−1160 cm^{-1}) and the region with poor S/N due to the cut-off of the filter (770−860 cm^{-1}) are both shaded gray.

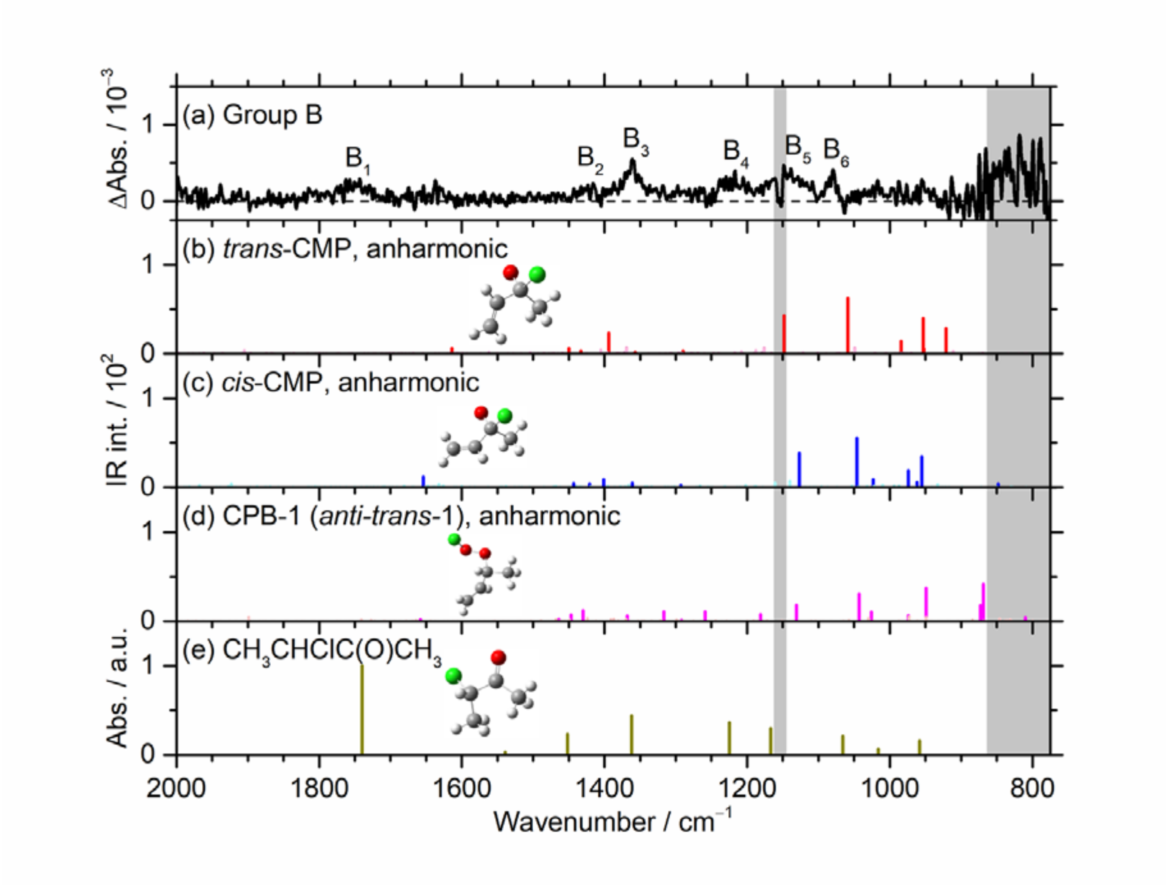

Figure S19. Comparison of bands in group B with IR stick spectra and absorption spectra of CMP, CPB and $CH_3CHClC(O)CH_3$. (a) Processed absorption spectrum of group B; instrumental resolution is 2.0 cm^{-1}; taken from Figure 3h. IR stick spectra of *trans*-CMP, $(C_2H_3)CCl(CH_3)O$, (b), *cis*-CMP (c), and CPB-1 (d) simulated according to scaled harmonic vibrational wavenumbers and IR intensities predicted with the B3LYP/aug-cc-pVTZ method. (e) Experimental absorption spectrum of $CH_3CHClC(O)CH_3$.[1] The region interfered with by absorption of the parent molecule (1140−1160 cm^{-1}) and the region interfered by the edge of the filter range (770−860 cm^{-1}) are both shaded gray.

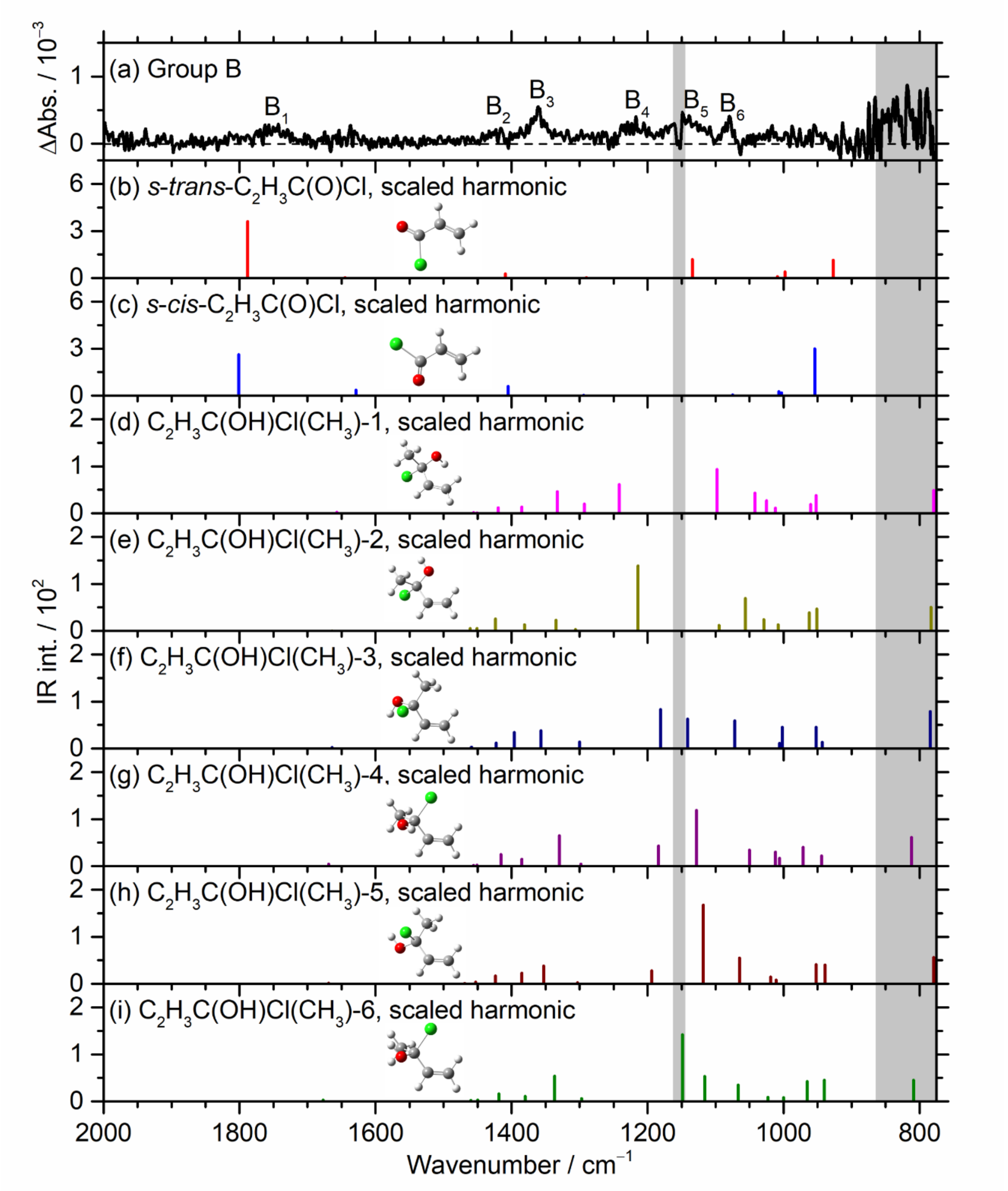

Figure S20. Comparison of bands in group B with IR stick spectra and absorption spectra of $(C_2H_3)C(O)Cl$ and $(C_2H_3)C(OH)Cl(CH_3)$. (a) Processed absorption spectrum of group B; instrumental resolution is 2.0 cm^{-1}; taken from Figure 3h. IR stick spectra of *s-trans*-$C_2H_3C(O)Cl$ (b), *s-cis*-$C_2H_3C(O)Cl$ (c),[2,3] and six conformers of $C_2H_3C(OH)Cl(CH_3)$ (d−i simulated according to scaled harmonic vibrational wavenumbers and IR intensities predicted with the B3LYP/aug-cc-pVTZ method. The region interfered with by absorption of the parent molecule (1140−1160 cm^{-1}) and the region interfered by the edge of the filter range (770−860 cm^{-1}) are both shaded gray.

References

[1] Sigma-Aldrich Co. LLC, Spectrum ID SLSVPFTIR_001111, IR spectrum of 3-chloro-2-butanone (CAS Registry Number 4091-39-8) obtained from SciFinder. Chemical Abstracts Service: Columbus, OH; https://scifinder-n.cas.org/searchDetail/substance/66cfdb737ae4e7512682263d/substanceSpectra, (Date accessed: 2024/8/29)

[2] Durig, J. R.; Berry, R. J.; Groner, P. Vibrational spectra and assignments, normal coordinate analyses, *ab initio* calculations, and conformational stability of the propenoyl halides, J. Chem. Phys. **1987**, *87*, 6303−6322.

[3] Das, P.; Lee, Y.-P. Infrared absorption of 3-propenonyl (·CH_2CHCO) radical generated upon photolysis of acryloyl chloride [$CH_2CHC(O)Cl$] in solid *para*-H_2, J. Chem. Phys. **2013**, *139*, 084320.
